# Seed Dormancy Involves a Transcriptional Program That Supports Early Plastid Functionality during Imbibition

**DOI:** 10.3390/plants7020035

**Published:** 2018-04-19

**Authors:** Alberto Gianinetti, Franca Finocchiaro, Paolo Bagnaresi, Antonella Zechini, Primetta Faccioli, Luigi Cattivelli, Giampiero Valè, Chiara Biselli

**Affiliations:** 1 Council for Agricultural Research and Economics—Research Centre for Genomics and Bioinformatics, via S. Protaso 302, 29017 Fiorenzuola d’Arda (PC), Italy; franca.finocchiaro@crea.gov.it (F.F.); paolo.bagnaresi@crea.gov.it (P.B.); zechiniantonella@virgilio.it (A.Z.); primetta.faccioli@crea.gov.it (P.F.); luigi.cattivelli@crea.gov.it (L.C.); giampiero.vale@crea.gov.it (G.V.); chiara.biselli82@gmail.com (C.B.); 2 Council for Agricultural Research and Economics—Research Centre for Cereal and Industrial Crops, s.s. 11 to Torino, km 2.5, 13100 Vercelli, Italy

**Keywords:** dry-afterripening, weedy rice, *Oryza sativa*, dormancy, germination, transcriptome, plastid

## Abstract

Red rice fully dormant seeds do not germinate even under favorable germination conditions. In several species, including rice, seed dormancy can be removed by dry-afterripening (warm storage); thus, dormant and non-dormant seeds can be compared for the same genotype. A weedy (red) rice genotype with strong dormancy was used for mRNA expression profiling, by RNA-Seq, of dormant and non-dormant dehulled caryopses (here addressed as seeds) at two temperatures (30 °C and 10 °C) and two durations of incubation in water (8 h and 8 days). Aim of the study was to highlight the differences in the transcriptome of dormant and non-dormant imbibed seeds. Transcript data suggested important differences between these seeds (at least, as inferred by expression-based metabolism reconstruction): dry-afterripening seems to impose a respiratory impairment onto non-dormant seeds, thus glycolysis is deduced to be preferentially directed to alcoholic fermentation in non-dormant seeds but to alanine production in dormant ones; phosphoenolpyruvate carboxykinase, pyruvate phosphate dikinase and alanine aminotransferase pathways appear to have an important gluconeogenetic role associated with the restoration of plastid functions in the dormant seed following imbibition; correspondingly, co-expression analysis pointed out a commitment to guarantee plastid functionality in dormant seeds. At 8 h of imbibition, as inferred by gene expression, dormant seeds appear to preferentially use carbon and nitrogen resources for biosynthetic processes in the plastid, including starch and proanthocyanidins accumulation. Chromatin modification appears to be a possible mechanism involved in the transition from dormancy to germination. Non-dormant seeds show higher expression of genes related to cell wall modification, suggesting they prepare for acrospire/radicle elongation.

## 1. Introduction

“Red rice” is the common name used for the heterogeneous group of the weedy rices, congeneric to crop rice and usually characterized by a red caryopsis [1]. These rices show various degrees of seed dormancy and can have much stronger dormancy than the cultivated rice [1]. From a physiological point of view, seed dormancy is considered to be the temporary failure of an imbibed and metabolically active seed to complete germination under otherwise favorable conditions [2]. A complex molecular network, not yet fully understood, regulates the induction and maintenance of seed dormancy, which is a general phenomenon present throughout the higher plants in all major climatic regions [3,4].

Although the dispersal unit (the structure by which the species disseminates) of rice is the spikelet, which in weedy rices shatters at maturation, physiological studies of seed dormancy often utilize the dehulled kernel (i.e., the caryopsis) to avoid interferences due to the hull [5,6]. It should be noticed that in such context the term “seed” is used in a wide, non-botanical sense and, in the present work, it refers to the red rice caryopsis.

Like in many species [3], red rice seed dormancy can be gradually released by dry-afterripening, that is, by storing the dry (actually, low-moisture; typically in the 6–14% range [7]) seed at non-freezing temperatures, usually 30 °C, for up to a few months [8] (at least 16 weeks are required to fully remove the dormancy of deeply dormant seeds [9]). Hence, when seeds are imbibed, their afterripening status determines their germination capacity and rate [8,10].

Germination is classically described as a sequential time course divided into three phases of seed water uptake [11,12]. The first phase is characterized by rapid seed imbibition, which is crucial for the transition from the quiescent metabolic state of the dry seed to the high metabolic activity of the hydrated seed. The second phase corresponds to a period during which the imbibed seed continues to absorb water though more slowly or its water content remains constant. In the third phase, rapid water uptake is resumed in concomitance with radicle, or acrospire, protrusion and seedling growth. In the dormant seed, the third phase is postponed, even indefinitely, and the seed persists in a metabolically active second phase. On the contrary, fully non-dormant rice seeds do not show a well-defined second phase as they germinate rapidly when they are imbibed at 30 °C [11] and their embryos quickly show an evident resumption of water uptake (third phase) in concomitance with the rupture of the pericarp [13]. A comparison of dormant and non-dormant seeds must therefore be accomplished before the third phase takes place for non-dormant seeds, to avoid comparing seeds that are at different developmental stages.

The first phase, corresponding to fast, passive imbibition, involves the resumption of general mechanisms for the start of metabolism and the repair of membranes and other cellular structures, and keeps reflecting an embryonic maturation program, including synthesis of proteins and metabolites for desiccation tolerance, until a certain developmental checkpoint turnover [12,14,15,16]. This suggests maintenance of the non-germination metabolism during the very first hours of imbibition, which opens a short decisional window for germination [17]. Seeds must reach a sufficient degree of imbibition before their metabolism and transcription can be thoroughly reactivated. In this first phase, degradation of several stored mRNAs, representing remnants from seed maturation, gradually starts [18,19]. In rice, these early activities are followed by large modifications in transcript abundances between 3 and 12 h of imbibition [20], a time interval that spans from the end of first phase through the second phase, which can be considered to start after 4 h (even though the seed can continue to slowly absorb water for several hours) [13]. A new transcriptional regulatory program is activated within the first 3 h of seed imbibition and it extends (through the first and second germination phases) to the first 9 h of incubation in barley [14] and to 12 h in arabidopsis [15] as well.

De novo transcription is not mandatory for early stages of germination, but it is necessary for the subsequent regulation of the germination rate and for seedling establishment [15,21,22,23]. Thus, in non-dormant seeds, the second phase, even though it takes place without visible morphological changes of the seed, is characterized by germination-specific changes that prepare the seed for radicle protrusion and seedling growth [18]. The second phase is therefore a suitable stage to study the differences that instantiate in the transcriptome of dormant and non-dormant seeds and make their fates to diverge. This can be done both at a sufficiently early time of imbibition, or by comparing the two types of seeds incubated under conditions that prevent the non-dormant seed to enter the third phase. A temperature below the minimum temperature for germination can be used for this purpose. Thereby, the presence of growing seedlings is prevented, and only imbibed seeds are compared.

Optimal temperature for germination of red rice is approximately 30 °C [9]. In this work, therefore, the transcriptomes of dormant (D) and non-dormant (ND) red rice seeds were compared after imbibition at 30 °C for 8 h, i.e., prior to any ND seed can attain pericarp splitting [5,13]. A further comparison between D and ND seeds incubated in water for 8 days (d) was set up at 10 °C, a temperature at which red rice seeds do not germinate [9]. The latter condition allowed to ascertain gene expression once regulation and metabolism were stabilized following imbibition. In this way, both comparisons allowed studying the differences in the transcriptome of ND vs. D seeds during the second germination phase, albeit in two different conditions, with 8 d at 10 °C being assumed to correspond not only to a different temperature, but also to a sufficiently long time of incubation in water to ensure that both water equilibration (seed imbibition) and metabolism had stabilized in the seeds. As additional controls, D seeds were also incubated 8 d at 30 °C and 8 h at 10 °C. The former test was aimed to establish how D seeds regulate transcription once D metabolism has stabilized at the normal temperature for germination. The latter control provided a reference for tracking transcriptional responses of D seeds to cold temperature, to distinguish them from responses really involved in the discrimination of the D/ND status.

In this work, a genome-wide transcriptional profiling was performed to investigate what differentiates D and ND red rice seeds at the gene expression level, and to compare these differences with findings from other species, with the aim to highlight common features that can help unravelling the general mechanism underpinning seed dormancy. Implied in this objective was the assumption that what we observe during incubation in water of D vs. ND seeds (before the second germination phase has ended) is the occurrence of the transition from dormancy to germination (at the expression level) that is consequent to the release (removal) of dormancy that has taken place during dry-afterripening. In fact, the transcriptional activity taking place before 6 h of imbibition does not determine whether seeds are able to germinate or not [23].

## 2. Results

Beside to basic informative data, this section shows an overall view of the findings as depicted by available bio-informatic tools such as PageMan and MapMan, whereas a more detailed picture, based on the biological functions of individual DEGs and on a careful re-construction of the related pathways as established according to the literature, is offered in the Discussion. An informed analysis of co-expression data, with its biological interpretation, is deferred to the Discussion too.

### 2.1. Germination Tests

The red rice genotype used in this work shows an almost categorical differentiation between D and ND seed, since D seeds do not germinate whereas, once afterripened, they become fully germinable (Table 1). This is a large vantage in terms of sharpness of discrimination and then of the findings, as compared to other studies wherein D seeds show partial dormancy or just a delay of germination with respect to ND ones. A slightly less sharp differentiation was observed only for D seeds incubated 8 d at 10 °C, which showed a small increase in germination capability when tested at 30 °C for two additional weeks (Table 1).

### 2.2. General Assessment of the RNA-Seq Results

The RNA Integrity Number (RIN, which represents a measure of RNA integrity) of ND seeds was consistently lower than that of D ones: the former ranged from approximately 6 to almost 7, whereas the latter from 8 to 9 (see Supplemental Figure S1 for typical plots). This difference is noticeable and is presumably due to two effects: first, ND seeds were obtained by dry afterripening the D ones for 16 weeks at 30 °C, which can cause some degradation of RNA (evident in terms of rRNA) that is not overcome neither in a few hours at 30 °C nor in a few days at 10 °C; second, by removing dormancy, a developmental switch is caused that will start germination, and a major transcriptional shift must occur accordingly, which requires degradation of unsuitable transcripts previously stored [20,24].

The whole set of reads (14,326,619) of one replicate of D seeds incubated at 30 °C for 8 d was aligned with the publicly available genome sequences of *Oryza* species, to ascertain the match of the studied red rice with the proper reference genome. In fact, not all red rices belong to *Oryza sativa* [25]. In the present case, however, the latter species gave the highest overall read mapping rate (97.5%; Supplemental Table S1), confirming that this red rice population belongs to *Oryza sativa* ssp. *japonica*.

As shown in Table 2, the overall number of expressed genes was found to be almost the same across all the conditions, corresponding to about one third of the estimated total number of genes in the rice genome (http://plants.ensembl.org/Oryza_sativa/Info/Annotation). Approximately one tenth of these expressed genes were annotated as non-coding transcripts. Since the methods used for RNA extraction and libraries construction were not suited to retain and detect microRNAs, these non-coding sequences have been retained because they might be either longer precursors of microRNAs or small RNAs or long non-coding RNAs. The complete set of genes queried in transcript profiling, with expression data for each contrast, is provided (Supplemental file “Expression_data_for_all_genes_in_all_conditions.xlsx”).

To make out the consistently most abundant mRNAs, the 100 transcripts with the highest level of expression were selected for every condition. They were first ranked within each condition according to the expression level and then their within-condition ranks (varying from 100 for the most highly expressed to 1 for the lowest) were used for a non-parametric Kruskal-Wallis test to ascertain whether there was any shift in the average ranking of each locus from an equal ranking of loci across conditions (which is the H_0_ hypothesis). When all the 100 transcripts from each condition were merged across conditions, the overall number of distinct loci rose to 186, meaning that some loci were among the 100 most highly expressed transcripts in some condition but not in others. As expected, the probability of a non-significant shift in the average rank of any locus was extremely low, assuming a Chi-square distribution with 185 df (Supplemental file “100 most abundant.xlsx”). Since this analysis aimed at identifying loci that were consistently most abundantly expressed across all conditions and that, therefore, might go undetected when differential expression analyses will be considered, only those loci (43 out of 186) that were found to encode for the most abundant mRNAs across all six conditions are reported in Supplemental Table S2. In general, several of the most abundant mRNAs were the same for all the conditions (Supplemental file “100 most abundant.xlsx”), although D seeds incubated in water at 30 °C for 8 d showed a quite evident rearrangement of the ranking for the very most abundant mRNAs (Supplemental Table S3). Thus, some genes were highly expressed independently of the seed condition and therefore were probably involved in functions of general importance for the seed. Nevertheless, it can be noted that many of the consistently most abundant mRNAs encode for storage proteins (Supplemental Table S2). This seems quite odd as these are all seeds that are not expected to accumulate storage proteins, but, rather, to utilize them either for germination or for survival during the rest of the imbibed seed in dormant state.

A large divergence of gene expression in ND seeds imbibed 8 h at 30 °C from all the other samples was observed (Supplemental Figure S2). However, both the overall number of expressed genes (Table 2) and the list of the very most abundant ones (Supplemental Table S3) were not affected. Since RIN values were quite low for ND seeds incubated either at 30 °C for 8 h or at 10 °C for 8 d samples (not shown), it seems that afterripening-caused RNA degradation was not the reason of this diversity. As it will be discussed, afterripening could rather have provoked damages to the cellular structures and this could have caused a transient increase in the expression of genes involved in metabolism restoration.

Expressed mRNAs from seeds incubated under the six different conditions were pair-wise contrasted to evidence relative changes in expression associated with the diverse conditions (Table 3). Table 3 indicates the intent of each comparison. The main interest focused on contrasting D and ND seeds. Even the comparison between D seeds incubated for 8 h (assumed to represent the inception of metabolism regulation) and 8 d (when seeds are assumed to be in a more stable physiological condition) was of major interest to understand how gene expression evolves from early to late dormancy. The other comparisons were mainly intended as controls ancillary to the understanding of the previously mentioned contrasts. For differential expression analyses, six pair-wise comparisons were therefore studied (Table 3): two contrasts between D and ND seeds (D 30 °C 8 h vs. ND 30 °C 8 h; D 10 °C 8 d vs. ND 10 °C 8 d), and four between D seeds incubated at different conditions (D 30 °C 8 d vs. D 30 °C 8 h; D 30 °C 8 d vs. D 10 °C 8 d; D 30 °C 8 h vs. D 10 °C 8 h; D 10 °C 8 d vs. D 10 °C 8 h). In each comparison, the level of expression in the latter condition was referred to the level of expression in the former condition.

Table 3 shows the number of DEGs for each condition. The most noticeable observation emerging from these data is that when gene expression of D and ND seeds was compared at 8 d of incubation (at a temperature that is necessarily non-permissive for germination, i.e., 10 °C) the number of DEGs plummeted. This would suggest that, once the seed metabolism has stabilized, most regulative and metabolic differences between the two physiological conditions vanish. If it were so, this contrast would be particularly interesting to pick out genes that are more directly involved in the differentiation of these physiological conditions. Indeed, the Principal Component Analysis (PCA) for overall gene expression of the experimental conditions with their replicates (Supplemental Figure S2) shows that gene expression in ND seeds is closer to that in D seeds for samples incubated 8 d than for samples imbibed 8 h (the total number of expressed sequences is, anyway, the same in the two ND samples; Table 2). Unfortunately, the plunge in the number of DEGs for this comparison appears to be largely due to a wider variability between replicates for these samples, which, however, may have a biological rationale (see Supplemental file “Insight into variability between replicates”).

### 2.3. Preliminary Assessment of Expression Profiles with PageMan

General expression profiles were preliminary compared by PageMan [26], a module of MapMan, which displays coordinated changes of functional classes of genes (“BINs” in MapMan terms [27,28]).

Dormant and ND seeds were compared, after incubating them for either 8 h at 30 °C or 8 d at 10 °C (Supplemental Figure S3). Given the low number of DEGs detected in the latter comparison, only a small number of DEGs could be contrasted across both paired sets; however, this was enough for some inference. A good correspondence of DEGs observed at 8 h at 30 °C and 8 d at 10 °C between D and ND seeds was found for stress, signaling and development, whereas photosynthesis-related genes were more expressed in D than ND seeds after 8 h at 30 °C, but they were more expressed in ND than D seeds after 8 d of incubation at 10 °C. As a general remark, we believe that DEGs that were consistently detected in both the comparisons (D 30 °C 8 h vs. ND 30 °C 8 h and D 10 °C 8 d vs. ND 10 °C 8 d) are of particular interest. Therefore, even though early differences in gene expression between D and ND seeds are expected to reflect more directly the initial determination of metabolism and regulation consequent to the diverse physiological states of the seeds, some features of transcription in the seed appear to be steadily associated with its dormancy status.

The effect of time of incubation in water (8 d vs. 8 h) was assessed for D seeds incubated at either 30 °C or 10 °C (Supplemental Figure S4). It can be immediately envisioned that photosynthesis-related genes, including those involved in the Calvin cycle were much more highly expressed at 8 h than at 8 d (at both 30 °C and 10 °C). When this finding is considered together with previous observation that, by 8 d of incubation at 10 °C, ND seeds restore their stock of photosynthesis-related transcripts (as described above, when commenting Supplemental Figure S3), it can be envisioned that whereas the expression of these genes was induced in ND seeds by 8 d of incubation (at 10 °C; Supplemental Figure S3), it was ultimately repressed in D ones following the initial surge. Overall, these results are consistent with a stabilization of metabolism and regulation in D seeds occurring by 8 d of incubation with respect to 8 h (at both 30 °C and 10 °C), as initially assumed. This confirms that although earlier changes in gene expression are more directly linked to the fate of the seed (to germinate or to stay dormant), later events can provide some additional clues on the physiological regulation of dormancy.

The effect of temperature (10 °C vs. 30 °C) was assessed for D seeds incubated for 8 h and 8 d (Supplemental Figure S5). It appears that many expression changes due to the diverse temperatures of incubation are specific to functional classes of genes (BINs) different from those that oppose dormancy to germination, and expression changes in gene families also affected in the comparison between D and ND seeds (like photosynthesis-related genes) were much less affected in this contrast. Therefore, there should not be relevant overall interference between changes in gene expression consequent to the temperature and the dormancy status, at least within the studied temperature range. Results obtained above for the comparisons between D and ND seeds across different temperatures can therefore be considered relatively safe with respect to this aspect.

### 2.4. DEGs Classification and Analysis

GO (Gene Ontology) enrichment analysis and MapMan software were used to identify the main functional classes of DEGs in each pair-wise comparison.

**Figure 1:**
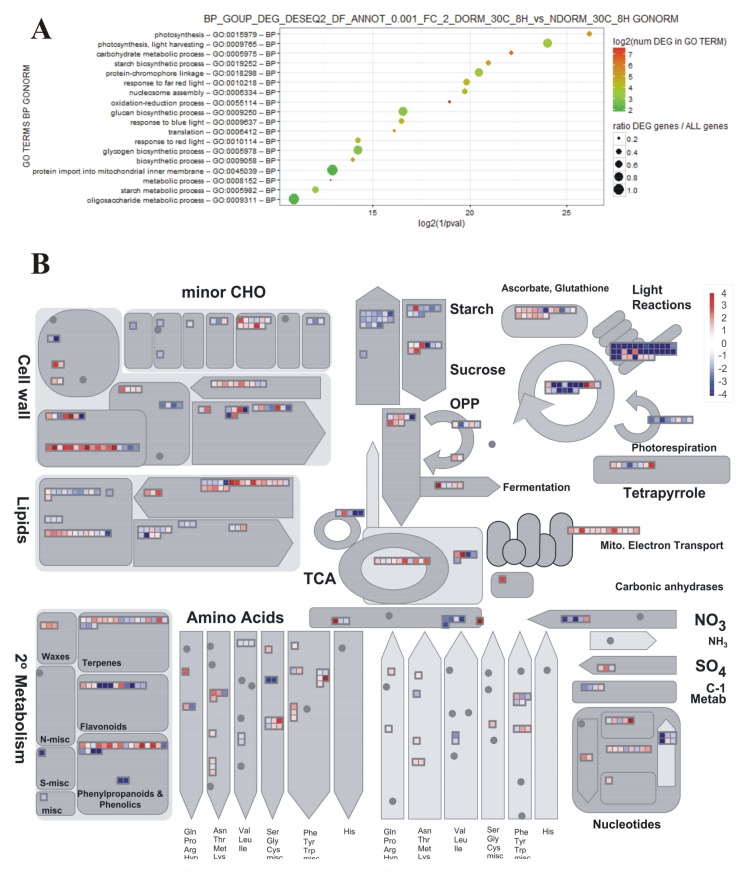
Comparison between gene expression in D and ND seeds incubated in water for 8 h at 30 °C. (**A**) GO term enrichment analysis for the Biological Process domain. The GO terms for which DEGs resulted enriched are reported on the y-axis, whereas the transformed GO enrichment probability is reported on the x-axis (higher values of the log_2_(1/probability value) transformation correspond to higher significance). The color of each spot indicates the number of DEGs that matched the GO term (log_2_-transformed), whereas the size of each spot shows the proportion of DEGs that matched the GO term with respect to all the rice genes that pertain to that GO term. (**B**) MapMan Metabolism overview. Single DEGs are represented by elementary squares grouped in functional BINs, and their color indicates the relative expression level in this contrast (in terms of log_2_FC, color scale on the right upper corner), with red showing relative higher expression in ND seeds and blue in D ones.

In the comparison of expression profiles of D vs. ND seeds incubated for 8 h at 30 °C, genes whose expression in seeds was most affected by their status of being D or ND were included in the following BINs (Supplemental Figure S6): photosynthesis (BIN 1), miscellaneous enzyme families (BIN 26), secondary metabolism (BIN 16), hormone metabolism (BIN 17), RNA processing (BIN 27), stress (BIN 20), development (BIN 33), not assigned loci (BIN 35), amino acid metabolism (BIN 13), and redox activities (BIN 21). GO term enrichment analysis and MapMan metabolism overview (Figure 1) evidenced the high expression of photosynthesis-related genes in D seeds, including genes encoding enzymes of the Calvin cycle and genes involved in other chloroplastic processes. Even the expression of several genes involved in the synthesis and polymerization of flavonoids was higher in these seeds. On the other hand, mitochondrial electron transport genes were more expressed in ND seeds, just as with enzymes for alcoholic fermentation. The BIN for cell wall modification showed an overall higher expression in ND seeds.

When the expression profiles of D and ND seeds were compared after 8 d at 10 °C, several functional classes of transcripts revealed an overall higher expression in ND seeds (Supplemental Figure S7), confirming that, oppositely to D seeds, quiescent (because of low temperature) ND seeds activated, or kept ready to activate, their metabolism rather than stabilize it. Whereas D seeds reduced the expression of photosynthesis-related (BIN 1) as well as of flavonoid-related genes as they remained metabolically active but dormant, ND ones reconstituted this set of transcripts that was apparently lacking during imbibition, particularly for light reactions and tetrapyrrole synthesis (protochlorophyllide reductase Os04g0678700). Therefore, for these genes, after 8 d at 10 °C the relative expression in D vs. ND seeds was reversed (i.e., from lower to higher) with respect to 8 h at 30 °C (Supplemental Figure S3).

**Figure 2:**
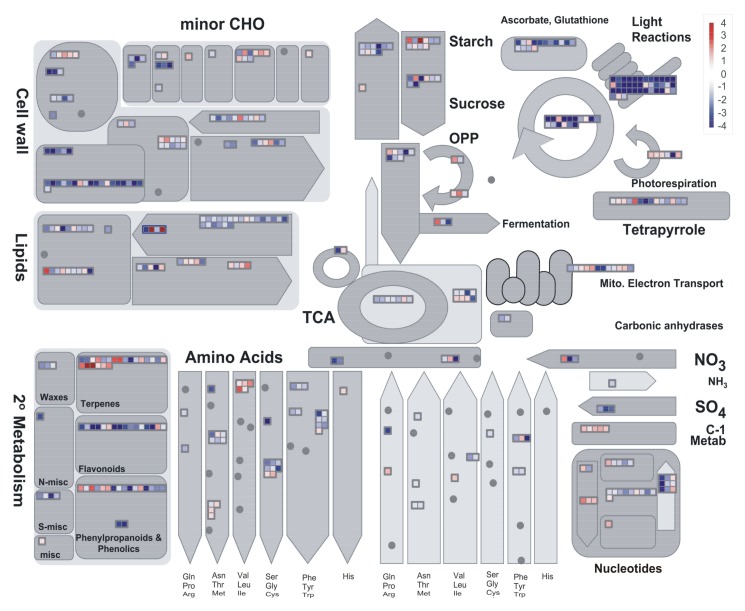
Comparison between gene expression in D seeds incubated in water for either 8 h or 8 d at 30 °C. MapMan Metabolism overview: single DEGs are represented by elementary squares grouped in functional BINs and their color indicates the relative expression level in this contrast (in terms of log_2_FC, color scale on the right upper corner), with red showing relative higher expression after 8 d and blue after 8 h.

In the comparison between D seeds incubated for 8 h or 8 d, it was observed that almost all transcripts for general metabolism had their expression decreased after more than one week of incubation at either 30 °C (Figure 2) or 10 °C (Supplemental Figure S8). This indicates a levelling down of transcription intensity in D seeds when their metabolic regulation stabilizes.

Only a few genes had relevant differences when expression profiles of D seeds were compared after 8 h of incubation at 10 °C with respect to 30 °C (not shown). One is pyruvate decarboxylase Os05g0469600, a key enzyme for fermentation that, therefore, appears to be more actively transcribed at 10 °C than at 30 °C.

Finally, when profiles of D seeds were compared after 8 d of incubation at 10 °C with respect to 30 °C, an overall increment of expression levels for metabolic activities was observed at 10 °C (Supplemental Figure S9). This should mainly represent an effect of adaptation to low temperature.

The responses of some representative genes were further confirmed by qPCR (Supplemental Figure S10). These genes were randomly chosen among DEGs and their expressions confirmed those obtained by RNA-Seq (see Supplemental file “Expression_data_for_all_genes_in_all_conditions.xlsx”).

### 2.5. Long Non-Coding RNAs

Several DEGs identified in the pair-wise comparisons were annotated as non-protein coding transcripts in the *O. sativa* Nipponbare IRGSP-1.0.27 genome release. Blast in the CantataDB (http://cantata.amu.edu.pl/) showed that several of these sequences match with at least one computationally identified long non-coding RNA (lncRNA). Loci without any known protein coding transcript were retained and examined for possible functions related to dormancy.

## 3. Discussion

Metabolism of imbibed caryopses necessarily diverges between D and ND seeds prior to the earliest time of germination of ND seeds (minimum time for a seed to germinate in water is approximately 9 h at 30 °C; see [5]), and commitment to one or the other path must be established and regulated before that time. Although gene expression represents only an indirect evaluation of how a biological system manages its metabolism, it offers an invaluable picture of how such system is preparing to change its metabolism and regulation. In the rice embryo, Howell et al. [20] found that, by considering a time lag (of some hours) between the transcript and metabolite changes, there was a good correlation between changes at the two levels. Moreover, the polysome occupancy of individual mRNA species is not affected by the seed dormancy status, indicating that differential regulation of translation in D and ND seeds mainly depends on transcript abundance [23].

In the present work, D and ND red rice seeds differed for many transcriptional switches. Likewise to what observed in wheat [24], several of them were associated with hormone metabolism and signaling, and similar to those found in arabidopsis [23] many were related to abiotic stress responses. However, differences in the expression of genes involved in the general metabolism were even more evident (Figure 1). In fact, reserve mobilization and several energetically costly processes associated with seed germination and preparation for subsequent seedling establishment are characteristically repressed in seeds in the imbibed D state with respect to ND ones [29].

### 3.1. The Impairing Effect of Dry-Afterripening

Although afterripening is closely associated with dormancy breaking, dormancy release and afterripening are distinct processes [4,10]. Indeed, dry-afterripening is a physical-chemical process that has a clearly negative effect on the stability of RNA (Supplemental Figure S1). It has thus been proposed that afterripening might increase germination potential by reducing levels of dormancy-promoting transcripts during dry storage [30]. This would take place because of differences in transcript stability, such that stable mRNAs would appear up-regulated following dry-afterripening because unstable transcripts, purportedly promoting dormancy, would appear to be down-regulated [30]. However, imbibed seeds do not show a correlation between mRNA stability and afterripening–dependent transcriptional regulation of the dormancy status [23,30]. An effect of global mRNA decay has therefore been excluded [23].

In addition, drying, by itself, alters the functionality of membranes and, therefore, of organelles; thus, in order to deal with the damage imposed during dehydration, dry storage and rehydration, seeds activate a number of repair mechanisms during imbibition [18]. This causes a hypoxic-like stress that induces some low-oxygen metabolic responses, such as enhanced ethanolic fermentation [31]. In many seeds, ethanolic fermentation is observed even during germination under normoxic conditions, indicating that the rate of pyruvate production (glycolysis) exceeds the capacity of the tricarboxylic acids (TCA) cycle and/or electron transport chain [31]. Long dry-afterripening, required to overcome red rice dormancy, apparently makes this problem worse, and thus ND seeds need to increase the transcription of key genes involved in the respiratory chain more than D ones (Supplemental Figure S11). This impairing effect of dry-afterripening on energy metabolism was clearly apparent as a hypoxic-like stress (see Supplemental file “Insight into the hypoxic-like stress caused by dry-afterripening”).

### 3.2. Nitrogen Metabolism

At 8 h of imbibition (both at 30 °C and 10 °C), D seeds showed a higher expression of plastid enzymes glutamine synthase (GS) and glutamate synthase (also known as GOGAT, i.e., glutamine oxoglutarate aminotransferase) with respect to ND ones (Supplemental Figure S12A). These enzymes are responsible for the incorporation of ammonia into amino acids by the so-called GS/GOGAT pathway. In seeds, ammonia used by GS to form glutamine is normally produced by the degradation of storage proteins [32]. Clearly, the latter process is very slow during imbibition and remains such indefinitely in D seeds. In fact, in rice, degradation of storage proteins mainly happens at the late stage of germination phase II [33]. Thus, the activation of the GS/GOGAT pathway in D seeds is not expected to be associated with notable storage protein degradation, but it could be involved in some other transamination process.

Red rice imbibed D seeds also showed slightly higher expression of alanine aminotransferases (glutamate:pyruvate aminotransferases) Os10g0390600 and Os09g0433900 (6.3-fold and 5.2-fold with respect to ND seeds, respectively, at 30 °C 8 h); the latter with a remarkably high expression level (Supplemental Figure S12A). Expression of Os09g0433900 as well as of its barley orthologous *qsd1* is embro-specific, and the barley alanine aminotransferase gene *qsd1* has been shown to be involved in the control of seed dormancy [34]. This kind of enzyme is usually induced during anaerobic stress, when alanine may serve as a storage form of pyruvate (perhaps in the vacuole) if pyruvate accumulation becomes excessive: alanine aminotransferases can act towards alanine accumulation when fermentation needs to be buffered, and they reversibly reconstitute pyruvate when it is depleted [34,35,36,37]. Thus, glutamate produced in the plastid could be used to aminate pyruvate to alanine, whereas the 2-oxoglutarate formed by de-amination of glutamate is continuously re-utilized by the GS/GOGAT cycle to re-synthesize glutamate [32,36]. Indeed, in germinating rice seeds, glutamate and alanine are the most abundant amino acids [38], and, in the shoot of germinating rice, there is a massive synthesis of alanine during anoxia, since alanine constitutes 30% and 50% of the total amino acids in rice roots and shoots, respectively, whereas it is only 6% in rice reserve proteins [32,39].

Based on these gene expression data, therefore, alanine production would be a preferred route in D seeds. It could be either a way to accumulate pyruvate while the mitochondrion is restored, or an aleurone/scutellum production aimed to transport amino acid units to the embryo axis, or it could serve to produce phenylalanine to form phenolics, or to transport amino acid units back and forth the plastid and the mitochondrion if the enzymes have diverse compartmentalization, or it might work with alanine:glyoxylate aminotransferase (which produces glyoxylate from glycine, and was more expressed in D seeds; Supplemental Figure S12A; specifically, its expression was 7.3-fold higher in D vs. ND seeds at 30 °C 8 h) to feed the glyoxylate cycle, which shall be discussed next.

Whatever the exact role of alanine is, it appears to be important in the maintenance of seed dormancy, as a mutation in an embryo-specific alanine aminotransferase strongly reduces seed dormancy in barley [34]. As the mitochondrion functionality appears to be more strongly impaired in ND seeds, however, use of alanine for pyruvate accumulation during mitochondrion restoration ought not to be a preferential feature of D seeds.

During rice germination, γ-aminobutyrate (GABA) is produced as early as 1 h of imbibition [20]. Higher expression of most genes for GABA metabolism was found in D seeds (Supplemental Figure S12B). Since alanine and GABA metabolism and accumulation are closely interconnected in rice [39], it can be speculated that, if there is indeed a net transfer of alanine between different tissues, the higher expression of alanine aminotransferases could also provide a quick equilibration between alanine flow and the TCA cycle by means of GABA:pyruvate transaminase and the GABA shunt in the mitochondrion.

### 3.3. Carbon Metabolism

The bulk of the reserves of cereal grains are stored in the dead starchy endosperm. However, the living aleurone cells of the endosperm, the embryo axis and scutellum also contain significant reserves in the form of oil and protein [40]. It is generally believed that triacylglycerol (lipid) reserves, present in dry and imbibed seeds as oil bodies (spherosomes or oleosomes), represent the principal energy and carbon stores within the embryo and aleurone, and are mobilized to sustain the embryo during early germination before the arrival of sugars from starch hydrolysis in the starchy endosperm can nourish the growing seedling [41,42].

In seeds, mobilization of lipid reserves typically takes place by means of the glyoxylate cycle, which, in conjunction with the TCA (tricarboxylic acid) cycle, provides the carbon skeletons used for biosynthetic processes through gluconeogenesis, that is, the generation of glucose, or, at least, of its precursor phosphoenolpyruvate, from non-carbohydrate organic substrates [43].

Although storage oil mobilization is not essential for seed germination, the ultimate fate of lipid-derived carbon in the embryo is committed to fuel some vital processes, and one of them could be chloroplast development [44,45]. This would explain why storage oil mobilization is essential for seedling establishment in arabidopsis [45]. In red rice, at 8 h and 30 °C, the high expression, particularly in D seeds, of some genes involved in the use of C4 organic acids for gluconeogenesis into the plastid (Supplemental Figures S11 and S12C), suggests that the plastid is provided with carbon skeletons (presumably from the glyoxylate cycle), which should then be used for biosynthetic processes. In fact, the prime functions of glycolysis in non-photosynthetic plastids are to participate in the breakdown of starch as well as to generate carbon skeletons, reductants, and ATP for feeding biosynthetic processes such as fatty acid and amino acid syntheses [46,47,48]. Correspondingly, in D seeds also genes encoding for enzymes of the plastid branch of glycolysis were much more highly expressed, namely, fructose-bisphosphate aldolase Os11g0171300 (whose gene expression increased 30.3-fold with respect to ND seeds, at 30 °C 8 h) and, particularly, NADP-dependent glyceraldehyde-3-phosphate dehydrogenase subunits A (Os04g0459500; showing 166.8-fold higher gene expression in D vs. ND seeds at 30 °C 8 h) and B (Os03g0129300; whose gene expression increased 84.9-fold) (Supplemental Figure S12D).

Genes encoding for enzymes of the glyoxylate cycle were highly expressed in both D and ND seeds (see Supplemental file “Insight into the carbon metabolism”), indicating that this cycle is important in imbibed seeds independently of their dormant status. However, in D seeds incubated at 30 °C for 8 h, a much higher expression of genes for enzymes of the plastid branch of glycolysis and the use of C4 organic acids for gluconeogenesis into the plastid suggests that the plastid is provided with carbon skeletons, which should then be used for biosynthetic processes. This would imply a quicker development of thylakoid membranes in the proplastid of D seeds, and indeed, higher expression of gene Os06g0563900 for diglyceride acyltransferase, which catalyzes the formation of triglycerides and is therefore presumably involved in the synthesis of membrane lipids, occurred in D seeds at 8 h of imbibition (see Supplemental file “Insight into the carbon metabolism”). As the embryo axis should not have an active glyoxylate cycle, the scutellum could then grant gluconeogenesis to replenish active biosynthesis in the axis (see Supplemental file “Insight into the carbon metabolism”). Differential expression of genes for the TCA cycle in D and ND seeds is consistent with a diverse regulation of primary metabolism in the two seed conditions (see Supplemental file “Insight into the carbon metabolism”).

### 3.4. Phosphoenolpyruvate Carboxykinase (PEPCK)

The glyoxylate cycle can feed gluconeogenesis with malate and, eventually, oxaloacetate, either directly or through the TCA cycle in the mitochondrion [43,45]. In any case, malate must be oxidized to oxaloacetate by malate dehydrogenase (which thereby reduces NAD^+^ to NADH) to allow gluconeogenesis to proceed from oxaloacetate to phosphoenolpyruvate by means of phosphoenolpyruvate carboxykinase (PEPCK, cytosolic). Indeed, PEPCK is a central element for the metabolic ability to mobilize storage lipids and proteins [44,49]. It predominantly allows soluble sugars to be made from C4 dicarboxylic acids produced by the breakdown of lipids [50] or even storage proteins [49], and the reaction it catalyzes is the major controlling step of gluconeogenesis [43,45]. In accordance with a preferential allocation of the carbon resources provided by the glyoxylate cycle to replenish gluconeogenesis for biosynthetic activities, PEPCK gene was more expressed in D seeds at 8 h of imbibition (at both 30 °C and 10 °C) and at 8 d of incubation in water at 30 °C (Supplemental Figure S12C); specifically, its gene expression increased 3.5-fold in D vs. ND seeds at 30 °C 8 h. In these seeds, phosphoenolpyruvate generated by gluconeogenesis could then be imported into the plastid to sustain biosynthetic processes.

### 3.5. Alanine Nutritional Shuttle

In the scutellum of germinating seeds, a relevant amount of the carbon skeleton produced by the glyoxylate cycle is used to form glutamate in maize [51], whereas alanine appears to be produced in barley [42]. This could well occur in rice too, since alanine represents a predominant amino acid during germination, especially under hypoxia [32,38,39]. As seen, dormant red rice seeds show high expression of alanine aminotransferase genes (Supplemental Figure S12A). Since both alanine aminotransferase Os09g0433900 and its barley orthologous are specifically expressed in the whole embryo [34], it might be supposed that alanine generated in the scutellum by alanine aminotransferases could then be re-converted to pyruvate, again by alanine aminotransferases, into the embryo axis, and pyruvate phosphate dikinases (gluconeogenetic enzymes highly expressed in red rice D seeds as well; Supplemental Figure S12C) could then phosphorylate pyruvate to phosphoenolpyruvate to start gluconeogenesis [50] in the embryo axis. Specifically, at 30 °C 8 h, expression of pyruvate phosphate dikinase gene Os05g0405000 (plastidic) increased 20.9-fold in D vs. ND seeds; whereas expression of pyruvate phosphate dikinase gene Os03g0432100 (cytosolic) increased 12.6-fold in D vs. ND seeds.

**Figure 3:**
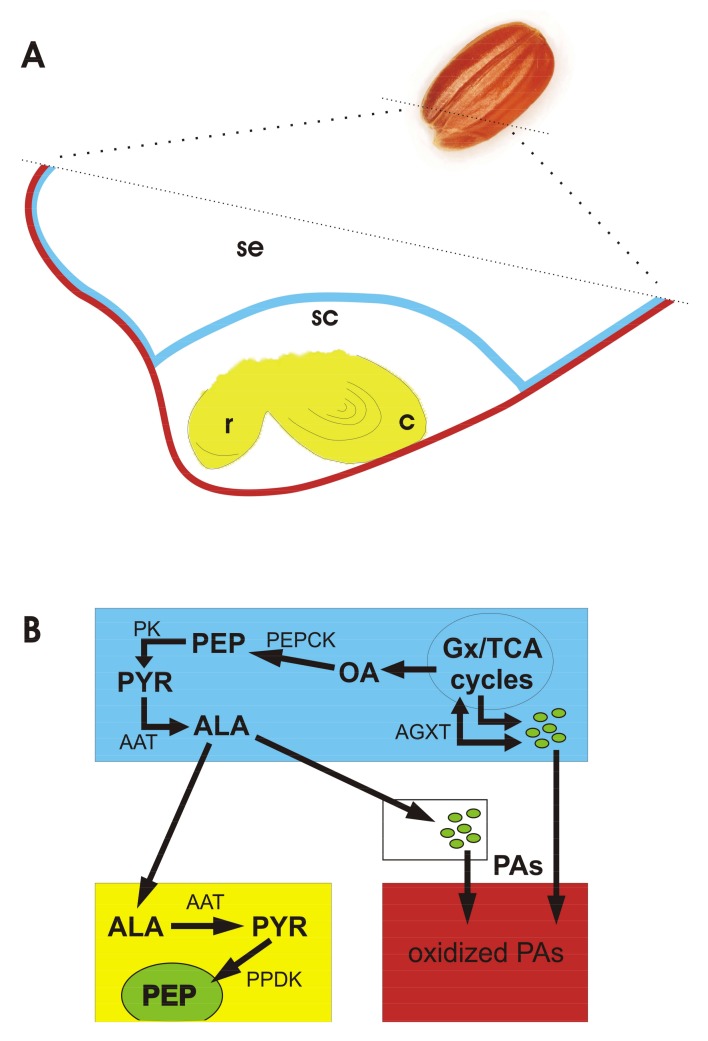
Proposed nutritional relationships in the D seed. (**A**) Representation of a section of the proximal region of the caryopsis. The caryopsis coat is red; the source tissues (scutellar epithelium and aleurone layer), which actively provide nourishment to the embryo by degrading reserves stored in the starchy endosperm (se) are represented by a light blue color (but they are colorless, in actuality); the embryo axis (i.e., the primordia of the radicle, r, and of the plumule, which is enveloped by the coleoptile, c) is highlighted in yellow (but it is colorless, in actuality). The embryo axis is embedded in the embryo collar, i.e., the grass hypocotyl that is expanded to form a bulging tissue and is fused with the scutellum (sc). (**B**) Schematic representation of the proposed nutritional relationships within the imbibed D caryopsis: rectangles correspond to compartments as colored in (**A**). The white rectangle represents the collar epithelium. Green ellipses represent proplastids (which are colorless, in actuality). In the source tissues, both storage reserves from the starchy endosperm and endogenous ones (soluble sugars/amino acids, oleosomes and protein bodies) can be utilized. Carbon skeletons undergo gluconeogenesis through the glyoxylate/TCA (Gx/TCA) cycles only in the source tissues. They are then transferred to the local proplastids for production of proanthocyanidins (PAs) and, through alanine (ALA), to the embryo axis, wherein they are, again, mainly used for biosynthetic processes in the proplastid. Supposedly, transferred reserves are used for PA synthesis in the collar epithelium too. Once released exogenously, PAs oxidize to the reddish pigments characterizing the caryopsis of red rice. AGXT, alanine:glyoxylate aminotransferase; OA, oxaloacetate; PEP, phosphoenolpyruvate; PYR, pyruvate; PK, pyruvate kinase; AAT, alanine aminotransferase; PEPCK, phosphoenolpyruvate carboxykinase; PPDK, pyruvate phosphate dikinase.

A much more widespread and persistent expression of a cytosolic pyruvate phosphate dikinase in the embryo than in the aleurone of germinated arabidopsis seed is, indeed, consistent with a specific role of this gluconeogenetic gateway in the embryo [50]. It seems therefore possible that alanine represents a nutritional shuttle from the scutellum to the embryo axis, since the glyoxylate cycle should be active in the former but not in the latter. This would explain why two mechanisms for gluconeogenesis seem to work at the same time: the first is based on cytosolic PEPCK producing phosphoenolpyruvate from the oxaloacetate coming from the glyoxylate/TCA cycles in the scutellum/aleurone [42,52], followed by formation of alanine [42,53] and, possibly, by its transfer to the embryo axis; whereas the second, based on pyruvate phosphate dikinases [50], would produce phosphoenolpyruvate from pyruvate generated from alanine by means of alanine aminotransferases in the embryo axis, where the glyoxylate cycle should not be active (Figure 3).

The latter reaction (i.e., production of phosphoenolpyruvate from pyruvate by means of pyruvate phosphate dikinase) also generates pyrophosphate, which is largely used as an alternative energy donor to ATP during an energy crisis [54]. Although high cytosolic concentrations of pyrophosphate could inhibit phosphoenolpyruvate formation, red rice D seeds at 8 h of incubation (at both 30 °C and 10 °C) also showed high expression of an H^+^-translocating pyrophosphatase membrane proton pump (Os05g0156900; Supplemental Figure S12E; specifically, its gene expression increased 212-fold in D vs. ND seeds at 30 °C 8 h) that, similar to that observed in arabidopsis [55], can suppress pyrophosphate accumulation and promote gluconeogenesis, while operating vacuolar acidification. Active H^+^ transport from cytoplasm to vacuoles is an important mechanism by which the cells regulate their intracellular pH, especially in hypoxic conditions, which favor cytoplasm acidification [36].

In addition, malate formed in the glyoxylate/TCA cycles can be exported to the cytoplasm, and eventually to the plastid, and then decarboxylated to pyruvate by NADPH malic enzymes (Supplemental Figure S12C), which thereby provide pyruvate for the pyruvate phosphate dikinases to produce phosphoenolpyruvate. This could entirely occur in the embryo axis if malate is provided by the TCA cycle, and it would require the intermediate formation (by transamination) and transfer of alanine, if malate is instead provided by the glyoxylate cycle in the scutellum, or aleurone, and then converted to pyruvate by malic enzyme [35]. Pyruvate used for gluconeogenesis, in the cytoplasm and in the plastid, could also directly come from glycolysis, which in the D seeds appears to be less liable to follow its anaerobic branch to ethanol.

An apparently reverse picture was depicted in arabidopsis, at the protein expression level, for pyruvate phosphate dikinases, PEPCK and alanine aminotransferases, whose up-accumulation was observed after dormancy breaking [29]. This can well be due to the later timing of observation of that study, corresponding to a progressive build-up of plastidial metabolism in ND arabidopsis seeds [29], as noted here for ND red rice seeds at 8 d of incubation at 10 °C. This would suggest that the PEPCK, pyruvate phosphate dikinase and alanine aminotransferase pathways have an important gluconeogenetic role associated with the restoration of plastid functions, both in D seeds, early after imbibition, as well as in ND ones, later.

Based on what has been discussed up to this point, it can be inferred that, at phase II of seed hydration, whereas the carbon metabolism predominantly flows toward cytosolic fermentation in the ND seeds, it proceeds toward some biosynthetic pathway(s) within the plastid in D ones.

### 3.6. Further Sugar Metabolism Features

Endosperm polysaccharide reservoirs are the most important carbon source for the embryo. However, α-amylases seem to become more important at later (8 d) rather than early (8 h) incubation times and at low temperature, with one form, Os02g0765400, more expressed in ND seeds, and another, Os08g0473600 (*Amy3E*), preferentially expressed in D seeds (Supplemental Figure S13A). Specifically, *Amy3E* expression showed a 3.5-fold increase in D vs. ND seeds at 30 °C 8 h and a 2.8-fold increase in D vs. ND seeds at 10 °C 8 d.

Following imbibition, the transcriptional activation of α-amylases is relatively late and quite slow, substantially increasing only when large-scale starch mobilization starts, i.e., just before radicle protrusion [56], which is consistent with a role of the biosynthesis of active GAs in the embryo and their transport to the aleurone layer in triggering the expression of α-amylase at the transcriptional level [57]. Dormant seeds also appear to preferentially express starch phosphorylases, which convert starch into glucose-1-phosphate that then enters glycolysis, with Os03g0758100 expressed early (5.2-fold higher expression in D vs. ND seeds at 30 °C 8 h), whereas Os01g0851700 more expressed later (8 d), particularly at 30 °C (2.3-fold higher expression at 8 d with respect to 8 h, for D seeds incubated at 30 °C) (Supplemental Figure S13A).

The scutellum and, eventually, the aleurone provide sucrose to the embryo axis, which then breaks down sucrose into hexoses by enzymes such as cell-wall invertases and sucrose synthases [54,58]. Expression of these and other genes for sugar metabolism are shown in Supplemental Figure S13B. Specifically, D seeds showed a much higher expression (375.1-fold with respect to ND seeds, at 30 °C 8 h) of Os04g0413500 (*GIF1*), encoding for a cell wall invertase [59]. On the other hand, sucrose synthases Os03g0401366 and Os03g0401300 (*SUS1*) and cell wall invertase Os02g0534400 (*OsCIN1*) were more expressed in ND seeds (with 7.6-fold, 3.3-fold, and 8.5-fold greater expression in ND vs. D seeds, respectively, at 30 °C 8 h). Sucrose synthase catalyzes the UDP-dependent cleavage of sucrose into UDP-glucose and fructose. In rice, transcription and translation of sucrose synthase is induced by hypoxia [36,37,54,60,61], though SUS1 increases in abundance even during normoxic rice seed germination [62] and a specific role in cell-wall synthesis in elongating tissues has been suggested for SUS1 [63]. In accordance, also Os01g0894300, encoding for fructokinase (which catalyzes the phosphorylation of fructose to fructose-6-phosphate that then enters glycolysis), was more expressed in ND seeds (2.1-fold at 30 °C 8 h). As for the invertase, over-expression of *OsCIN1* in seeds causes pre-harvest sprouting and may implicate a role for OsCIN1 in sugar-mediated α-amylases activation [59].

A transcript for fructose-bisphosphate aldolase (Os01g0905800), a key enzyme of the glycolysis pathway, was highly accumulated in seeds (Supplemental Table S2) under all conditions, though its expression was promoted at low temperature (Supplemental Figure S13C). Indeed, fructose-bisphosphate aldolase is predominantly, promptly and transiently synthesized in anoxia by rice coleoptiles [64]. This confirms that rice quickly activates glycolysis upon imbibition to support early energy-demanding biological process [20], just as observed with barley [14] and wheat [56]. As mentioned above, ND seeds appear to mainly use sugars for anaerobic glycolysis (fermentation). Accordingly, they show higher expression of fructose-bisphosphate aldolases Os05g0402700 and Os08g0120600 (Supplemental Figure S13C), and pyrophosphate-dependent phosphofructokinase Os05g0194900 (Supplemental Figure S13D), usually activated in anaerobic glycolysis [54,65]. On the other hand, D seeds appear to make a different use of sugars, as they preferentially express fructose-1,6-bisphosphatases Os01g0866400 and Os03g0267300 (Supplemental Figure S13E). Fructose bisphosphatase catalyzes the reverse of the reaction that is catalyzed by phosphofructokinase in glycolysis, that is, it converts fructose-1,6-bisphosphate to fructose-6-phosphate in gluconeogenesis and the Calvin cycle, which are both anabolic pathways. Correspondingly, ND seeds show higher expression of fructose-2,6-bisphosphatase Os11g0522000 (Supplemental Figure S13F). This enzyme catalyzes the production of fructose-2,6-bisphosphate from fructose-6-phosphate. The former metabolite activates pyrophosphate-dependent phosphofructokinase (=F6P1PT) and allosterically inhibits fructose-1,6-bisphosphatase, stimulating glycolysis while inhibiting gluconeogenesis [31], thus that high activity of one pathway is accompanied by low activity of the other. This is in agreement with previous observations that the level of fructose-2,6-bisphosphate normally increases in germinating seeds well beyond the level reached in D ones, which show, instead, an earlier and stronger enhancement of phosphoenolpyruvate and 3-phosphoglycerate levels [5,31,66]. In fact, high expression in D seeds of genes involved in gluconeogenesis into the plastid through the generation of phosphoenolpyruvate from non-carbohydrate organic substrates, specifically by means of pyruvate phosphate dikinase and PEPCK, suggests that phosphoenolpyruvate could indeed be preferentially accumulated in dormant red rice seeds. Analogously, as 3-phosphoglycerate can be used to transfer reducing equivalents into the plastid by means of the triosephosphate/3-phosphoglycerate shuttle [67], it could be preferentially accumulated in D seeds too, because of the biosynthetic processes apparently ongoing in the plastid. Although fructose-2,6-bisphosphate is not a universal marker for dormancy release [5], its prolonged accumulation, or, as presently observed, the accumulation of transcripts of its biosynthesizing enzyme, appears to characterize germination, at least under normal condition. This seems just to be a consequence of the shift of metabolism from glycolysis towards plastid gluconeogenesis in D seeds. One possible interpretation of this phenomenon is that both D and ND seeds suffer a hypoxic-like condition due to subdued activity of the respiratory chain, but whereas this stimulates anaerobic glycolysis in ND seeds, wherein quick energy surge is needed and excess NADPH must be consumed by alcohol dehydrogenase and alternative NADH dehydrogenases, excess reduced equivalents are extensively utilized in D seeds for plastid biosynthetic processes. In this sense, the previously mentioned preference for alanine production in D seeds could be due to the fact that fermentation to alanine by alanine aminotransferase does not contribute to the oxidation of NADH, as does lactate or ethanol production [32,37]: this can save more reducing equivalents that are then utilized in these seeds for plastid biosynthetic processes.

Furthermore, higher expression in ND seeds of genes for sucrose synthase, fructokinase, cytosolic fructose-bisphosphate aldolases, pyrophosphate-dependent phosphofructokinase and fructose-2,6-bisphosphatase, all known to be induced by hypoxia, provides additional support to the present contention that the long dry-afterripening used to obtain these seeds causes a stronger impairment in mitochondrial functionality that leads to a stronger hypoxic-like metabolism. The latter also seems to be enforced by incubation at low temperature.

### 3.7. Cell Wall Modifying Enzymes

The activity of cell wall enzymes plays a critical role in germination by enabling embryo cell expansion [18,68,69]. Many genes for hemicellulose remodelling are expressed during the early germination phase, and their encoded proteins are subsequently involved in loosening cell walls for cell expansion and division, and radicle protrusion [14,70].

Similarly to previous studies in rice [20,71] and barley [72,73], several genes for cell wall modification were up-regulated during imbibition (i.e., 8 h 30 °C) in ND, germinating seeds (Figure 1; Supplemental Figure S14A): expansins (like *OsEXPA2*, *OsEXPA4* and *OsEXPB6*; 4.3-fold, 23.5-fold and 22.8-fold, respectively), xyloglucan endotransglycosylases (like *OsXTR1*~*XTH2*; 15-fold), many pectin methylesterases (like *OsPME2*; 18.4-fold), hemicelluloses synthases, some β-xylosidases (like Os04g0640700 and Os11g0673200; 8.4-fold and 6-fold, respectively), endo-1,4-β-glucanase Os06g0256900 (8.2-fold), polygalacturonase Os01g0623600 (10.4-fold), and α-xylosidase Os01g0130400 (8.2-fold). On the contrary, cellulose synthases, some glycosyl hydrolases (like Os09g0520800 and Os04g0530700; 11.7-fold and 15.6-fold, respectively), some pectinases, pectin acetylesterase Os01g0892600 (37.4-fold), and invertase/pectin methylesterase inhibitors Os04g0587100 and Os03g0639400 (137-fold and 31.3-fold, respectively) were more expressed in D seeds (Supplemental Figure S14B).

Cell-wall modifying enzymes such as xyloglucan endotransglycosylase/hydrolases (XTRs/XTHs), expansins and endo-1,4-β-d-endoglucanases, together with plasma membrane proton pumps, are required for shoot cell growth [74], which also reflects in the expansion of embryo that brings about germination [69]. Specifically, early expression of *XTR*/*XTH* genes was observed during germination in different species [75,76,77], and the gene encoding for expansin OsEXPB6 is orthologous to the barley Contig7394_at that is up-regulated early in germination [14]. Expansins are plant cell wall-loosening proteins that stimulate wall polymer creep and, in general, exhibit extensive up-regulation during early germination [18]. Plasma-membrane proton pump (H^+^-ATPase) actively pumps protons from the cytosol into the apoplast and thus activates expansin activity resulting in cell wall loosening and cell expansion [74]. This can explain why transcripts for cell-wall modifying enzymes were more expressed in ND seeds together with some proton-transporting ATPases (like Os12g0168900 and Os04g0660600, 2.7-fold and 3.2-fold at 30 °C 8 h, respectively; Supplemental Figure S14C). Also pectin methylesterases [78,79] and polygalacturonases [80] have been shown to have a role in germination. Os01g0130400 is orthologous to the arabidopsis *XYL1*, which encodes for an α-xylosidase with a potential role in cell wall loosening and anisotropic growth [68].

Barrero et al. [73] showed that a number of cell wall degradation related genes are associated with breaking seed dormancy. In red rice, as reported above, transcripts for some cell wall modifying enzymes (e.g., OsEXPA4 and OsXTR1) that were more expressed in ND than in D seeds at 8 h at 30 °C were higher even at 8 d at 10 °C. Therefore, also given their consistent association with germination across diverse species, expression of these genes might represent a useful candidate marker of germination prior to any morphological marker such as pericarp splitting.

### 3.8. Proanthocyanidins and Phlobaphenes

As with most weedy rices [1], the red rice genotype studied in this work has a reddish-brown caryopsis at maturity [81]. In rice, the reddish-brown color of the caryopsis is due to the accumulation and oxidation of polymeric proanthocyanidins, sometimes called phlobaphenes [82]. Although the caryopsis coat has an important effect in maintaining the dormancy of the seeds in the long run [6], in normal germination tests, proanthocyanidins (PAs) have a small, though significant, effect on seed dormancy in rice [83].

**Figure 4:**
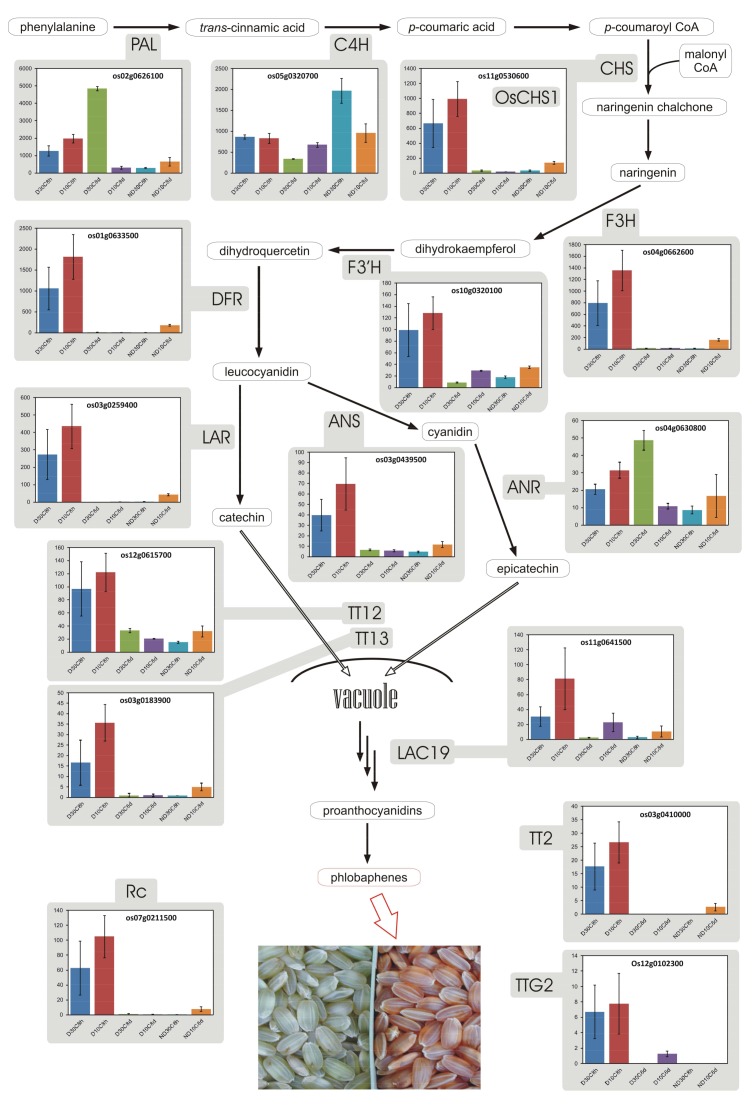
Expression levels (left scale on the y-axis of each plot) of DEGs involved in the proanthocyanidins biosynthesis pathway. PAL, phenylalanine ammonia lyase; C4H, cinnamic acid 4-hydroxylase (~ *trans*-cinnamate 4-monooxygenase); CHS, chalcone synthase; F3H, flavanone 3-hydroxylase; F3′H, flavonoid 3′-hydroxylase; DFR, dihydroflavonol-4-reductase; LAR, leucoanthocyanidin reductase; ANS, anthocyanidin synthase (~ leucoanthocyanidin dioxygenase, putative); ANR, anthocyanidin reductase; TT12, transparent testa 12-like protein (putative vacuole flavan-3-ol/proton antiporter involved in the transportation of proanthocyanidin precursors into the vacuole of the seed coat endothelium); TT13, transparent testa 13-like protein (putative tonoplast ATPase proton pump in the tonoplast of seed coat generating the driving force for TT12-mediated transport of PA precursors to the vacuole); LAC19, laccase (putatively responsible of the oxidative polymerization of flavan-3-ols); Rc, bHLH transcription factor (regulating proanthocyanidin production in seeds). In addition, two putative regulatory genes, not included in the list of DEGs because of their low expression levels and relatively high variance, but that could act as determinants in the accumulation of PAs in seed coat endothelium (owing to their similarity to arabidopsis genes having such function), encode for: TT2, transparent testa 2-like protein (a putative R2R3 MYB domain transcription factor); TTG2, transparent testa glabra 2-like protein (a WRKY transcription factor). The final evident outcome of this pathway is the reddish color of the seed coat, due to the oxidation of PAs to phlobaphenes (the image shows maturing red rice seeds at two stages: before and after PAs oxidation). Error bars represent standard errors (*n* = 3 repeats for each mean).

Many key structural genes involved in rice flavonoid biosynthesis have been characterized [84]. During imbibition (8 h), D seeds showed a coordinate expression of genes involved in the biosynthesis of those flavonoids that already confer to the seed its reddish color (Figure 4). Whereas such expression was higher than that of ND seeds at 8 h and 30 °C, after 8 d at 10 °C ND seeds showed a relatively higher expression, with respect to D ones, of some of these flavonoid genes. This apparent recovery is anyway tiny, as at 8 h (30 °C) genes for the synthesis of PAs were largely more expressed in D seeds (Figure 4). Higher expression of the cinnamyl alcohol dehydrogenase encoding locus Os04g0229100 (Supplemental Figure S15A) in D seeds during imbibition (8 h; specifically, a 18.3-fold stronger expression was observed in D vs. ND seeds at 30 °C 8 h), suggests that accumulation of PAs is accompanied by an increase of hydroxycinnamic acids, as indeed observed in rice [82].

In D seeds, the high expression, at 8 h, of transcripts involved in providing carbon skeletons to the plastid, presumably coming from β-oxidation in the glyoxysomes, could be also used for the biosynthesis of PAs. In fact, β-oxidation can have an essential role in inducing flavonoid biosynthetic genes [85]. In arabidopsis, PA biosynthesis intermediates accumulate in the vacuole thanks to tt13, a putative ATPase proton pump in the tonoplast of the seed coat generating the driving force for transport of PA precursors into the vacuole [86]. Although an orthologous to *tt13* is present in rice (Figure 4), as previously mentioned a tonoplast H^+^-translocating pyrophosphatase membrane proton pump (Os05g0156900) was also much more expressed in D seeds at 8 h of incubation (212-fold with respect to ND seeds at 30 °C; Supplemental Figure S12E), and it could provide the proton gradient necessary to drive accumulation of PA precursors in the vacuole in conditions of low availability of ATP, which should occur in the first hours of imbibition even for D seeds.

In maturing seeds, PA oligomers are probably polymerized, oxidized and deposited in the extracellular space of seed coat endothelial cells [86]. As some ATP-binding cassette (ABC) transporters are involved in the transport of flavonoids [87], it may be worthy to note that some ABC transporters, namely Os08g0544400, Os06g0503100 and Os02g0211000, were more expressed in red rice D seeds (11.2-fold, 9.4-fold and 8.1-fold with respect to ND seeds, respectively, at 30 °C 8 h; Supplemental Figure S15B).

### 3.9. Jasmonates

**Figure 5:**
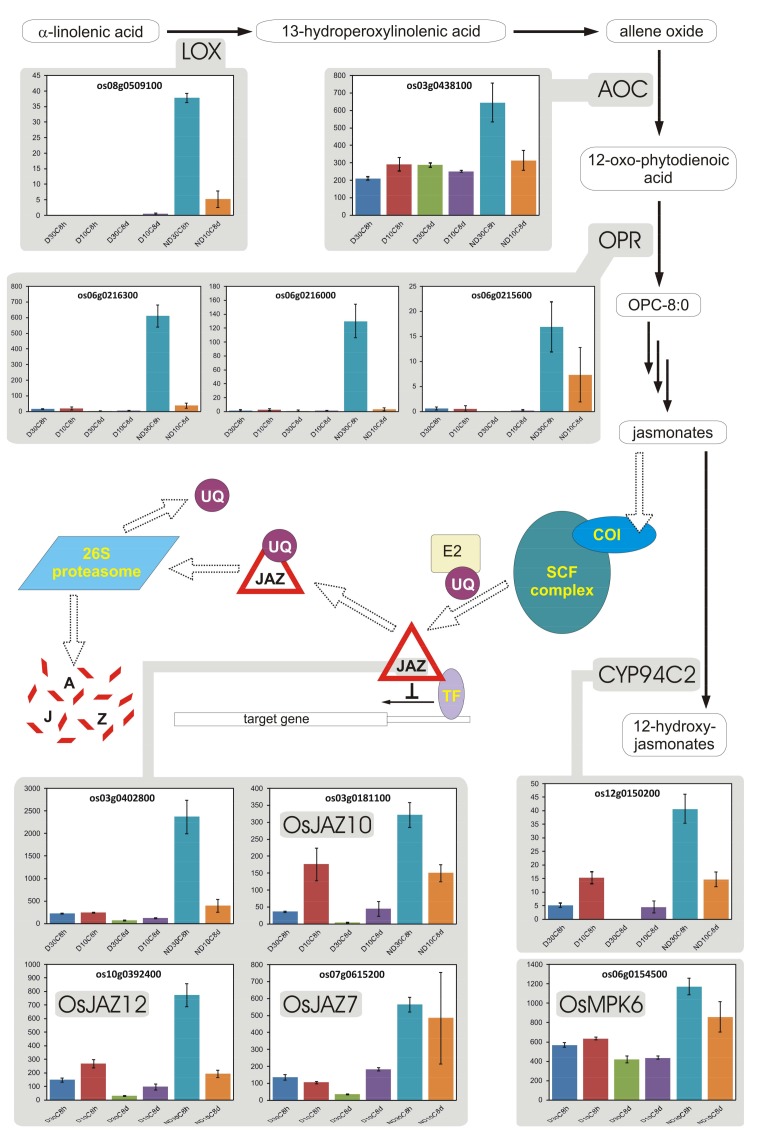
Expression levels (left scale on the y-axis of each plot) of genes involved in jasmonate (JA) metabolism and signaling. Metabolism of JAs (dark arrows): LOX, 13(*S*)-lipoxygenase; AOC, allene oxide cyclase; OPR, 12-oxophytodienoate reductases. These initial steps of the conversion of linolenic acid to 12-oxophytodienoate occur in the plastid. CYP94C2, a cytochrome P450 that inactivates bioactive JAs, probably the amino acid conjugate JA-isoleucine. JA signaling (white dotted arrows): bioactive JA (JA-isoleucine) is perceived by the F-box cofactor CORONATINE INSENSITIVE (COI), which activates an Skp/Cullin/F-box E3 ubiquitin ligase protein complex (SCF complex). The latter is thereby activated and specifies the ubiquitination (by multiple transfers of ubiquitin, UQ, from the ubiquitin-conjugating enzyme E2) and then the degradation of some transcriptional repressors of the family called JAZ (JA ZIM domain), via the 26S proteasome. If not degraded, JAZ repressors bind to a variety of cis-acting transcription factors (TF) in the promoter of JA-responsive genes and thus stop transcription of these genes. Degradation of JAZ proteins leads to the release of repression and expression of target genes. OsMPK6 is a MAP kinase involved in JA signaling and affecting plant defense and embryo development. Error bars represent standard errors (*n* = 3 repeats for each mean).

Jasmonates (JAs) are plant hormones that induce biosynthesis of many secondary metabolites involved in a variety of plant processes, including stress response [88]. Their specific effects depend upon the active transcription factors and repressors (JAZs, JA ZIM-domain-containing proteins). JAZs are constitutive repressors of JA-regulated transcription: in the absence of JA, JAZs repress transcription of target genes [88]. However, in the presence of JA, JAZs are degraded, allowing transcription factors to activate expression of genes needed in stress responses ([88]; Figure 5).

During imbibition (8 h) at 30 °C, ND seeds showed a higher expression of genes involved in JA biosynthesis, including a 13(*S*)-lipoxygenases and 12-oxophytodienoate reductases (Figure 5). On the other hand, also several JAZs were more expressed in ND seeds (Figure 5), and, at 8 h of imbibition at 30 °C, several stress transcripts, mostly related to biotic stress, were preferentially expressed in ND seeds (Supplemental Figure S15C). In addition, Os04g0650000 encodes for a cysteine protease involved in defense and much more highly expressed in ND seeds (14.5-fold at 30 °C 8 h; Supplemental Figure S15D), and is orthologous to arabidopsis *RD21* (At1g47128), which was reported to be associated with the germination potential [10] and the jasmonate signaling pathway [89].

Moreover, several APETALA2/ethylene-responsive element binding proteins (APT2/EREBP or APT2/ERF), which are implicated in the responses to both biotic and abiotic stress, were differentially expressed in D and ND seeds (Supplemental Figure S16). These genes participate in different pathways regulating stress-responsive networks in response to JA and other hormones, even independently of ethylene [90]. Ethylene, indeed, does not appear to be an hormone player here, because it is not produced in red rice D seeds as well as in ND seeds prior to germination [91]. Anyway, the ethylene signaling pathway was active, and some of its regulators (*OsEIN2* and *OsEIL2*, as well as *OsMED4*, a component of the complex central to transcriptional co-regulation of these genes) were differentially expressed (2.1-fold more expressed in D vs. ND seeds, and 2.8-fold and 2.2-fold more expressed in ND vs. D seeds, respectively, at 30 °C 8 h; Supplemental Figure S17A). Thus, JAs would seem to supplant ethylene in activating these genes in ND seeds prior to germination.

In addition, as JA production starts in the chloroplast or, in the case of seeds, in the proplastid, it is probable that ND seeds need to restore their basal level of JA, which was presumably depleted during the afterripening process. This can explain why most JA genes were strongly up-regulated in ND seeds during the first 8 h of imbibition (Figure 5). Indeed, growth is promoted by GAs that, however, repress defense gene activation in the absence of JA [92]. Thus, ND seeds presumably must re-synthesize JA to assure a balance between growth and defense. This seems to suggest that at least some early functions of proplastids are important in ND seeds too, and therefore the strong increase in expression of photosynthesis-related genes in D seeds at 8 h is devoted to maintaining some metabolic activity that is specific to such seeds, and not just the basic functionality of the proplastid.

Interestingly, both D and ND seeds expressed the gene for allene oxide cyclase (AOC in Figure 5), which produces 12-oxo-phytodienoate, but the ND seeds displayed higher expression of this gene while also showing very much higher expression of genes for 12-oxophytodienoate reductases (OPR in Figure 5), which are responsible of metabolizing 12-oxo-phytodienoate to JAs and were almost non-expressed in D seeds. As accumulation of 12-oxo-phytodienoic acid represses seed germination, at least in arabidopsis [93,94], it can be speculated that accumulation of 12-oxo-phytodienoic acid could indeed occur in D seeds, and this might have a role in maintaining their dormancy. Thus, the level of this compound deserves to be ascertained in red rice D and ND seeds.

Overall, our data suggest that afterripening activates the transcription of specific JA biosynthesis and signaling genes, particularly those encoding 12-oxophytodienoate reductase, though it is not clear whether this was a cause or an effect of dormancy decay and germination. These findings are consistent with those of Liu et al. [24] for wheat and of Barrero et al. [73] for barley. In barley, higher expression of a 12-oxophytodienoate reductase gene was specifically observed in the coleorhiza of afterripened seeds [73]. Finally, Linkies and Leubner-Metzger [95] noted that, in arabidopsis dry seeds, the non-dormant accessions are characterized by high JA contents, whereas the deeply dormant accession Cvi is characterized by low JA contents. This appears to agree with our expression data, and, when considered together with the literature, it suggests that, whereas D seeds accumulate 12-oxo-phytodienoic acid, germination is associated with JAs production.

### 3.10. Auxin

**Figure 6:**
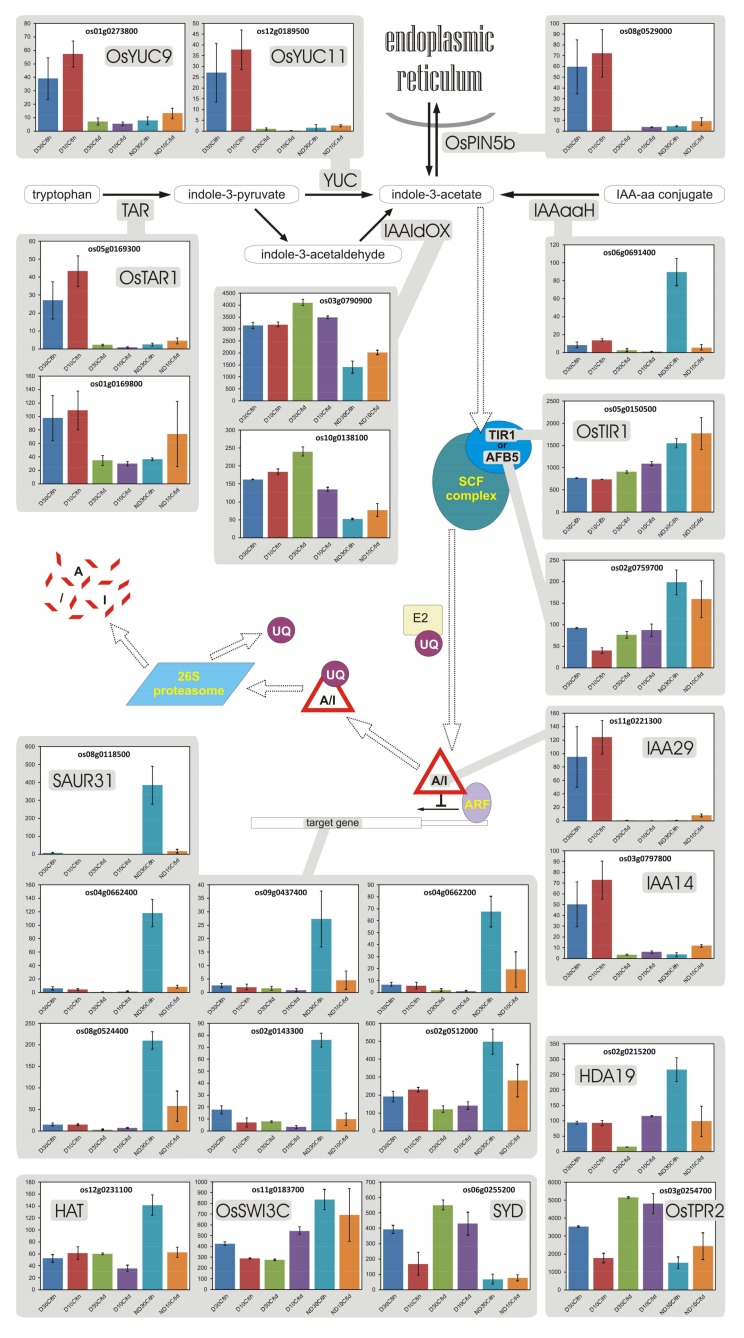
Expression levels (left scale on the y-axis of each plot) of genes involved in auxin metabolism and signaling. Metabolism of auxin (dark arrows): TAR, tryptophan aminotransferases of rice; YUC, flavin monooxygenases converting indole-3-pyruvate to indole-3-acetate acid (IAA), the main auxin in plants; IAAldOX, indole-3-acetaldehyde oxidases. OsPIN5b, auxin efflux carrier regulating auxin compartmentalization into the endoplasmic reticulum; IAAaaH, IAA:amino acid conjugate hydrolase. Auxin signaling (white dotted arrows): bioactive auxin (IAA) is perceived by an auxin signaling F-box cofactor, either TRANSPORT INHIBITOR RESISTANT 1 (TIR1) or AUXIN SIGNALLING F-BOX 5 (AFB5), each of which can be part of an Skp/Cullin/F-box E3 ubiquitin ligase protein complex (SCF complex). The latter is thereby activated and, depending on the cofactor, specifies the ubiquitination (by multiple transfers of ubiquitin, UQ, from the ubiquitin-conjugating enzyme E2) and then the degradation of some transcriptional repressors of the family called Aux/IAA (Auxin/Indole-3-Acetic Acid, here referred to as A/I), via the 26S proteasome. If not degraded, Aux/IAA (A/I) repressors bind to a variety of auxin response factors (ARF), i.e., cis-acting factors regulating transcription, in the promoter of auxin-responsive genes and thus stop the transcription of these genes. Degradation of Aux/IAA proteins leads to the release of repression and expression of target genes. The seven target genes showed here are putative auxin-responsive SAUR genes. Repression of auxin response genes can also involve histone deacetylase HDA19, which leads to a more compact chromatin state and thus prevents the expression of some auxin-responsive genes. The latter, however, is effective only when recruited, through TPR cofactors (like OsTPR2), by Aux/IAA (A/I) repressors in low-auxin conditions. Another class of chromatin regulatory proteins, the SWI/SNF chromatin-remodelling ATPases (like OsSWI3C), helps overcome this repressed chromatin state upon auxin sensing and in the presence of specific ARFs (like SYD), also by recruitment of histone acetyltransferase (HAT), which can then revert the compact/repressed chromatin state. Error bars represent standard errors (*n* = 3 repeats for each mean).

The main auxin in plants is indole-3-acetic acid (IAA [96]). Gene regulation by auxin is quite complex [97,98]. Contrary to what observed by Bai et al. [23] in arabidopsis, imbibed red rice D seeds (8 h at 30 °C) apparently had an increased expression of genes for auxin biosynthesis (the main pathway, from tryptophan to IAA, is considered here; also note that though tryptophan is synthesized in the plastid, auxin is synthesized in the cytoplasm), but they also showed increased expression of a putative intracellular carrier as well as of specific repressors (Aux/IAA) of IAA-responsive genes (Figure 6). On the other hand, ND seeds increased transcription of a gene linked to hydrolysis of inactive IAA:amino acid conjugates into active IAA, and they also showed higher expression of genes encoding for cofactors (OsTIR1 and AFB5) that enhance degradation of transcription repressors (Aux/IAA), thereby increasing the expression of target genes (Figure 6). As even the Aux/IAA themselves are target genes for this signaling pathway, at high IAA concentrations Aux/IAA proteins can block the expression of some specific genes otherwise activated by auxin, while others can fully express, essentially depending on cofactors.

Auxin regulation can also occur through chromatin remodelling by histone deacetylases, which reduce the accessibility of genomic DNA to transcription factors [97]. Aux/IAA proteins are involved in this repressing system together with other cofactors. Furthermore, this additional repressing system can be alleviated by histone acetyltransferases, if the latter are recruited by some cofactors and in the presence of specific auxin response transcription factors [97]. Interestingly, this mechanism can also repress JA-responsive genes [92]. Although in barley, genes for histones and chromatin structure are up-regulated in the late germination phase [14], red rice D and ND seeds displayed contrasting features as regards the expression of genes involved in this kind of repression already at 8 h of imbibition (Figure 6). To wit, HDA19, a histone deacetylase that increases chromatin compactness, preventing expression of some auxin-responsive genes, was overexpressed in ND seeds (2.8-fold with respect to D seeds, at 30 °C 8 h; Figure 6), and has indeed been shown to modulate seed germination [99]. However, histone acetyltransferase HAT and putative helicase SYD ought to cooperate in reverting the repressed chromatin state, but, though *HAT* expression pattern was close to that of *HDA19* (with *HAT* showing a 2.7-fold higher expression in ND vs. D seeds at 30 °C 8 h), *SYD* showed higher expression in D seeds (5.8-fold with respect to ND seeds, at 30 °C 8 h), suggesting these two latter genes are involved in differential regulation of gene expression in D and ND seeds.

Thus, on the one side, higher expression of genes for auxin biosynthesis [96,100] and gene *OsPIN5b* for an auxin efflux carrier [101] that has been suggested to serve as auxin receptor [98], were clearly associated with dormancy during imbibition (Figure 6). On the other side, D seeds also showed up-regulation of some Aux/IAA transcription repressors, whereas some cofactors enhancing degradation of the Aux/IAA repressors were up-regulated in ND seeds, thus several auxin response genes expressed in ND seeds were instead repressed in D ones (Figure 6). Apparently, auxin response is regulated at the level of individual genes and no clear-cut general transcriptional mechanism can be invoked for auxin signaling regarding seed dormancy.

Anyway, a large increase in IAA content during development of rice grains was shown to correlate with the expression of IAA biosynthesis genes *OsTAR1*, *OsYUC9* and *OsYUC11* [100]. Thus, over-expression of these genes in D seeds during imbibition (8 h) suggests that IAA may be important during dormancy, in addition to its previously suggested role early in grain development. In fact, auxin induces hypersensitivity of seeds to ABA and thereby inhibits germination, whereas afterripening induces transcriptional repression of specific auxin signaling genes [24].

Altogether, auxin seems to be somehow involved as an important regulatory hormone, at least for what concerns the transcription of specific genes in both D and ND seeds, but these seeds greatly differ in how auxin transcriptional regulation is performed and, then, in which genes are ultimately activated (Figure 6). Cofactors play a major role in determining this divergence [97], and cross-talking with other regulatory hormones is probably what really establishes the actual response in the two physiological conditions (D vs. ND).

### 3.11. Abscisic Acid

The role of abscisic acid (ABA) in seed dormancy is far from being clear [81,102]: ABA level does not appear to be correlated with dormancy level, although a minimal threshold of ABA is necessary; however, ABA sensitivity is clearly associated with dormancy. Besides, even if it enters the embryo, exogenous ABA is not able to restore dormancy in seeds wherein ABA synthesis has been blocked [81]. In fact, in many species, *sensu stricto* germination (testa rupture) of ND seeds is not prevented by ABA [18]. Correspondingly, the proteomic and transcriptomic profiles of D arabidopsis seeds differ from those of ND seeds treated with exogenous ABA to block their germination and growth, indicating that the mechanism of dormancy induction also differs [85,103]. Similarly, in wheat, it was observed that afterripening induces changes in the seed dormancy status without altering the dynamics of ABA metabolism [24].

**Figure 7:**
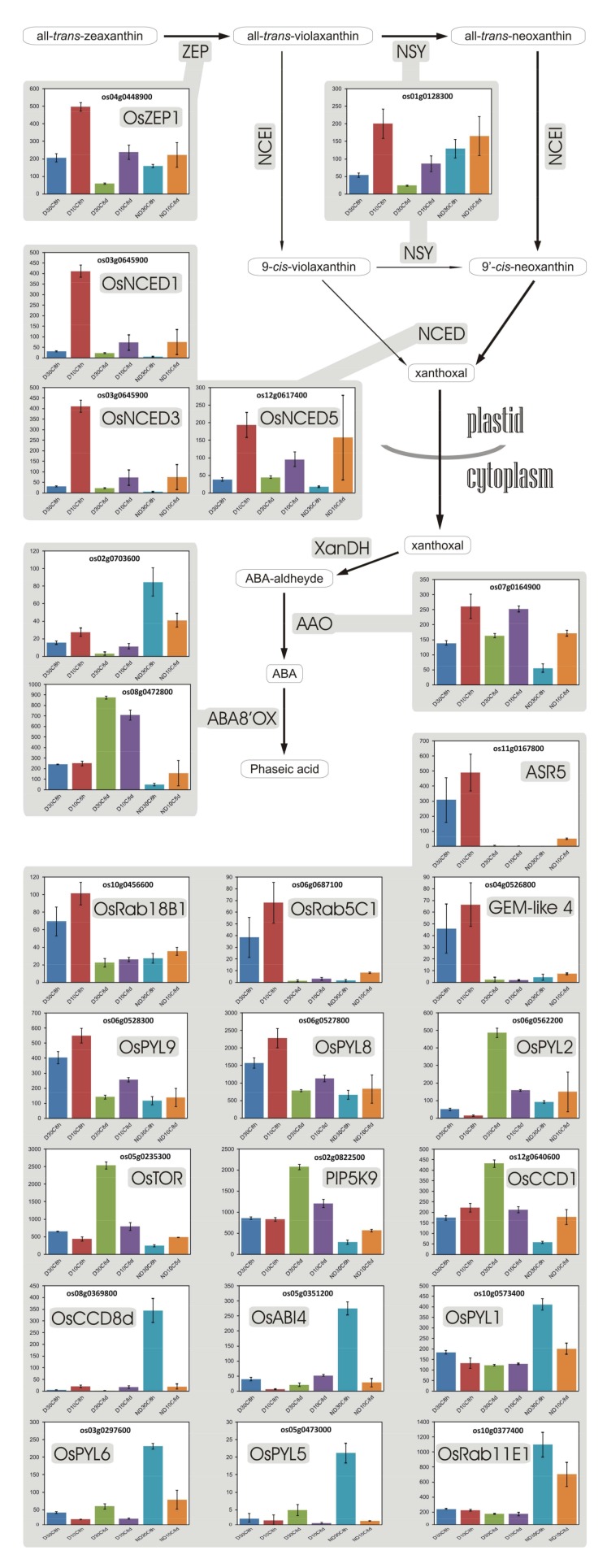
Expression levels (left scale on the y-axis of each plot) of DEGs involved in abscisic acid (ABA) metabolism, regulation and signaling pathways. Metabolism of ABA (dark arrows): ABA is produced in the plastid starting from carotenoids, with zeaxanthin epoxydase (ZEP) that catalyzes violaxanthin formation, a first important step for ABA biosynthesis in the seed; violaxanthin is then converted to neoxanthin by neoxanthin synthase (NSY), neoxanthin is further isomerized to its 9′-*cis* form by a still unidentified 9-*cis*-epoxycarotenoid-forming isomerase (NCEI). The 9′-*cis* isomer of neoxanthin is split by 9-*cis*-epoxycarotenoid-dioxygemase (NCED) to form xanthoxal (xanthoxin), which is then exported to the cytoplasm, where it is oxidized by xanthoxin dehydrogenase (XanDH) to abscisic aldehyde (ABA-aldehyde). Alternatively, as NCED appears to be able to form xanthoxal even from 9-*cis*-violaxanthin in vitro, it cannot be excluded that this latter can be directly formed by the action of NCEI, though 9′-*cis*-neoxanthin seems to be the typical substrate in vivo. ABA is formed by oxidation of the aldehydic group of ABA-aldehyde to the corresponding carboxylic group by ABA-specific aldehyde oxidase (AAO). ABA catabolism most commonly occurs through hydroxylation at the 8′ carbon by ABA 8′ oxidase/hydroxylase (ABA8′OX, cytochrome P450 monooxygenases of the CYP707A family) to form phaseic acid. The expression of a number of other genes, associated with ABA metabolism, regulation, or signaling, is shown too: an ABA-responsive GEM protein (GEM-like 4), a serine/threonine kinase controlling cell growth (OsTOR), a phosphoinositol-4-phosphate kinase (PIP5K9), two carotenoid cleavage dioxygenases (OsCCD1 and OsCCD8d), an embryo-specific AP2/ERF-domain transcriptional regulator (OsABI4), a soluble ABA receptor (OsPYL1), and three Rab GTPases (OsRab5C1, OsRab18B1 and OsRab11E1). Error bars represent standard errors (*n* = 3 repeats for each mean).

Expression of genes for ABA synthesis (Figure 7) changed more in function of the incubation temperature, that is, as a response to cold, rather than in relation to the dormancy status of the seed. Moreover, gene Os08g0472800 for OsABA8ox2 (~ OsCYP707A6), an ABA hydroxylase involved in ABA catabolism, was more expressed in D seeds (4.9-fold with respect to ND seeds, at 30 °C 8 h) even when metabolism had stabilized (4.5-fold with respect to ND seeds, at 10 °C 8 d), suggesting that ABA catabolism regularly happens in D seeds as well. Another gene encoding for an ABA hydroxylase, Os02g0703600 (*OsABA8ox1* ~ *OsCYP707A5*), which was more expressed in ND seeds (5.5-fold with respect to D seeds, at 30 °C 8 h), is orthologous to maize *ZmABA8ox1b*, which was proposed to contribute to seed germination by indirectly promoting cell expansion [104]. In rice, Os02g0703600 was indeed identified as a gene encoding a long-lived mRNA required for germination [105]. In this regard, it has been consistently suggested that low expression of *HvABA8’ox1* in barley [106,107,108,109] and of the gene encoding for ABA hydroxylase AtCYP707A2 in arabidopsis [106,110,111], which both are orthologous to rice *OsABA8ox1*, is important in the maintenance of dormancy and such expression increases during germination.

Curiously, D seeds, on average, showed a higher expression of gene *OsD27* for 9-*cis*/all-*trans*-β-carotene isomerase (Supplemental Figure S17B), suggesting that 9-*cis* carotenoids can have a role. As expression of genes *CCD7* and *CCD8*, involved in strigolactone synthesis together with *D27* [112], was very low in both D and ND seeds (not shown), expression of *D27* could be aimed to a different pathway. Maybe, as speculated by Bruno and Al-Babili [112], 9-*cis*-β-carotene produced by OsD27 is converted into 9-*cis*-violaxanthin via a route similar to the established pathway that leads to all-*trans*-violaxanthin and can thereby affect ABA synthesis. It remains to clarify why D seeds should prefer an alternative route to produce ABA.

Even for ABA, as with the other phytohormones, signaling is based on the repression of repressors of transcriptional activators. When ABA concentration increases, PYL/RCAR ABA receptors bind ABA and interact with, and thereby repress, protein phosphatases 2C (PP2Cs), which normally suppress activity of SAPKs (Stress/ABA-activated Protein Kinases; homologous to arabidopsis SnRK2s). This causes the release of SAPKs from the repression of PP2Cs, and as a result, SAPKs activate, by phosphorylation, ABA response element (ABRE) binding factors (ABFs), which are bZIP transcription factors that activate the expression of ABA-regulated genes [113]. At least three diverse pattern of gene expression are evident for PYL/RCAR ABA receptors (*OsPYL9* and *OsPYL8* vs. *OsPYL2* vs. *OsPYL1*, *OsPYL5* and *OsPYL6*; Figure 7), indicating that other factors intervened in determining the expression of these genes and then, presumably, the abundance of the receptors. ABA regulation seems therefore to be subject to, and thus mediate rather than decide, the dormancy status of the seed.

As for genes involved in signaling and response to ABA, on the one hand, imbibed (8 h) D seeds showed a neat higher expression of: an ABA/WDS induced gene (*ASR5*; with a 1036-fold higher expression in D vs. ND seeds at 30 °C 8 h); gene Os04g0526800, encoding for a putative ABA-responsive GEM protein (GEM-like 4; whose gene was 11-fold more expressed in D vs. ND seeds at 30 °C 8 h); and two *Rab* (Responsive to ABA) GTPases loci, *OsRab18B1* and *OsRab5C1* (showing 2.5-fold and 25.7-fold stronger expression in D vs. ND seeds, respectively, at 30 °C 8 h; Figure 7). Even some soluble ABA receptor genes, *OsPYL9* and *OsPYL8*, showed greater (3.4-fold and 2.4-fold, respectively) expression in D seeds (with respect to ND seeds, at 30 °C 8 h; Figure 7), with a pattern relatively similar to that of a gene encoding for a putative ABA responsive protein (Os02g0528300; Supplemental Figure S17C). A different pattern, with greater expression in D seeds incubated 8 d at 30 °C, was observed for regulatory genes *OsPYL2*, *OsTOR* and *PIP5K9*, as well as for carotenoid cleavage dioxygenase gene *OsCCD1* (Figure 7). A relatively similar pattern was also shown (Supplemental Figure S17D) by *OsMFT2*, a putative homolog of arabidopsis *MOTHER OF FT AND TFL1* (*MFT*) and of wheat *TaMFT*, which acts as an inhibitor of germination [114]; by *EIF4A*, encoding for a subunit of the eukaryotic initiation factor 4A that acts as an ATP-dependent RNA helicase unwinding mRNA secondary structures; as well as by *OsRad6*, encoding for a ubiquitin-conjugating protein E2, whose orthologous *VrUBC1* induced a highly sensitive response to ABA in terms of seed germination when over-expressed in arabidopsis [115]. Even the expression of gene *TCTP*, encoding for a translationally-controlled tumor protein that acts as a regulator of TOR [116], showed a corresponding pattern (Supplemental Figure S17D), and it was so high that *TCTP* transcript was one of the most abundant mRNAs (Supplemental Table S2). Notably, OsTOR is a conserved eukaryotic serine/threonine kinase that functions as a central controller of cell growth [117] and reduces sensitivity to ABA while increasing ABA synthesis and accumulation [118]. Specifically, rice lines overexpressing *AtTOR* were insensitive to the ABA-mediated inhibition of seed germination [117]. Thus, the high expression of *OsTOR* in D seeds after 8 d at 30 °C suggests that ABA-mediated inhibition of seed germination is not what keeps these seeds dormant, even though the role of TOR in seed dormancy can be different, as red rice D seeds do not show higher ABA but do show stronger ABA sensitivity [81]. Anyway, D seeds showed a slight but consistently higher expression of the *TRAB1* gene (2.3-fold in D vs. ND seeds at 30 °C 8 h; Supplemental Figure S17E), encoding for an ABF bZIP factor that mediates ABA signals to activate transcription [119]. This finding is consistent with a greater sensitivity to ABA in D seeds [81].

On the other hand, ND seed showed (mainly at 8 h and 30 °C) higher expression of genes encoding for: a carotenoid cleavage dioxygenase (OsCCD8d; 81.1-fold), an embryo-specific AP2/ERF-domain transcriptional regulator (OsABI4; 7-fold), soluble ABA receptors (OsPYL1, OsPYL5 and OsPYL6; 2.2-fold, 5.5-fold and 8.8-fold, respectively), and a Rab GTPase (OsRab11E1; 4.6-fold). ABI4 plays a central role in coupling metabolic status to the regulation of primary carbon metabolism [45] and is specifically expressed in seeds, whereas it is barely detectable in vegetative tissues after germination [120]. In arabidopsis, *ABI4* expression is confined to the embryo and accounts for the major differences in embryo response to ABA [121]. *ABI4* expression is the crucial determinant of the sensitivity of lipid reserve mobilization to ABA in the seed and is therefore associated with repression of lipid breakdown in the embryo during suboptimal conditions, such as osmotic stress [121]. ABI4 also represses nuclear genes involved in photosynthesis, fatty acid biosynthesis and pigment metabolism [121,122], all processes that, in accordance with *ABI4* expression, appear to be repressed in ND seeds and activated in D ones (at 8 h of imbibition), at least at the transcription level. In fact, ABI4 is not required for dormancy but is necessary for the ABA inhibition of germination [121]. Anyway, even though the role of ABA in the inhibition of chloroplast development in young seedlings is known, and occurs exclusively through the regulation of the nuclear genome [121], and ABI4 represses expression of several photosynthesis-associated nuclear genes, most likely this effect of ABI4 is independent of ABA signaling [122]. Indeed, ABI4 is a pivotal inhibitory element in the control of a complex regulatory network subject to a two-state master switch (on/off) for the coordinate expression of nuclear genes involved in plastid functionality [123,124]. Rab GTPases regulate structural membrane trafficking, including vesicle formation, vesicle movement along actin and tubulin networks and membrane fusion. Although it is known that different Rab proteins target vesicles to different membranes, the specific roles of the diverse plant Rab proteins is not well understood. However, proteins of the Rab11/Rab-A4 group are putatively involved in membrane addition at the growing tip [125]. Indeed, *OsRab11E1*, which belongs to the *Rab11*/*Rab-A4* group, was more expressed in ND seeds, wherein growth is supposed to be activated.

In relation to the previously mentioned dominating effect of the incubation temperature on the transcription of genes for ABA synthesis, it is worthy to note that the expression levels of four genes well-known to be specifically ABA-responsive, *OsEm*, *1Cys-Prx* and *SodCc2* [126] as well as *OsRab16A* (aka *RAB21* [127]), showed quite similar expression patterns (Supplemental Figure S17F). If these expressions were indeed responding to ABA level, they would consistently indicate that ABA level is more involved in the response to cold stress (10 °C) than in the dormancy status of the seed, in agreement with what observed for ABA biosynthetic genes. Expression of NCEDs genes, specifically, appeared to be responding more to low temperature than to the dormancy status of the seed (Figure 7). In fact, *OsNCED1* expression level correlates with ABA accumulation when rice plants are exposed to cold [128]. Incidentally, all the four ABA-responsive genes showed high average levels of expression, and the transcripts of three of them (*OsEm*, *1Cys-Prx* and *OsRab16A*) were among the most abundant mRNAs across all six tested conditions (Supplemental Table S2). Besides, OsEm increases the expression of other genes, including *OsLEA3-1* [129], which was among the most abundant mRNAs (Supplemental Table S2) and showed a similar pattern of the response to cold stress (10 °C; Supplemental Figure S17G).

Therefore, as judged from gene expression, ABA metabolism and signaling are certainly different in D and ND seeds, but overall differences are apparently not stronger, or even weaker, than those observed for other phytohormones. Accordingly, ABA was shown not to be involved in dry-afterripening regulation of gene expression in arabidopsis [10].

### 3.12. Gibberellins

In germinating cereal grains, gibberellins (GAs) are primarily synthesized in the embryo, particularly the scutellar epithelium, and are then relocated to the aleurone, where they induce the synthesis of hydrolytic enzymes to mobilize endosperm storage reserves to sustain embryo growth before an autotrophic phase is fully established [57,130]. Although GAs are not directly involved in the control of seed dormancy, they are classically known as germination-promoting hormones [131,132,133]. In cereals, however, the classical effect of GA induction of hydrolytic activities mainly occurs in the post-germination phase to support seedling growth [130,133], and even though GAs are required for the completion of germination, they are not involved in the initial mobilization of seed storage proteins and lipids [15]. 

**Figure 8:**
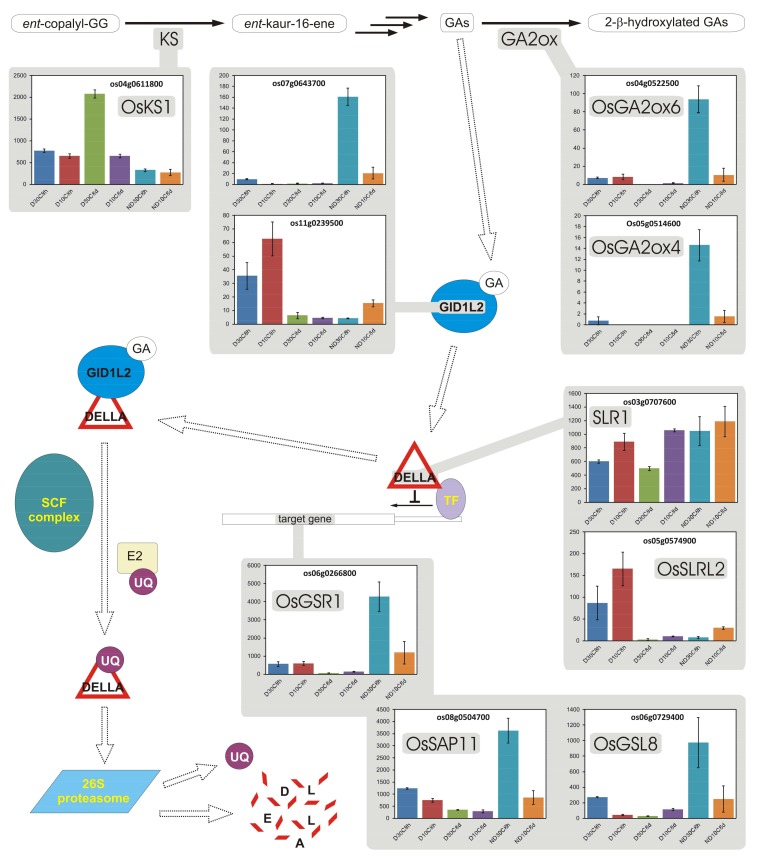
Expression levels (left scale on the y-axis of each plot) of DEGs involved in gibberellin (GA) metabolism, regulation and signaling pathways. Metabolism of GA (dark arrows): the first step dedicated to GA synthesis is catalyzed by ent-kaurene synthase (KS, plastidic); then, through several steps (in the endoplasmic reticulum and finally in the cytoplasm) bioactive GAs are produced. GA 2-oxidases (GA2ox) catalyze 2β-hydroxylation of GAs, which are thereby deactivated. GA signaling (white dotted arrows): bioactive GAs are perceived by GIBBERELLIN-INSENSITIVE-DWARF-1-like type-2 putative receptors (GID1L2), which then interact with specific transcriptional repressors called DELLA (like SLR1, or its functionally cognate GRAS factor OsSLRL2, which lacks the DELLA domain) and promote their ubiquitination (with multiple transfers of ubiquitin, UQ, from the ubiquitin-conjugating enzyme E2) by activating an E3 ubiquitin ligase protein complex (SCF complex). Ubiquinated DELLA proteins are then degraded via the 26S proteasome. If not degraded, DELLA repressors stop transcription of GA-responsive genes. On the other hand, degradation of DELLA repressors leads to the release of repression and expression of target genes (like *OsGSR1*, *OsGSL8* and *OsSAP11*). Error bars represent standard errors (*n* = 3 repeats for each mean).

In red rice seeds, gene *OsKS1* for the enzyme catalyzing the first dedicated step of GA synthesis was more expressed in D seeds (2.4-fold in D vs. ND seeds at 30 °C 8 h), with a particularly high expression after 8 d at 30 °C, and two genes for GA 2-oxidases (GA2ox), which are deemed to deactivate bioactive GAs [134], were more expressed in ND seeds after 8 h at 30 °C (13.4-fold and 16.8-fold with respect to D seeds for *OsGA2ox6* and *OsGA2ox4*, respectively; Figure 8). On the other hand, gene Os07g0643700 for a cofactor involved in relieving repression, by DELLA proteins [132], of specific GA-responsive genes, as well as some GA-target genes involved in germination (like *OsSAP11* and the ones encoding for putative GA-regulated GASA/GAST/Snakin proteins OsGSR1 and OsGSL8), were more expressed in ND seeds after 8 h at 30 °C (Figure 8). Interestingly, OsGSR1 activates the synthesis of brassinosteroids by directly regulating a brassinosteroid biosynthetic enzyme at the post-translational level, thereby mediating an interaction between GAs and brassinosteroids [135]. In addition, rice OsGSR1 is orthologous to arabidopsis AtGASA6, which has been suggested to be a positive regulator of seed germination that governs GA- and ABA-mediated seed germination via the action of AtEXPA1, a cell wall loosening expansin protein, by promoting cell elongation and consequently embryonic hypocotyls length [136]. Indeed, the rice α-expansin gene *OsEXPA4* is orthologous to arabidopsis *AtEXPA1* and showed a pattern of expression close to that of *OsGSR1* (compare Supplemental Figure S14A and Figure 8). In barley, Contig3674_at, orthologous to *OsEXPA4*, is up-regulated early in germination [14]. Other genes encoding for cell wall modifying enzymes showed the same pattern as *OsEXPA4* (Supplemental Figure S14A), suggesting they have a similar role and could be regulated by OsGSR1 as well. Among them, the expansin gene *OsEXPA2* is orthologous to *AtEXPA2*, whose expression was found to be specifically associated with germination in arabidopsis [10]. Transcripts of *AtEXPA1* and *AtEXPA2*, or their *Lepidium sativum* orthologouses, greatly accumulate during the early phase of seed germination in both *Arabidopsis thaliana* and *Lepidium sativum*, mainly in the endosperm, and are involved in ABA-insensitive processes that lead to testa rupture and germination [4,10,76,121,137,138,139,140].

Even for GAs, there seems to be no clear-cut overall behavior for the expression of biosynthesis and response genes with respect to the seed dormancy status. On the one side, this was expected, as the classical effect of GA induction of hydrolytic activities mainly occurs from the end of germination to the post-germination phase. On the other side, however, our results are consistent with a potential role of GAs, following imbibition, in activating cell expansion, as proposed for germination of seeds of dicotyledonous species [136,137,140]. If this latter effect is not associated with an increased synthesis of GAs in cereal grains early during imbibition, as inferred by the expression pattern of biosynthetic genes in red rice and by previous studies in barley [73,133], some specific changes in sensitivity and response could then play a role, mediated by genes that, as with *OsGSR1*, encode for positive regulators of GA signaling [135]. This would suggest that a dominating role can be played by GA signaling over GA biosynthesis in modulating seed germination and dormancy, at least in these species. This hypothesis would also explain why histone deacetylase gene *HDT701* was more expressed in red rice ND seeds (2.3-fold with respect to D seeds, at 30 °C 8 h; Supplemental Figure S17H), notwithstanding its overexpression has been shown to be associated with decreased histone H4 acetylation and consequent down-regulation of GA biosynthetic genes [141]; down-regulation that was observed here as well (Figure 8). Clearly, at present this represents an intriguingly but rather speculative interpretation, although what observed at the transcriptional level in red rice (this study) and barley [73,133] regarding the regulation of GA biosynthetic genes during imbibition seems worthy of note.

### 3.13. Seed Storage Proteins

Mature dry seeds contain large amounts of long-lived mRNAs that can contribute to protein synthesis during the early stages of germination [15,142], but also include other highly expressed mRNA species, such as the genes for seed reserve synthesis, for the translational machinery, for Lea proteins, and many others [14,16,138]. In barley, it has been shown that the endosperm of the germinating grain contains a considerable amount of residual mRNAs that are produced during seed development and are degraded during the early stages of germination [143]. In red rice, several of the most abundant mRNAs detected in imbibed caryopses across all the six tested conditions (Supplemental Table S2) encode for endosperm storage proteins.

In arabidopsis, seed storage proteins are stored in cotyledons, which are living organs, and their mRNAs are preserved in these organs in the dry seed, but are translationally arrested without being degraded, and become degraded only later, during germination [144]. Penfield et al. [121] hypothesized that unused transcripts stored in imbibed seeds may themselves be a stored seed reserve that is broken down so that the nucleotide components can be recycled for rapid de novo transcription during early postgerminative growth. It can therefore be hypothesized that, being the most abundant mRNAs, transcripts for endosperm storage proteins could represent a relevant form of nucleotide storage in the endosperm: they would not be dismantled until new mRNAs are to be synthesized, thereby avoiding to exceedingly enhance the concentration of unused, spare nucleotides in seed cells. This could therefore be the reason for which transcripts for storage proteins appear largely expressed in imbibed seeds, wherein they would otherwise seem unuseful.

Indeed, some specific transcripts appear to be stored in the seed and undergo controlled degradation upon imbibition [15,145], and an important role for mRNA decay during germination has been highlighted [145]. Accordingly, Howell et al. [20] noticed that the combination of a specifically timed up-regulation of a suite of specific transcripts and the degradation of stored mRNAs based on 3’ UTR sequences appear to be key elements in the coordination of at least some groups of transcripts during the early events in rice germination.

The presence of the AAAUAA motif in the 3′ UTR sequence has been shown to be involved in mRNA stability [146]. This motif (AAATAA, at the DNA level), is present in the 3′ UTR sequence of all the 13 highly abundant mRNAs encoding for prolamins, glutelins, albumins and globulins reported in Supplemental Table S2. This represents an almost five-times enrichment for the presence of the AAAUAA motif in this set of highly expressed transcripts for storage proteins (which is highly significant, with *p* < 0.0001, according to the one-sided Fisher’s exact test based on a 2 × 2 contingency table) with respect to transcripts not involved in the nutrient reservoir activity (represented by a random sample of 6258 protein-coding genes whose 3′ UTR sequence is identified in rice, which showed a presence of the motif in about 20% of the 3′ UTR sequence). The frequency of this motif in 91 genes representing all those classified in the class GO:0045735 “nutrient reservoir activity” for which the 3′ UTR sequence is presently identified in rice, is also higher (about 66%) than in the random sample of 6258 protein-coding genes (from which genes of this GO class were excluded). However, in this larger set, enrichment for the presence of the AAAUAA motif is only 3.26 (which is nevertheless still highly significant, with *p* < 0.0001, according to the one-sided Fisher’s exact test based on a 2 × 2 contingency table). The presence of this motif in many genes linked to “nutrient reservoir activity”, and, particularly, in all the 13 transcripts for storage proteins of Supplemental Table S2 indicates that the high levels of these mRNAs were indeed associated with their stability, which, in turn, confirms that they have some function that requires their persistence in the imbibed seed.

A detailed evaluation of the mRNA levels of several genes encoding for storage proteins (see Supplemental file “Insight into mRNA levels of seed storage proteins”), is consistent with the up-mentioned hypothesis that these transcripts could represent a form of nucleotide storage in the endosperm, and also leads us to propose that a strong differentiation between D and ND seeds in the apparent expression levels of some of these genes (specifically, genes for type B1 glutelins) can be explained by differential activation of mRNA turnover in D and ND seeds in the endosperm region next to the embryo.

### 3.14. Soluble Starch Synthase

Likewise, genes for seed storage proteins, *OsSSIIIa*, encoding for the soluble starch synthase IIIa, involved in generating relatively long starch chains in the endosperm, was consistently more expressed in D seeds (26.6-fold with respect to ND seeds, at 30 °C 8 h; Supplemental Figure S18A). As, like similar genes, it is mainly expressed in the endosperm, and, as with genes for seed storage proteins (see Supplemental file “Insight into mRNA levels of seed storage proteins”), is up-regulated by RISBZ1 (~ OsbZIP58 [147]), its apparent expression levels could undergo the same fate as transcripts of seed storage proteins and therefore similar considerations as discussed for their transcripts could hold for *OsSSIIIa* as well. However, the expression of this gene is evidently dependent on the dormancy status and not on the incubation temperature, though time of incubation is important too (Supplemental Figure S18A), and therefore it does not fit the previously proposed model for a storage form of nucleotides so well as transcripts for storage proteins. Thus, as the high expression in D seeds of some genes (Supplemental Figures S11 and S12C,D) involved in plastid gluconeogenesis suggests that the proplastid is provided with carbon skeletons, presumably from the glyoxylate cycle, for biosynthetic processes, it could even be supposed that some of these carbon skeletons can be used to produce starch in the proplastids. Indeed, during germination, soluble sugars taken up by the rice embryo can be transiently re-converted into starch [148] and accumulated as starch granules in the scutellar and embryo leaf sheath cells [21,149]. Accordingly, even genes for starch synthases OsSSI and OsGBSSI, as well as starch branching enzyme SBE1, were more expressed in D seeds (2.5-fold, 3.8-fold, and 2.5-fold with respect to ND seeds, at 30 °C 8 h; Supplemental Figure S18B). These three starch biosynthesis-related enzymes have been shown to be highly expressed at 24 h of rice germination and then to decrease at later stages [148].

Starch is synthesized by starch synthases starting from ADP-glucose: starch synthase elongates linear glucan chains by transferring a glucosyl unit from ADP-glucose and thereby also produces free ADP. ADP-glucose is produced by ADP-glucose pyrophosphorylase (AGPase) [150]. Some genes for rice AGPase subunits were correspondingly more expressed in D seeds at 8 h of imbibition (Supplemental Figure S18C).

In addition, D seeds at 8 h of imbibition, showed a high expression of *OsMADS29*, whereas no expression of this gene was observed in ND seeds at 30 °C 8 h (Supplemental Figure S18D). Overexpression of *OsMADS29* was found to mimic the effects of exogenous application of cytokinins that causes differentiation of proplastids to starch-containing amyloplasts and activation of genes involved in starch biosynthesis [151]. It could then be inferred that, upon imbibition, metabolism of ND seeds is directed toward making soluble carbon skeletons promptly available for growth; whereas, in the imbibed D seeds, sugars are rather partially accumulated in the proplastids, in accordance with the central role that these organelles appear to have in D seeds. Clearly, even this hypothesis needs further confirmations.

### 3.15. Pre-Emptive Defence Strategies and Regulation of Transcription

Many transcripts involved in response to stresses were differentially expressed in D and ND seeds (Supplemental Figure S6). A detailed evaluation of the mRNA levels of these genes (see Supplemental file “Insight into pre-emptive defense strategies”) reveals that the tuning of the pre-emptive defense strategy differs between D and ND seeds, in association with a diverse transcriptional regulation of JA and GA genes. Specifically, genes of the OsMKK4-OsMPK1-OsWRKY53 module, which stimulates production of lignin and oxylipins defense agents, were more expressed in ND seeds (2.6-fold, 2.1-fold and 3.9-fold, respectively, at 30 °C 8 h), whereas *OsNPR1*/*NH1*, putatively activating the biosynthesis of phytocassane antimicrobials, was more expressed in D seeds at 8 d of incubation at 30 °C (Supplemental file “Insight into pre-emptive defense strategies”). Besides, at 8 h of imbibition, D seeds showed a high expression of genes for PAs (Figure 4), whose major function is to provide protection against microbial pathogens, insect pests and larger herbivores [152]. The deposition of PAs in the endothelial layer of the seed coat in many species is a classic example of a pre-formed protective barrier [152]. Accumulation of PAs is an important defense strategy in seeds [153], and this seems to be especially true in red rice D seeds (Figure 3), beside to its effect in enforcing dormancy [6]. It is, therefore, possible to infer that different preventive protection systems are activated in D and ND seeds, presumably in function of their diverse expected fates: whereas ND seeds proceed toward germination and can successfully accomplish it only in the upper layer of soil, D ones persist buried in the soil, usually at greater depth, where a different set of pathogens, and insect herbivores, can pester them.

Only a few DEGs were consistently more expressed in D seeds than in ND ones over the two contrasts (D 8 h 30 °C vs. ND 8 h 30 °C and D 8 d 10 °C vs. ND 8 d 10 °C), among them two seem particularly interesting: *SYD*, encoding for a putative helicase involved in chromatin remodelling (Figure 6); and *OsRDR4*, for an RNA-dependent RNA polymerase (Supplemental Figure S19A).

On the one hand, SYD can be involved in auxin response (Figure 6) as it activates the expression of specific genes by releasing the compact, repressed chromatin state. *SYD* encodes a SNF2 protein, which belongs to a group of ATP-dependent chromatin remodelling complexes that are evolutionarily conserved and are involved in the control of essential growth and developmental processes in all eukaryotes [154,155]. Specifically, SYD is required for meristem maintenance [155]. In red rice, *SYD* expression pattern suggests it is involved in the activation of dormancy-specific gene transcription. Quite interestingly, the gene encoding for OsCAF1B, a putative CCR4-NOT transcription complex subunit involved in the regulation of mRNA deadenylation and degradation [156], was more expressed in ND seeds (8.2-fold with respect to D seeds, at 30 °C 8 h; Supplemental Figure S19B). Its pattern was almost opposite to that shown by *SYD*, thereby supporting that a mechanism for regulation of transcription is differentially switched in D and ND seeds. Expression of a gene orthologous to *OsCAF1B* was indeed negatively associated with dormancy in canola [157].

On the other hand, OsRDR4 should be involved in RNA interference and silencing: RNA-dependent RNA polymerases (RDRs), in fact, are the core proteins mediating RNA interference as they can amplify microRNAs and small temporal RNAs and can also produce double-stranded RNA using small interfering RNAs as primers [158]. In addition, RDRs have antisense RNA synthesis activity independent of the endogenous small RNA pathways [159]. Quite interestingly, *OsRDR4* expression appears to be quite specific, as it does not show detectable levels in vegetative tissues except the shoot apical meristem [158].

Since dormancy blocks the development, it can indeed be expected to be associated with the remodelling of chromatin structure. It is enticing, anyway, that a specific RDR is preferentially expressed in D seeds. This would suggest that in D seeds some, or several, transcripts involved in germination are not repressed, but are just silenced. It could be a way to keep ready for germination in case quick resumption of development were required, similar to what happens after wounding [91]. Overall, an important role during germination has been suggested for both the regulation of transcription levels of dormancy genes by chromatin remodelling [14,17,160] and antisense RNA production [161]. Our findings confirm the simultaneous action of these two mechanisms for regulating gene expression in the switch between dormancy and germination.

### 3.16. More on Transcription Factors

Many transcription factors (362) were differentially expressed in D vs. ND red rice seeds at 8 h of incubation at 30 °C (Supplemental Figure S16). Noteworthy, two whole classes (BINs) of transcription factors were differentially expressed (with a *p* < 0.01, according to the Wilcoxon Rank Sum Test and using the Benjamini and Hochberg correction, which were performed in MapMan to predict BINs that exhibited a different behavior in terms of expression profile compared to all the other remaining BINs; Supplemental Table S4): genes encoding for AS2 transcription factors were consistently, and sometimes strongly, overexpressed in ND seeds, whereas genes for MADS box transcription factors were consistently, and sometimes very strongly, overexpressed in D seeds (like the previously mentioned *OsMADS29*). Expression of these two families of transcription factors appears therefore to be quite specific to either germination (AS2) or dormancy (MADS box), in red rice.

Restricting the respiratory capacity seems to cause a down-regulation of growth and an up-regulation of many transcription factors associated with stress, as well as a down-regulation of those factors typically reported to be involved in growth, such as AS2 factors [162]. This latter, however, is evidently not the case in red rice ND seeds, wherein an impairment of the respiratory chain (apparent at the transcriptional level) is associated with over-expression of genes for AS2 transcription factors.

It has been suggested that 3 h may represent a specific switch point in the germination process of rice [20]. Specifically, Howell et al. [20] pointed out some germination-specific transcription factors encoding genes, with known homologs in arabidopsis, which showed transient expression at 3 h of imbibition: WUSCHEL-related homeobox (HB) transcription factors encoded at the loci Os08g0242400 (LOC_Os08g14400), Os03g0325600 (LOC_Os03g20910), and Os07g0684900 (LOC_Os07g48560), as well as a zinc finger homeodomain transcription factor encoded by Os09g0466400 (LOC_Os09g29130). In the present experiment, these four genes showed greater expression in ND than in D seeds (50.2-fold, 14.7-fold, 3.7-fold and 2.6-fold, respectively, at 30 °C 8 h; Supplemental Figure S19C), confirming their involvement in the germination process and showing that our 8 h timing is still reasonably comparable to the 3 h timing of Howell et al. [20] in terms of transcriptional regulation. This is probably owing to both timings being prior to the earliest time of germination of ND seeds at 30 °C.

Os01g0854500 (*OsWOX5* ~ *OsWOX9* and *QHB*), a rice WUSCHEL-type homeobox gene, which is likely involved in the specification and maintenance of the quiescent center of the root apical meristem [163], was much more expressed in ND seeds at 8 h of incubation at 30 °C (54.2-fold with respect to D seeds; Supplemental Figure S19D). *OsWOX5* expression is specifically induced by auxin and predominantly confined to radicle, indicating that it might be implicated in the development of radicle regulated by auxin [163]. Although as previously noted, imbibed red rice D seeds (at 8 h of incubation) showed increased expression of genes for auxin biosynthesis, a much stronger expression of *OsWOX5* in ND seeds would appear to support a role for this hormone even in seed germination, as reported for arabidopsis [23], confirming that important divergences in the transcription of auxin-related genes occur in D and ND seeds.

### 3.17. Gene Co-Expression Network Analysis and the Role of Photosynthesis-Related Transcripts

This analysis essentially allows to group together genes with close expression patterns [164]. It implicitly assumes that genes with a similar pattern of expression over a (not too small) number of conditions can prompt the existence of a common underpinning biological function.

**Figure 9:**
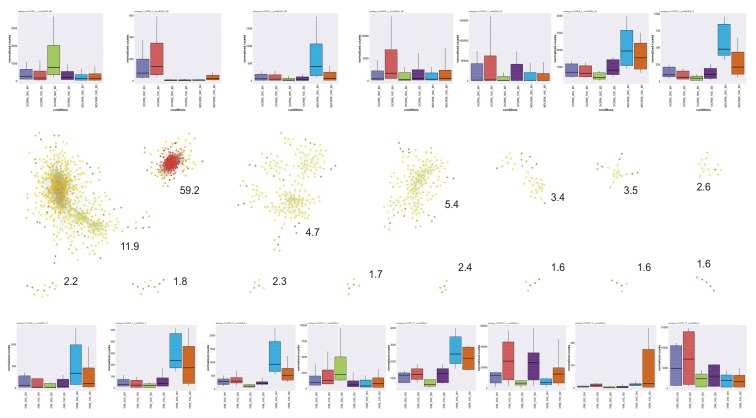
Co-expression network analysis: the clusters with at least five nodes (i.e., genes with correlated expression) are shown. Next to each cluster, its average node degree (i.e., average number of connections per node) is reported. Every node is colored according to its neighborhood connectivity (nc; that is, the average node degree of all the neighbors of that node), with green indicating a nc = 1 and increasing redness corresponding to an increasing nc value, with a maximum of 118.64. Next to every cluster, a box plot shows, for each tested condition, the median, the interquartile range, and the 15th and 85th percentiles (which are represented by the lower and upper whisker, respectively) of average expression level of the genes in the cluster.

At r = 0.96, the network was fragmented into several component clusters of gene expression (Figure 9 shows 15 clusters with at least five genes each; smaller clusters did not show any apparent interesting feature and then were not further considered), and some of them characterized ND seeds (see box plots in Figure 9). These clusters were analyzed for GO enrichment, and some of them were characterized by a significant and relevant enrichment in some GO terms (Supplemental Figures S20–S24). However, no one was associated with an expression pattern that could clearly characterize D seeds across all conditions. The largest cluster was associated with higher expression of many genes in D seeds after 8 d of incubation at 30 °C (Figure 9), including several genes involved in processes such as regulation of cell growth and nuclear transcription, or molecular functions linked to nucleotide binding, but defense response represented the most significantly enriched biological process (Supplemental Figure S20). Thus, it is not clear whether the higher expression of all these genes was mainly due to the adoption of diverse pre-emptive defense strategies in D and ND seeds, or some of them were really involved in seed dormancy. The second largest cluster was associated with higher expression of many genes in D seeds after 8 h of incubation at either 30 °C or 10 °C (Figure 9), and many genes linked to photosynthesis were included (Supplemental Figure S21). The third largest cluster corresponded to genes more expressed in ND seeds imbibed at 30 °C for 8 h (Figure 9), and it was mainly associated with oxidation-reduction processes and response to oxidative stress (Supplemental Figure S22A), as well as with activities localized to the extracellular region (Supplemental Figure S22B). The sixth cluster corresponded to genes more expressed in ND seeds incubated in water at 30 °C for 8 h or 10 °C for 8 d (Figure 9), and it was mainly associated with translation and ribosomal activity (Supplemental Figure S23). Finally, the 14th cluster corresponds to genes that were more expressed in ND seeds incubated in water at 10 °C for 8 d (Figure 9), and it was mainly associated with water transport (Supplemental Figure S24).

Although this analysis appears inconclusive in resolving a gene co-expression cluster specifically involved in seed dormancy across all conditions, network analysis showed that the second largest cluster has a higher average node degree (where the node degree, nd, is the number of directly connected neighbors) than the other clusters, and a much stronger neighborhood connectivity (nc, i.e., the average connectivity, or node degree, of all the immediate neighbors of each node) with respect to all the other clusters (Figure 9). Indeed, this cluster differentiates from the rest of the network also by other topological features (see Supplemental file “Insight into gene co-expression network analysis”) indicating that the expression of these genes is highly inter-connected and strongly suggesting this cluster represents a functional module associated with a structural unit, which turns out to be the plastid, since the clustered genes are essentially related to photosynthesis and chloroplast metabolism (Supplemental Figure S21). Indeed, genes encoding for photosynthesis-related proteins must be coordinately expressed, and most proteins involved in photosynthesis are encoded in the nucleus and are imported into the plastid [122].

Actually, in seeds there are only non-green plastids, presumably proplastids, which in rice display a limited thylakoid system and contain cytoplasmic tubular and vesicular inclusions formed by invaginations of the outer plastid membranes [165,166]. Anyway, the dry proplastid must restore its functionality following imbibition. Thus, a role of this cluster in plastid function may explain why its secondary topological features differ from those of the other clusters (see Supplemental file “Insight into gene co-expression network analysis”). The higher expression (Supplemental Figure S15E), in imbibed D seeds (8 h), of a gene for β-tubulin (*OsTUB8*; showing a 66.7-fold higher expression with respect to ND seeds, at 30 °C) and of gene Os02g0729400 encoding for a rhodanese-like domain containing protein that seems to act as extracellular calcium sensing receptor involved in the organization of plastids [167] (with no detectable expression in ND seeds, at 30 °C), as well as the high connectivity they have within cluster 2, would confirm that such cluster can be linked to cell organization for plastid functionality. In accordance, another gene that is included in the second cluster is *OsMADS29*, which, as previously seen, can stimulate the differentiation of the proplastid to amyloplast. Even the previously mentioned locus *ASR5*, encoding for a protein that is present in multiple cellular compartments, including the plastid, and may regulate genes related to photosynthesis [168], belongs to this cluster.

Even though seed dormancy was not associated with a specific gene co-expression network that holds at both early (8 h of imbibition) and stabilized (8 d) incubation time, the high inter-connectivity of plastid-related genes expressed early (8 h) in D seeds (Figure 9; second cluster) is noteworthy. In arabidopsis, temporal transcriptome profiling showed that the expression of genes for organelle biogenesis is an essential developmental step for germination [145]. Although, in rice germination, the peak in transcript abundance for components encoding the machinery of oxidative phosphorylation for energy metabolism is 24 h after the start of imbibition [169], Narsai et al. [145] showed that the presence of mitochondrial DNA replication factors and RNA-processing functions in the transcriptome profile represents the earliest events in the expression of germination-specific genes, preceding any changes associated with energy metabolism, and occurring even before 8 h of imbibition. This was consistent with some other observations made in rice, which revealed a surge in transcript abundance for genes encoding transport functions at 3 h of imbibition [20], suggesting that signals (and responses) affecting mitochondrial function are taking place earlier in germination in rice as well [145]. However, the absence of relevant co-expression clusters for the mitochondrion functionality, suggests that this organelle has no specific role in maintaining dormancy. Surely, mitochondrial biogenesis plays a crucial role from the very early stages of seed germination [20], but imbibed dormant red rice seeds need energy metabolism as well. Although some genes for mitochondrion functionality (mitochondrial import inner membrane translocase subunits Os03g0114900 and Os07g0604500) were more expressed in ND seeds (3-fold and 2.3-fold with respect to D seeds, respectively, at 30 °C 8 h; Supplemental Figure S15F), neither they showed the same high degree of differential expression as for the chloroplast, nor they formed a co-expression cluster under our experimental conditions.

Although it might be wondered whether the second cluster is due to transcripts remaining from the developing green seed, large changes in abundance of many transcripts already occur as early as within 3 h of imbibition [20]. In fact, 3 h may represent a specific switch point in the germination process [20], indicating that the state of the transcriptome at 8 h is significant from a physiological point of view. Analogously, in arabidopsis, the transcriptome of imbibed seeds is widely reprogrammed within 6 h after the onset of imbibition [138] and the changes in the number of transcripts, including degradation of transcripts that had accumulated during seed maturation, may commence directly upon imbibition and are highly abundant during the first 6 h of imbibition [19]. Furthermore, developing rice caryopses are green, up to the inception of physiological maturity, because of some chlorophyll persisting in the pericarp [170], which in the mature grain is a dead covering tissue that ought not convey any remnant transcript to the imbibed D seed.

Correspondingly, dry seeds contain many nuclear transcripts encoding chloroplast proteins, including several that are involved in photosynthesis, but a large number of transcripts for plastid proteins show only a very low expression in dry ND seeds that rises quickly during imbibition [20,145,161]. In spinach, though the nuclear genes encoding plastid ribosomal proteins are expressed very early during seed imbibition, photosynthetic genes and plastid-encoded genes are highly expressed only late in chloroplast development during seedling growth [171].

Hence, even though the association between the chloroplast-related expression cluster and the dormancy status of imbibing seeds does not definitively prove that the functionality of the chloroplast (or, better, some functions of the proplastid) is necessary to maintain dormancy, it points out that this could indeed be the case. In fact, on the one hand, the size and the strong interconnectivity of the second cluster, found after the timepoint for the germination switch, demonstrate that these transcripts are not just casual leftovers from the grain filling stage, but they represent a functional module that is prominently conserved in the D seed. On the other hand, there was not an analogously strong differentiation for genes linked to the functionality of the mitochondrion, which is evidently essential for seeds in every condition [145,172]. The gene cluster linked to the functionality of the mitochondrion is therefore not revealed in the co-expression network as obtained from our study, which is mainly based on the picking of differences between D and ND seeds.

The fact that, in the present study, even ND seeds, after 8 d of incubation at 10 °C, provided to rebuild transcripts for photosynthesis-related genes (Supplemental Figure S7), shows that chloroplast (or proplastid) functionality lately becomes important in these seeds as well, probably in view of the development of the seedling, even though germination has not yet started, at least visibly. This is in agreement with findings of Bassel et al. [173], who argued that the up-regulation of photosynthetic machinery in arabidopsis seeds may be a reflection of the seed commitment to germinate in anticipation of autotrophic growth, at least after 24 h of incubation at optimal temperature. Indeed, even in barley, genes for photosynthesis and the chloroplast protein synthesis machinery are specifically and coordinately up-regulated in the post-germination phase, even in the dark, to support the transition to photo-autotrophic growth [14]. Correspondingly, expression of genes for photosynthesis, including those related to light reaction, photorespiration, and the Calvin Cycle, was activated only between 36 and 46 h of incubation in water, in both barley [72] and wheat [56] germinating seeds. In germinating soybean embryonic axes, a remarkable enrichment in photosynthesis genes was present at 24 h of incubation in water, in preparation for autotrophic seedling growth [77]. The earlier preparation to seedling establishment in this species might be due to the higher optimal temperature for germination, closer to that used here for red rice. Present findings show that, in red rice, up-regulation of photosynthetic machinery genes in anticipation of autotrophic growth could be observed in ND seeds even at 8 d of incubation (at 10 °C), but, oddly, it was much more intense in D seeds at an earlier time (8 h). 

It can, thus, be wondered why early chloroplast functionality (actually some chloroplast-like function of the proplastid) is so strongly guaranteed, at least at the transcriptome level, in D seeds, which are developmentally blocked and therefore do not need to activate photosynthesis, at least in the immediacy. In fact, no greening of embryonic tissues occurs in these seeds, even if they are exposed to light [6]. It can be inferred that some chloroplastic functions of the non-green proplastid are actually firmly promoted in these seeds. As said, this evident teleonomy strongly prompts some role of the proplastid in seed dormancy.

One role is certainly played in the synthesis of red pigments, to protect the caryopsis (Figure 3). In fact, several genes for the synthesis of PAs (Figure 4) belong to the highly-interconnected second cluster, and the Rc gene, activating PA synthesis, is central to this cluster, with one of the highest node degree, i.e., 134. Indeed, this gene is a master regulator of the PA biosynthesis pathway [174,175], and has been suggested to pleiotropically control both dormancy and pigment traits by regulating ABA and flavonoid biosynthetic pathways, respectively [83]. Moreover, a direct role of the proplastid in maintaining seed dormancy can occur through carotenoid biosynthesis, which starts in the chloroplast (in the proplastid, in the case of seeds), is needing to maintain dormancy [81], and eventually leads to ABA.

### 3.18. Long Non-Coding RNAs

Three DEGs belonging to the second co-expression cluster (Figure 9) were identified as lncRNAs: Os02g0653000 (CantataDB codes CNT0030133 and CNT0030134), Os01g0800701 (CNT0032682) and Os06g0132450 (CNT0028870). A role in the regulation of plastid functionality might therefore be hypothesized for these lncRNAs.

## 4. Materials and Methods

### 4.1. Seed Materials and Experimental Setup

A straw-hulled red rice originally found in a paddy close to Vercelli (located in a rice-growing area of the Po Valley, Italy), and previously used for other studies [6,81,91], was grown in a greenhouse at Fiorenzuola d’Arda (Italy). The seed was harvested when showing shattering capability and dried for 1 d at 35 °C [6]. Dormant red rice spikelets (<1% germination [6]) were stored at −15 °C till use. Fully germinating (ND, >99% germination) seeds were obtained by dry-afterripening dormant spikelets at 30 °C for 16 weeks [9]. Naked (dehulled) caryopses were prepared by manually dehulling the spikelets prior to the start of the experiment [6,13]. Dehulled red rice caryopses were incubated in water in Petri dishes enclosed in a humidity box. Dormant caryopses were incubated either at 30 °C or 10 °C for either 8 h or 8 d. For comparison, fully afterripened (ND) caryopses were incubated for either 8 h at 30 °C or 8 d at 10 °C.

For each treatment, three replicated dishes were each prepared by placing 15 caryopses on two filter paper discs with 5 mL of water. At every sampling time, for each replicate, all 15 imbibed seeds (approximately 450 mg) were immediately frozen in liquid nitrogen and stored at −80 °C. Samples were subsequently ground in liquid nitrogen and further stored at −80 °C. Additional germination tests were performed as above but with 20 caryopses per dish to assay germinative capability.

### 4.2. RNA Extraction, Libraries Preparation and Sequencing

Total RNA was extracted by a protocol modified after López-Gómez and Gómez-Lim [176]. Briefly, 15 mL tubes with samples were kept in liquid nitrogen and singularly transferred to ice; 1.4 mL of cold ethanol containing 6.8% β-mercaptoethanol was added to the tube that was turned upside down and hit against the counter a few times, to suspend the frozen powdered pellet. The tube was vortexed till the sample was fully suspended in the ethanol/β-mercaptoethanol mixture. One mL of TE-saturated phenol (pH 8) was added to the single tube, and after quick vortexing, 1 mL of 24:1 chloroform/isopropanol was added, and the tube was shortly vortexed once more. Five mL of extraction buffer (150 mM Tris/borate pH 8 containing 50 mM EDTA and 2% SDS) was added, the tube was vortexed 1′, and left on ice. Then, 0.6 mL of 5 M potassium acetate was added to each sample, which was turned upside down ten times and left on ice for 1 h. Tubes were centrifuged 5′ at 15,000 g and 5 mL of the upper phase of each sample was transferred to a new 15 mL tube. Five mL of 6 M LiCl was added and the tubes were gently turned upside down several times and left 30′ on ice. They were centrifuged 10′ at 20,000 g and the upper phase was then poured away; open tubes were left to drain upside down for >1′. Pellets were washed with 2 mL 80% ethanol, and tubes were centrifuged 5′ at 15,000 g. The liquid was carefully removed, and the tubes left 10′ upside down. Pellets were re-suspended in 700 µL DEPC-treated water by heating and each sample was transferred to a 2 mL tube. Thirty-three µL of 5 M NaCl and 1.3 mL cold ethanol were added; tubes were turned upside down a few times and left 30′ on ice. They were centrifuged 12′ at 20,000 g and the liquid was carefully removed. Pellets were washed with 0.2 mL 80% ethanol, and tubes were centrifuged 5′ at 20,000 g. Any liquid was carefully removed, and open tubes were left (upside down) to dry 5′ at 37 °C. Pellets were dissolved in 100 µL DEPC-treated water by heating. DNA was removed by treating 20′ with RNase-free DNAse. RNA integrity number (RIN) was determined with a 2100 Bioanalyzer (Agilent) using the Agilent RNA 6000 Nano Kit and following provided instructions.

RNA-Seq libraries were prepared with the TruSeq RNA sample preparation kit (Illumina), according to manufacturer’s instructions. One µg of total RNA was utilized for each sample. This protocol involves removal of rRNA. The quality of libraries was checked with a 2100 Bioanalyzer (Agilent) using the Agilent DNA 1000 Kit and following provided instructions. Libraries were quantified through qRT-PCR, as recommended by the manufacturer’s instructions, and single-end sequenced for 51 bases on an Illumina Genome Analyzer (GAIIx).

Raw sequencing reads are available in the ArrayExpress database (http://www.ebi.ac.uk/arrayexpress) under accession number E-MTAB-6740.

### 4.3. Bioinformatic and Statistical Methods

Raw fastQ files were checked for low-quality reads and contaminants via fastQC application (version 11.1, downloaded from https://www.bioinformatics.babraham.ac.uk/projects/fastqc/). Reads (51 nt) containing contaminant primer/adapters and long stretches of poor quality bases were trimmed out with the Cutadapt software [177]. Contaminant-free, filtered reads were mapped with Bowtie2-2.2.5 [178] and Tophat2 version 2.0.14 [179] to the rice genome (*O. sativa* Nipponbare IRGSP-1.0.27 release). Based on rice small intron size, minimum and maximum intron length of 30 and 50,000 bp, respectively, were set. Read counts were collected from the BAM alignment files with HTSeq version 0.6.1p1 in the single-end and ‘union’ mode using the *O. sativa* IRGSP-1.0.27 gtf file as obtained from the Ensembl Plants Repository. Spearman correlation coefficients among biological replicates were always greater than 0.90.

Reads mapping against genomes of several *Oryza* species was conducted with Bowtie2 and Tophat2 as described above for *O. sativa* Nipponbare. Genome sequences and corresponding GTF files for all rice species were obtained from Ensembl Plants repository (http://plants.ensembl.org/info/website/ftp/index.html).

### 4.4. DEG Calling

The Bioconductor DESeq2 package [180] version 1.8 was implemented to call differentially expressed genes (DEGs) using parametric fit and betaPrior parameter set to False. Thresholds for FDR (Benjamini-Hochberg false discovery rate) and fold change (FC) were set to 0.001 and 2, respectively. Expression level values presented in the paper are DESeq2 counts normalized across all samples. The expression level of every treatment was evaluated on three biological repeats, each obtained as a bulk of 15 caryopses (that is, each repeat corresponded to the seed bulk from one of the three replicated Petri dishes).

### 4.5. Screening DEGs for Biological Functions

MapMan [27,28] figures were generated by importing DESeq2-normalized expression data for DEGs in MapMan application [181]. Binning of DEG sequences to MapMan “BINs” (which represent functional classes of genes) was accomplished by using the Osa_RAPDB_mapping file (RAPDB-IRGSP1.0; downloaded from http://mapman.gabipd.org/web/guest/mapmanstore).

The following databases were used to characterize relevant genes: NBRP-Rice Oryzabase (https://shigen.nig.ac.jp/rice/oryzabase/), funRiceGenes (https://funricegenes.github.io), NCBI-Gene (https://www.ncbi.nlm.nih.gov/gene), MSU Rice Genome Annotation Project (http://rice.plantbiology.msu.edu/index.shtml), CoP (http://webs2.kazusa.or.jp/kagiana/cop0911/), RAP-DB (http://rapdb.dna.affrc.go.jp/), Ensembl Plants (http://plants.ensembl.org/index.html), KEGG (http://www.genome.jp/kegg/), UniProt (http://www.uniprot.org/).

### 4.6. GO Term Enrichment Analyses

GO (Gene Ontology) terms associated to genes were obtained with BiomaRt queries (genome release *Oryza sativa* IRGSP-1.0.27). To account for RNA length bias typical of RNA-Seq approaches, the goseq bioconductor package was employed. Gene lengths were retrieved with BiomaRt queries (*Oryza sativa* IRGSP-1.0.27) based on cDNA and median length for each rice locus used. An FDR cutoff of 0.05 was used for GO enrichments.

### 4.7. Co-Expression Analyses

A matrix of 10,336 rows (genes called as DEG; FDR in at least one of the contrasts) and as columns all 18 samples (three replicates for each of the six tested conditions) was generated by subsampling the whole DESeq2-normalized expression data matrix (DESeq2 countSet). For such matrix, the adjacency function as available in R WGCNA package [182] was implemented for calculation of signed network adjacency. Correlation threshold was set to 0.96. To obtain edges and nodes, a graphNEL-type graph was subsequently generated from the adjacency matrix and sent via the Rcytoscape Bioconductor package [183] to Cytoscape application version 2.8.1 [184] for cluster visualization and analysis. Communication from and to Cytoscape from R for batch analyses was ensured by functions from R Cytoscape package. Biological Process enriched GOs for genes in cluster were calculated with the hypergeometric test as implemented in Bioconductor GOstats package [185] using a *p*-value cutoff of 0.01.

### 4.8. Quantitative RT-PCR Analysis

The validation of expression patterns of representative genes obtained by RNA-Seq analysis was performed by quantitative PCR (qPCR) analysis. Two-step RT-qPCRs were carried out using the same RNAs utilized for the RNA-Seq experiment. Two technical replicates for each biological replicate were performed. cDNAs were synthesized by the Super Script II enzyme (Invitrogen) following manufacturer’s instructions and quantified through the Qubit Fluorometer (Invitrogen). qPCRs reactions were carried out using the KAPA SYBR FAST ABI Prism qPCR Kit (ResnovA) according to manufacturer’s instructions, by means of a 7300 Real Time PCR System (Applied Biosystems, Foster City, CA, USA). Primers were designed with the NCBI Primer-BLAST software (https://www.ncbi.nlm.nih.gov/tools/primer-blast/) which directly checks the specificity of each primer for the corresponding gene. The sequences of primers are listed in Supplemental Table S5. The *Edf* gene (LOC_Os08g27850 ~ Os08g0366100) was used as internal control [186]. The specificity of the reactions was verified by melting curve analysis. Relative gene expression was calculated with the 2^−ΔΔCT^ method [187]. The relative mRNA level averages across the different tested conditions were normalized to the highest average value.

## 5. Conclusions

The present study has provided several clues on the regulation of dormancy and germination in red rice; in fact, gene expression data suggest that: (i) long dry-afterripening imposes a strong respiratory impairment onto ND seeds; (ii) in accordance, following imbibition, glycolysis is preferentially directed to alcoholic fermentation in ND seeds but to alanine production in D ones; (iii) PEPCK, pyruvate phosphate dikinase and alanine aminotransferase pathways have an important gluconeogenetic role associated with the restoration of plastid functions in the D seed early during imbibition; (iv) correspondingly, co-expression analysis pointed out a strong commitment to guarantee plastid functionality in D seeds; (v) altogether, putative reconstruction of general metabolism prompts a preferred usage of carbon and nitrogen resources to biosynthetic processes in the plastid in D seeds during imbibition, including starch and PAs accumulation; (vi) the pathway to PA synthesis is a process activated in D seeds at least at the transcription level, and it is apparently involved in a specific defense strategy that differs from that of ND seeds; (vii) among phytohormones, JAs, auxin, ABA and GA showed an involvement, but there was no evidence for a preeminent role of ABA in seed dormancy; (viii) once their metabolism was stabilized (8 d), D seeds showed a higher expression of some regulative genes related to the control of growth, such as *OsTOR*; (ix) chromatin modifications appeared to be involved in actuating the transition from dormancy to germination; (x) ND seeds showed a higher expression of several genes related to cell wall modification, such as genes encoding for expansins OsEXPA2, OsEXPA4 and OsEXPB6, xyloglucan endotransglycosylase OsXTR1 (~XTH2) and pectin methylesterase OsPME2, consistent with analogous findings for orthologous genes across several species, and suggesting they prepared for acrospire/radicle elongation.

## Figures and Tables

**Table 1:** Germination tests (averages ± se). Germination recorded as pericarp splitting (ps, first visible stage of germination) and as seedling growth stage S1 (S1, rootlet or coleoptile ≥1 mm). Averages obtained for the seeds used in the RNA-Seq experiment (3 replicates of 15 seeds, 45 seeds total) are evidenced in bold, the other averages were obtained in additional tests (5 replicates of 20 seeds, 100 seeds total).

Test	D		ND	
	ps	S1	ps	S1
	(%)	(%)	(%)	(%)
**8 h 30 °C**	**0 ± 0**	**0 ± 0**	**0 ± 0**	**0 ± 0**
**8 d 30 °C**	**0 ± 0**	**0 ± 0**	99 ± 1	99 ± 1
14 d 30 °C	1 ± 1	1 ± 1	100 ± 0	100 ± 0
**8 h 10 °C**	**0 ± 0**	**0 ± 0**	0 ± 0	0 ± 0
8 h 10 °C + 14 d 30 °C	2 ± 1	2 ± 1	100 ± 0	100 ± 0
**8 d 10 °C**	**0 ± 0**	**0 ± 0**	**0 ± 0**	**0 ± 0**
8 d 10 °C + 14 d 30 °C	7 ± 5	7 ± 5	100 ± 0	100 ± 0

**Table 2:** Number of expressed sequences detected in imbibed caryopses.

Seed	Incubation Temperature (°C)	Time of Incubation	Total Number of Transcripts	Number of Non-Coding Transcripts
Dormant	30	8 h	32,355	3381
		8 days	28,865	2469
	10	8 h	30,378	2418
		8 days	31,747	3214
Non-Dormant	30	8 h	31,967	3116
	10	8 days	31,968	2899

**Table 3:** Number of differentially expressed genes (DEGs) detected in imbibed caryopses.

Comparison	DEGs	DEGs for Non-Coding Transcripts	Intent of the Comparison (Highlighted Differences)
D 30 °C 8 h vs. ND 30 °C 8 h	3772	18	Transition to germination during imbibition
D 10 °C 8 d vs. ND 10 °C 8 d	92	0	Transition to (potential) germination when metabolism has stabilized
D 30 °C 8 d vs. D 30 °C 8 h	4468	24	Stabilization of metabolism in D seeds at normal temperature
D 30 °C 8 d vs. D 10 °C 8 d	5131	36	Assessment of temperature effect in D seeds (stabilized metabolism)
D 30 °C 8 h vs. D 10 °C 8 h	1299	4	Assessment of temperature effect in D seeds (during imbibition)
D 10 °C 8 d vs. D 10 °C 8 h	3192	9	Stabilization of metabolism in D seeds at low temperature

## Supplementary Materials

The following are available online at http://www.mdpi.com/2223-7747/7/2/35/s1, Supplemental Tables S1–S5 (Supplemental Tables.pdf), Supplemental Figures S1–S24 (Supplemental Figures.pdf), and Supplemental files “100 most abundant.xlsx” and “Expression_data_for_all_genes_in_all_conditions.xlsx” are provided. Six Insights are also available: Insight into variability between replicates, Insight into the hypoxic-like stress caused by dry-afterripening, Insight into the carbon metabolism, Insight into mRNA levels of seed storage proteins, Insight into pre-emptive defense strategies, Insight into gene co-expression network analysis.

## Abbreviations

D	dormant
ND	non-dormant
DEG	differentially expressed gene
FC	fold change
GO	Gene Ontology
PAs	proanthocyanidins
JA	jasmonate
GA	gibberellin
ABA	abscisic acid
