# Supplementary material for: Seed Dormancy Involves a Transcriptional Program That Supports Early Plastid Functionality during Imbibition
Source: Plants (Basel). 2018 Apr 19;7(2):35. doi: 10.3390/plants7020035 (PMC6026906; doi:10.3390/plants7020035)
Supplement: Supplementary file 1 [file plants-07-00035-s001.zip › plants-273866-supplementary-update/Insight into gene co-expression network analysis.pdf]

Supplementary material to the article "Seed dormancy involves a transcriptional program that supports early plastid functionality during imbibition" by Gianinetti et al.

Insight into gene co-expression network analysis

Analysis of the co-expression network (by the Network-Analyzer plugin of Cytoscape and considering the network as undirected) obtained for r = 0.96, showed that the overall network basically would seem to have a scale-free topology, as suggested by the initially linear trend of the node degree distribution on a double logarithmic scale (Fig. In1A of this Insight). A scale-free topology means that the number of nodes with a given node degree (i.e., number of connections) is inversely related to the node degree raised to some constant power, and the degree distribution follows the same power law independently of the scale of observation and the size of the network. Plotting the cumulative distribution of node degree, which smoothes the plot and eliminates the plateau observed at high node degrees (Barabási, 2016), confirms that the network is compatible with a scale-free topology, but shows a high-degree cutoff (Fig. In1B of this Insight).

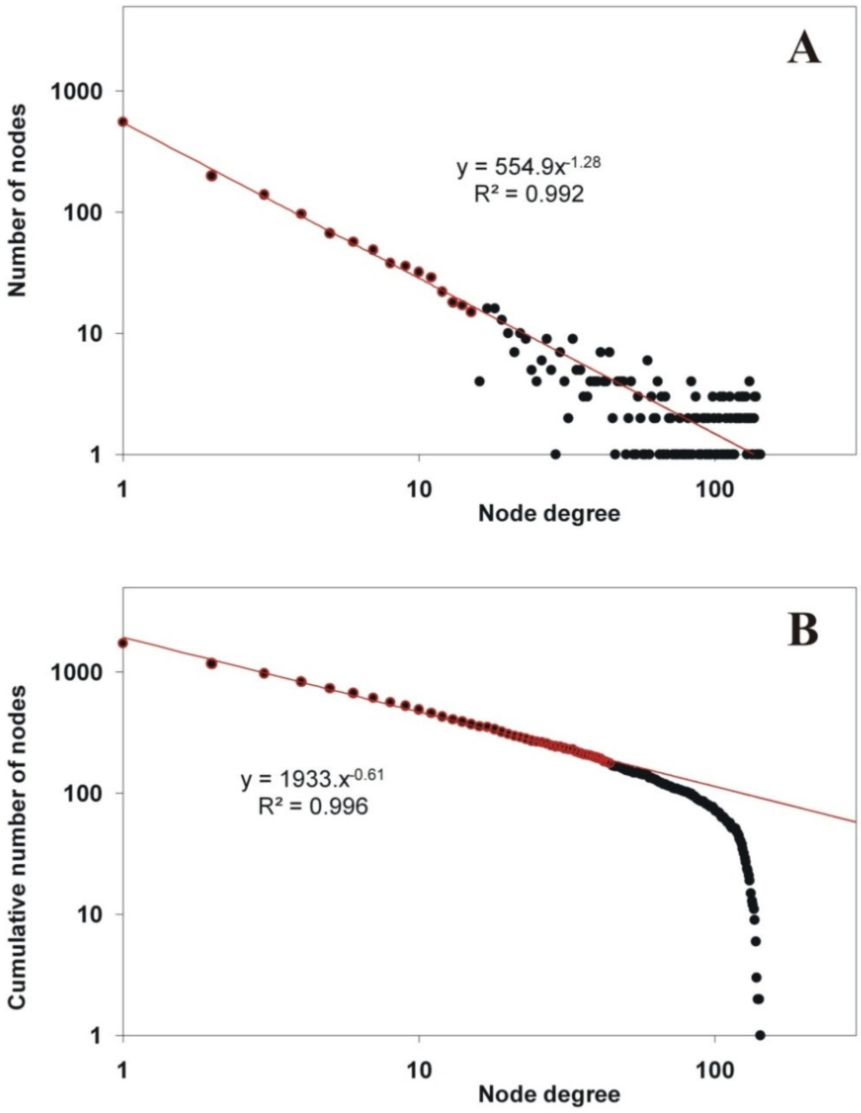

Figure In1. (A) Node degree distribution of the co-expression network (built with r = 0.96). Since this distribution is linear (at least, initially) on a log-log scale, the network appears compatible with a scale-free topology. (B) Cumulative node degree distribution (i.e., the number of nodes with a degree equal or greater than that indicated on the x-axis is plotted) of the co-expression network: a clear scale-free region, more extended than in the previous plot, is apparent before a high-degree cutoff. In both plots, the red line is the regression based on the log-log linear (apparently, scale-free) region, whose datapoints are highlighted by a red border.

A high-degree cutoff is a rapid drop, above some given value, in the proportion of nodes having a node degree above that value with respect to what expected in a pure power law (Barabási, 2016). Regressions based on the log-log linear (apparently, scale-free) region, where a pure power law holds (Fig. In1 of this Insight), suggest the absolute value of the decay exponent of the node degree distribution (γ, the degree exponent) is approximately 1.61 in the scale-free region: direct estimation is 1.28, but the cumulative distribution provides a better, even though still approximate, estimate, which, however, has to be incremented by one (Barabási, 2016).

Scale-free networks with $\gamma < 2$ show several anomalous features (Barabási, 2016). Actually, no large network can exist with $\gamma < 2$ because for a sufficiently large number of nodes the largest node degree must exceed the total number of nodes in the network, and thus it will run out of nodes to connect to (Barabási, 2016). So, it shows a rapid drop in the node degree distribution at high degrees (the high-degree cutoff). On the other hand, as the smaller is γ, the shorter are the distances between the nodes (Barabási, 2016), this gene co-expression network appears to favour the reduction of paths (that is, the number of links that, on average, separate two nodes). This is probably due to the fact that gene expression is co-regulated within what can be roughly considered as functional modules with different metabolic functions, which are integrated but not strictly correlated between them. This functional compartmentation can be expected to cause a much higher correlation of expression among genes belonging to the same metabolic function, but a poor correlation between genes of different functional modules, posing a limit to node connectivity that is well below the extension of the whole network. This would explain why this network can work even with $\gamma < 2$.

When, at $r = 0.96$, the network was fragmented into several component clusters of gene expression (Fig. 9), it turned out that the second largest cluster had a higher average node degree (nd; that is, the number of edges; for an undirected network it corresponds to the number of directly connected neighbours) and a much stronger neighbourhood connectivity (nc; i.e., the average connectivity, or node degree, of all the immediate neighbours of each node) than all the other clusters (Fig. 9). Indeed, this cluster differentiates from the rest of the network by a specific characteristic: as the node degree increases, the distribution of topological coefficients (i.e., the tendency of the nodes in the network to have shared neighbours) decreases according to a power law in the rest of the network, but it decreases linearly, and almost nothing, for this cluster (Fig. In2 of this Insight).

A linear decrease of topological coefficients indicates that, in the second cluster, the proportion of nodes with a high degree that also have a high number of shared neighbours is unusually high. In other words, the expression of the genes belonging to this cluster is highly inter-connected. This is consistent with an even stronger reduction of paths and, then, a closer co-regulation.

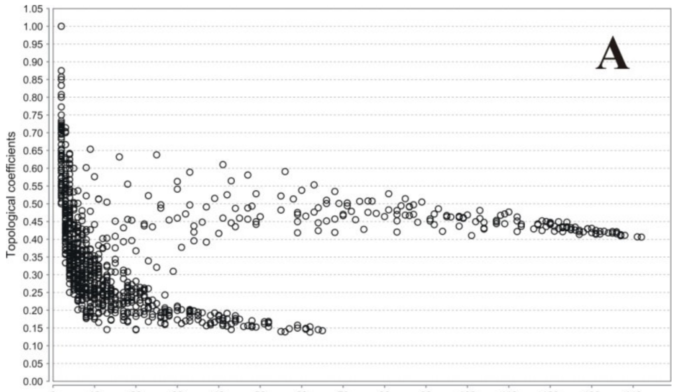

Figure In2. Distribution of topological coefficients (i.e., the tendency of the nodes in the network to have shared neighbours) as a function of the number of neighbours (i.e., node degree) for: (A) the whole co-expression network, (B) the second cluster, (C) all the network apart from the second cluster.

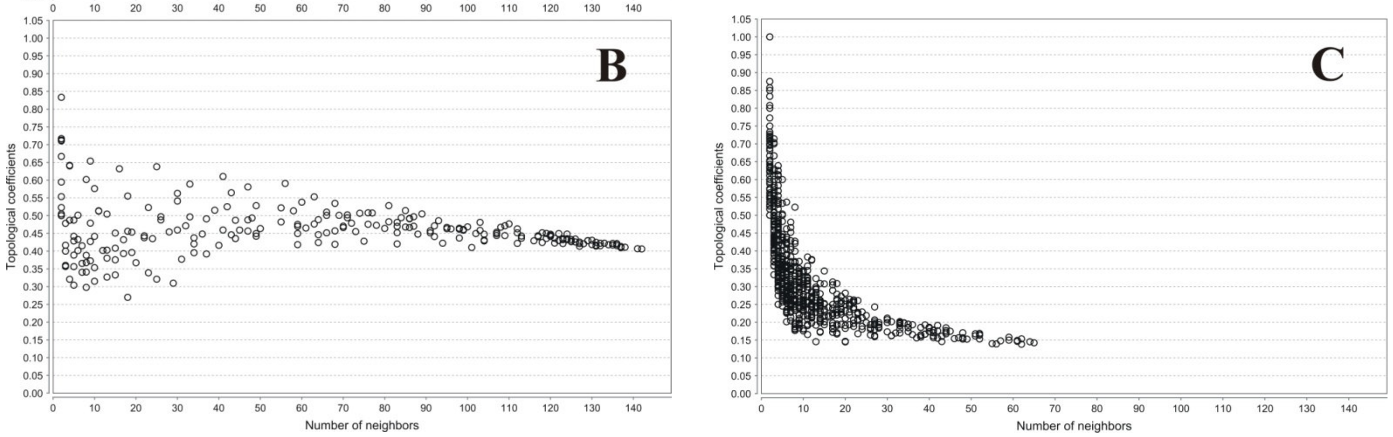

Notably, the gene expression coherence is often related to functional coherence (Bhattacharya and Cui, 2017). Although possibly unknown, such a common function binds together the expression of involved genes, acting as a hidden linkage factor. Thus, this analysis can hint to the common function, if this is hidden, and/or it can highlight which genes are involved in its accomplishment. At least two further assumptions are required to these purposes: (a) that the similar pattern of expression is causal and not a random effect, and (b) transcriptional modulation is an important, even though not unique, mode of physiological regulation. In this sense, the overall features of a network of genes having corresponding expression patterns, can evidence some collective properties (i.e., a network topology) that are not apparent when considering single genes. When a large number of genes is involved in determining a given co-expression topology, a random effect is poorly probable, and a transcriptional modulation seems probable.

It ought to be noted that link removal, consequent to adopting a minimum threshold of correlation to construct a network, does not appear to be responsible of the cutoff, since the node degree distribution of the network built with a lower correlation, $r = 0.90$, shows a cutoff (Fig. In3A of this Insight) like that built with $r = 0.96$ (Fig. In1B of this Insight), notwithstanding a much higher number of connections (the whole number of links, or edges, passes from 12,814 with $r = 0.96$ to 105,963 with $r = 0.90$). As γ further decreases when the minimum threshold of correlation passes from 0.96 to 0.90, it can be inferred that the observed topology is not merely due to choice of the minimum threshold of correlation. Neither the maximum number of links for node in each cluster is simply constrained to the size of the cluster. In fact, the node degree distribution of the largest cluster shows a cutoff at a node degree lower than that of the second cluster (Fig. In3B-C of this Insight).

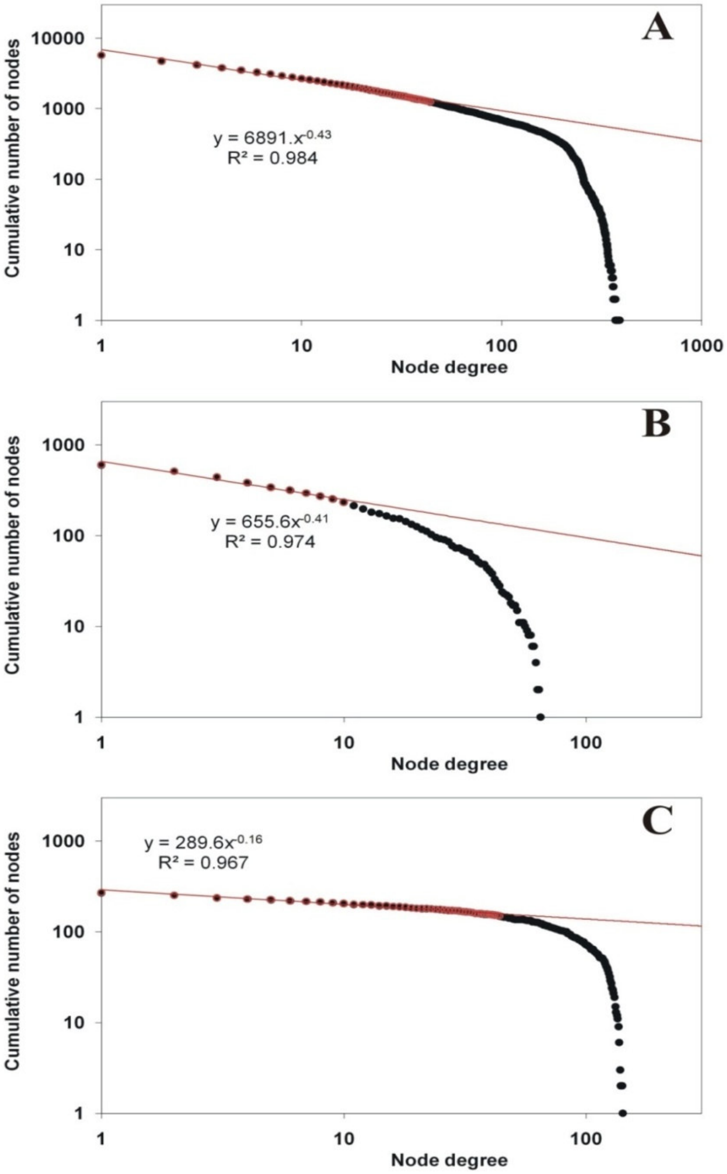

Figure In3. Cumulative node degree distribution (i.e., the number of nodes with degree equal or greater than that indicated on the x-axis is plotted) of: (A) the co-expression network built with r = 0.90; (B) the largest cluster of the chosen co-expression network (the one built with r = 0.96); (C) the second largest cluster of the chosen co-expression network (the one built with r = 0.96). In every case, a clear scale-free region is apparent before a high-degree cutoff. In all plots, the red line is the regression based on the log-log linear (apparently, scale-free) region, whose datapoints are highlighted by a red border. These regressions suggest the absolute value of the decay exponent of the node degree distribution (γ, the degree exponent) is < 2 (the estimate obtained from a cumulative distribution has to be incremented by one; Barabási, 2016) even if a lower r is adopted to build up the network.

The unusually uniform distribution of node degrees (that is, the low degree exponent, γ) of the expression network can be due to the necessity of a close correlation between the expression of genes cooperating into the same function, or into cooperating functions. Hence, the high-degree cutoff could be due to the constraining of the largest node degree to the number of nodes, i.e. genes, which directly integrate their activities within functional modules, thus that they have quite uniform connectivity with reduced paths of correlation (that is, they show close co-expression) and also display functionally limited maximum node degree. Low γ (that is, quite uniform connectivity) and sharp high-degree-cutoff (truncation of connectivity outside the functional module) are maximally evident for the second cluster (Fig. In3C of this Insight), which is associated with plastid functionality (Supplementary Fig. S21). Thus, a probable reason for the peculiar features of the second cluster is that it pertains to a structural unit, the plastid. This would explain both the high inter-connectivity among its members and the drop of connectivity with genes outside the unit.

References

Barabási A.-L. (2016). Network Science. Cambridge University Press.

Bhattacharya A. and Cui Y. (2017). A GPU-accelerated algorithm for biclustering analysis and detection of condition-dependent coexpression network modules. Sci. Rep. 7:1-9.
