# Supplementary material for: Seed Dormancy Involves a Transcriptional Program That Supports Early Plastid Functionality during Imbibition
Source: Plants (Basel). 2018 Apr 19;7(2):35. doi: 10.3390/plants7020035 (PMC6026906; doi:10.3390/plants7020035)
Supplement: Supplementary file 1 [file plants-07-00035-s001.zip › plants-273866-supplementary-update/Insight into mRNA levels of seed storage proteins.pdf]

Supplementary material to the article "Seed dormancy involves a transcriptional program that supports early plastid functionality during imbibition" by Gianinetti et al.

Insight into mRNA levels of seed storage proteins

Although seed storage proteins are mainly accumulated during seed maturation and are typically described as a carbon and nitrogen sources for seed germination and seedling establishment, they can be synthesized during imbibition as well (Galland et al., 2014). This translational activity associated with the resumption of the late maturation translational program has been interpreted as reflecting the maintenance of an embryonic maturation program for the translatome during early imbibition as an adaptive transition from a quiescent to a highly active metabolic state (Galland et al., 2014). However, overall *de novo* protein synthesis is low during the first 8 h of imbibition (Galland et al., 2014), thus that no substantial additional synthesis of seed storage proteins is supposed to occur. Actually, the main nitrogen source for novel synthesis in the imbibed seed is represented by degradation of seed storage proteins, thus that novel synthesis of seed storage proteins would seem to be a futile cycle. Seed storage proteins predominantly accumulate in the endosperm, and their genes are expressed in that region (Qu and Takaiwa, 2004). In dry seeds, these transcripts remain as highly expressed long-lived mRNAs species (An and Lin, 2011; Rajjou et al., 2012). Hence, mRNA abundance for seed storage proteins does not necessarily result in a change in the corresponding protein abundance, because the large amount of these specific mRNAs in red rice seeds is not associated with translational exigencies. Indeed, selective mRNA translation is a key feature of seed germination (Galland et al., 2014).

In this context, it is quite singular that two (almost identical) genes for glutelin type B1 proteins, *GluB-1a* and *GluB-1b*, were very much more expressed ($log_2FC \geq 5$; Fig. In1 of this Insight) in dormant than in nondormant seeds both at 8 h (30 °C) and at 8 d (10 °C).

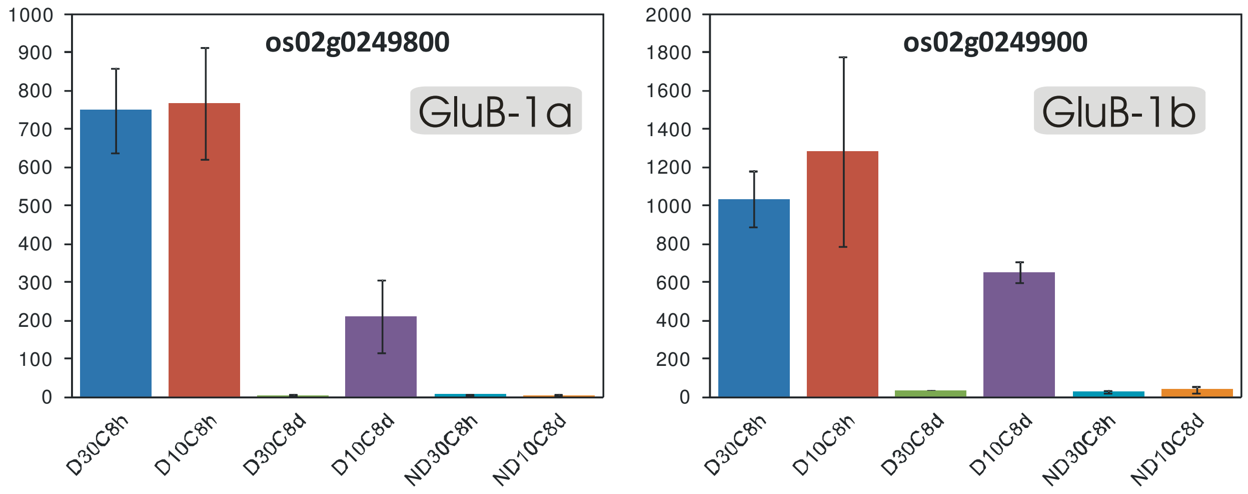

Figure In1. Expression levels, in the tested conditions, of genes for glutelin type B1 proteins, *GluB-1a* and *GluB-1b*. Error bars represent standard errors.

Glutelins are the most abundant storage proteins in rice grain and can make up to 80 % of total protein content (Qu and Takaiwa, 2004). As these proteins largely accumulate during seed development, their transcripts are not expected to be further translated in imbibed seeds. Regarding transcription, rice seed storage proteins genes are trans-activated by RISBZ1 (~ OsbZIP58) and RPBF (OsDof3), which act synergistically to give much higher levels of expression than the sum of individual activities elicited by each transcription factor alone (Yamamoto et al., 2006). Although both *RISBZ1* and *RPBF* showed higher expression in dormant seeds imbibed for 8 h (Fig. In2 of this Insight), novel transcription of *GluB-1a* and *GluB-1b*, and perhaps also other glutelin and seed storage protein genes, is repressed by OsGZF1 (Chen et al., 2014). Thus, a high level of expression of *OsGZF1* in dormant seeds at 8 h (Fig. In2 of this Insight), should assure that *de novo* expression of *GluB-1a* and *GluB-1b*, and other genes for seed storage proteins, was not activated in these seeds, and supports that the high expression observed for these genes was probably due to the persistence of their transcripts since seed development.

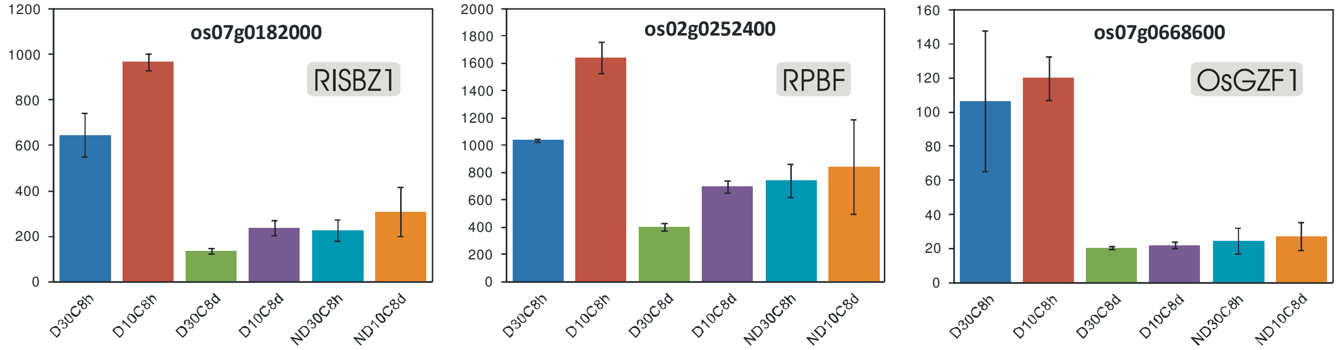

Figure In2. Expression levels, in the tested conditions, of genes for regulatory activators RISBZ1 and RPBF, and repressor OsGZF1. Error bars represent standard errors.

So, why did transcripts for glutelin type B1 proteins appear to be more expressed in dormant than in nondormant seeds? Perhaps, these mRNAs had been spontaneously degraded during afterripening, the process used to obtain nondormant seeds (El-Maarouf-Bouteau et al., 2013). If so, the same decrease should have been observed for all endosperm storage protein transcripts. Actually, several transcripts for other glutelins (GluA-1, GluA-2, GluA-3, GluB-2, GluC, GluD-1, GluB6 and Os02g0242600) as well as prolamins (genes Os07g0206500, Os07g0206400, Os03g0766100, Os06g0507200 and Os12g0269200), an albumin (gene Os07g0214300) and a globulin (gene Os05g0499100), showed similar, but much less sharp, expression patterns (Fig. In3 of this Insight). The gene for seed allergenic protein RAG2, which is a member of 14-16 kDa α-amylase/trypsin inhibitors in rice and belongs to the albumin storage proteins (Zhou et al., 2017), showed an analogous pattern of expression (Fig. In3 of this Insight). These findings support that spontaneous degradation of these mRNAs can have happened during afterripening, but the much sharper differential expression of *GluB-1a* and *GluB-1b* in dormant *vs* nondormant seeds, with respect to what observed for other storage protein genes, suggests it cannot be the only cause of this difference.

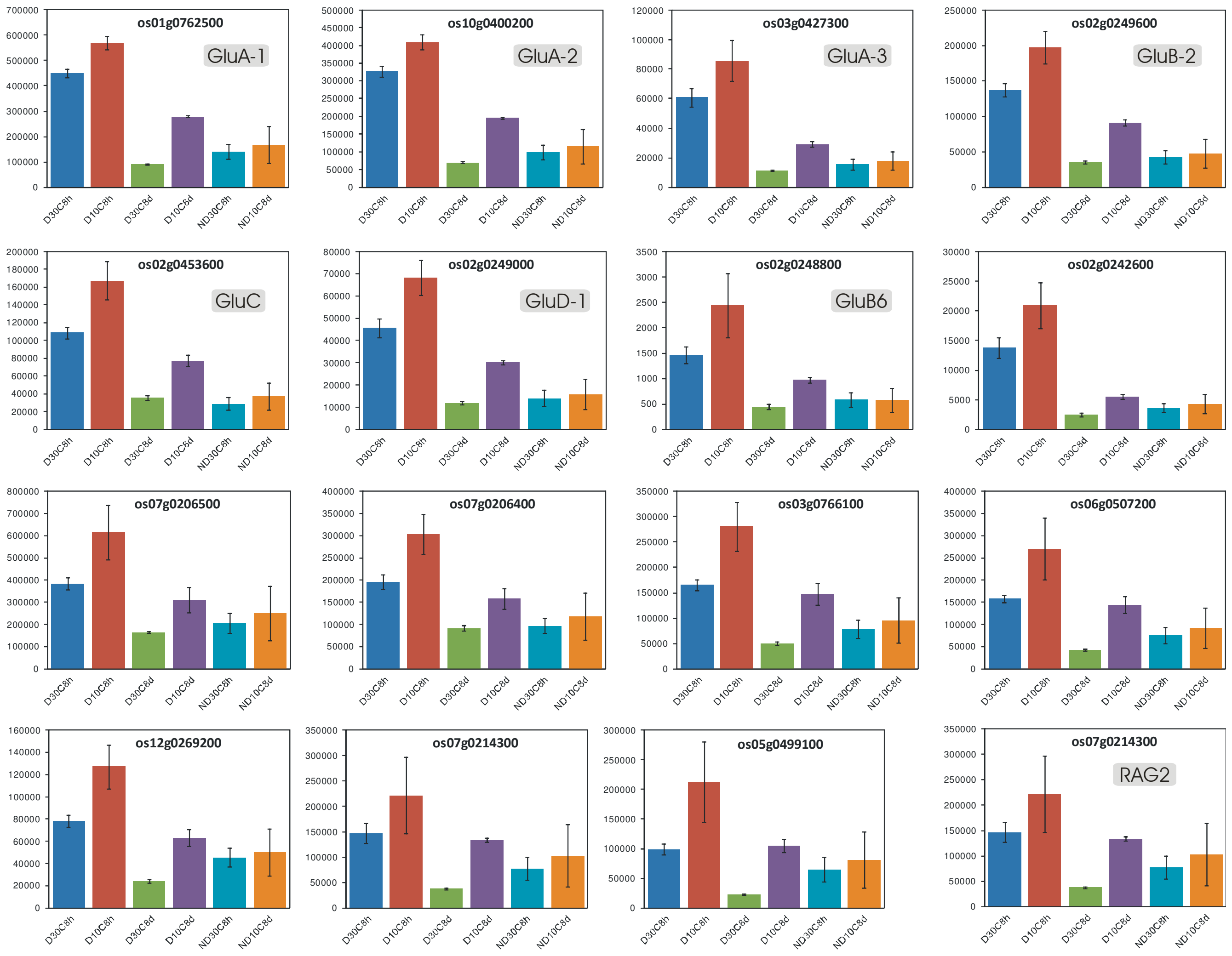

Figure In3. Expression levels, in the tested conditions, of genes for storage proteins: glutelins GluA-1, GluA-2, GluA-3, GluB-2, GluC, GluD-1, GluB6 and Os02g0242600; prolamins Os07g0206500, Os07g0206400, Os03g0766100, Os06g0507200, Os12g0269200; albumin Os07g0214300 and globulin Os05g0499100; and seed allergenic protein RAG2, which belongs to the albumin storage proteins and is also a member of 14-16 kDa α-amylase/trypsin inhibitors. Error bars represent standard errors. Many of these transcripts were the most abundant mRNAs across all the tested conditions (Supplemental Table S2).

Although RNA degradation consequent to afterripening could partially explain why all the transcripts listed in Fig. In3 (this Insight) displayed close expression patterns, it should be noted that, in rice (differently from arabidopsis), seed storage proteins are stored in the starchy endosperm, which is a dead tissue at the time of full maturation, though storage proteins and their transcripts are also present in the aleurone (Arcalis et al., 2014). Storage protein mRNAs are therefore preserved in the endosperm of the dry seed. Noteworthy, the promoter of at least one gene encoding for GluB-1 shows much higher activity in the region of the endosperm close to the scutellum (Qu and Takaiwa, 2004), thus that these

transcripts are expected to accumulate there. In addition, in the germinating cereal seed the initial formation site of hydrolases, proteinases and RNAases is in the region of the epithelial cells of the scutellum and only later it gradually extends throughout the aleurone layer into the entire region of the endosperm tissues (Okamoto et al., 1980). Furthermore, degradation of storage proteins in both embryo and endosperm in seeds of a wild rice was found to be closely associated with dormancy release (Xu et al., 2016). It can therefore be speculated that the apparent much higher levels of expression of *GluB-1a* and *GluB-1b* in imbibed dormant seeds at 8 h can rather be due to the biological degradation of these mRNAs in other samples, particularly nondormant seeds, wherein turnover of unused mRNAs is accelerated by the impending demand for the synthesis of new transcripts, starting from the embryo. Indeed, genes for glutelins GluA, GluB-2, GluC, GluD-1 and prolamins, are expressed in endosperm regions even far from embryo (Qu and Takaiwa, 2004; Kawakatsu et al., 2008; Qu et al., 2008). Differential activation of mRNA turnover in dormant and nondormant seeds could therefore justify why these genes appeared as DEGs. If the turnover starts in the embryo and then extends to the distal regions of the endosperm, in fact, a stronger decline would occur for mRNAs preferentially stored in tissues closer to the embryo. This could explain the stronger differentiation in the expression of *GluB-1a* and *GluB-1b* between dormant and nondormant seeds, that is, the hypothesized plunge of these transcripts in the latter seeds. Some turnover would occur also in dormant seeds incubated for 8 d at 10 °C, but this would still be much less than in nondormant seed incubated under the same conditions.

It can be mentioned that Os03g0197300, encoding for a RmlC-type domain containing cupin, showed a very high expression in dormant seeds, especially higher with respect to nondormant seeds incubated at 30 °C for 8 h (Fig. In4 of this Insight). The expression pattern of this gene was however different from that of all the other storage protein genes described here, suggesting that expression of Os03g0197300 differs for some feature that enhances preservation of its transcripts in dormant seeds. Interestingly, Os03g0197300 (LOC_Os03g10110), which has a maize homolog involved in embryo development, has been identified as a candidate regulator of rice seed dormancy in a genome-wide association study (Lu et al., 2018).

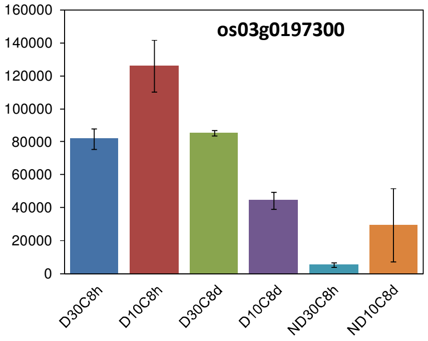

Figure In4. Expression levels, in the tested conditions, of gene for cupin storage protein Os03g0197300. Error bars represent standard errors.

A role of endosperm storage protein transcripts as a storage form of nucleotides would also be consistent with the fact that RNA from nondormant seeds resulted partially degraded even after 8 d at 10 °C. Clearly, most of the partially degraded RNA corresponded to an excess of unfunctional rRNA (compare the heights of the two highest peaks in Supplementary Fig. S1 A and B, corresponding to the 18S and 25S rRNA species, respectively) whose complete degradation was delayed. Although RNA synthesis and degradation are possible at this temperature, as demonstrated by the increase/decrease in the abundance of specific mRNAs in dormant seeds between 8 h and 8 d at 10 °C, a relevant presence

of degraded RNA in nondormant seeds still after 8 d at 10 °C suggests that partially degraded RNA was not completely degraded by further metabolic processing to nucleotides, undetectable by the RIN assay, most probably because there was not yet need of large amounts of nucleotides for the synthesis of novel RNAs. Untranslated long-lived mRNAs species present in the aleurone have to be fully degraded as activation of transcription, synthesis, and secretion of the enzymes needed to break down reserves gradually extends throughout the aleurone layer during germination. However, the dynamics of gene expression indicate that substantial mobilization of storage proteins deposited in the starchy endosperm and aleurone begins late during cereal seed germination (Yu et al., 2014). In addition, several partially degraded RNAs are, most probably, fettered in the starchy endosperm, where their use necessarily proceeds in parallel with the overall degradation of endosperm reserves. They would therefore still represent a storage form of nucleotides that is not further depleted because, in the absence of growth, they cannot be utilized. Although the dynamics of stored mRNAs in imbibing seeds need to be better explored in the future, the suggested explanation can make up for the puzzling findings described here.

References

Arcalis E., Ibl V., Peters J., Melnik S. and Stoger E. (2014). The dynamic behavior of storage organelles in developing cereal seeds and its impact on the production of recombinant proteins. Front. Plant Sci. 5:439.

Chen Y., Sun A., Wang M., Zhu Z. and Ouwerkerk P.B. (2014). Functions of the CCCH type zinc finger protein OsGZF1 in regulation of the seed storage protein GluB-1 from rice. Plant Mol. Biol. 84:621-634.

El-Maarouf-Bouteau H., Meimoun P., Job C., Job D. and Bailly C. (2013). Role of protein and mRNA oxidation in seed dormancy and germination. Front. Plant Sci. 4:77.

Galland M., Huguet R., Arc E., Cueff G., Job D. and Rajjou L. (2014). Dynamic proteomics emphasizes the importance of selective mRNA translation and protein turnover during *Arabidopsis* seed germination. Mol. Cell. Proteomics 13:252-268.

Kawakatsu T., Yamamoto M.P., Hirose S., Yano M. and Takaiwa F. (2008). Characterization of a new rice glutelin gene *GluD-1* expressed in the starchy endosperm. J. Exp. Bot. 59:4233-4245.

Lu Q., Niu X., Zhang M., Wang C., Xu Q., Feng Y., Yang Y., Wang S., Yuan X., Yu H., Wang Y., Chen X., Liang X. and Wei X. (2018). Genome-wide association study of seed dormancy and the genomic consequences of improvement footprints in rice (*Oryza sativa* L.). Front. Plant Sci. 8:2213.

Okamoto K., Kitano H. and Akazawa T. (1980). Biosynthesis and excretion of hydrolases in germinating cereal seeds. Plant Cell Physiol. 21:201-204.

Qu L.Q. and Takaiwa F. (2004). Evaluation of tissue specificity and expression strength of rice seed component gene promoters in transgenic rice. Plant Biotech. J. 2:113-125.

Qu L.Q., Xing Y.P., Liu W.X., Xu X.P., Song Y.R. (2008) Expression pattern and activity of six glutelin gene promoters in transgenic rice. J. Exp. Bot. 59:2417-2424.

Xu H.H., Liu S.J., Song S.H., Wang W.Q., Møller I.M. and Song S.Q. (2016). Proteome changes associated with dormancy release of Dongxiang wild rice seeds. J. Plant Physiol. 206:68-86.

Yamamoto M.P., Onodera Y., Touno S.M. and Takaiwa F. (2006). Synergism between RPBF Dof and RISBZ1 bZIP activators in the regulation of rice seed expression genes. Plant Physiol. 141:1694-1707.

Yu Y., Guo G., Lv D., Hu Y., Li J., Li X. and Yan Y. (2014). Transcriptome analysis during seed germination of elite Chinese bread wheat cultivar Jimai 20. BMC Plant Biol. 14:20.

Zhou W., Wang X., Zhou D., Ouyang Y. and Yao J. (2017). Overexpression of the 16-kDa α-amylase/trypsin inhibitor RAG2 improves grain yield and quality of rice. Plant Biotech. J. 15:568-580.
