# Supplementary material for: Seed Dormancy Involves a Transcriptional Program That Supports Early Plastid Functionality during Imbibition
Source: Plants (Basel). 2018 Apr 19;7(2):35. doi: 10.3390/plants7020035 (PMC6026906; doi:10.3390/plants7020035)
Supplement: Supplementary file 1 [file plants-07-00035-s001.zip › plants-273866-supplementary-update/Insight into pre-emptive defence strategies.pdf]

Supplementary material to the article "Seed dormancy involves a transcriptional program that supports early plastid functionality during imbibition" by Gianinetti et al.

Insight into pre-emptive defence strategies

As remarked in discussing JAs, several ethylene-responsive factors, which regulate stress-responsive networks in response to JA and other hormones even independently of ethylene, were differentially expressed in dormant and nondormant seeds. Since the latter showed a much higher expression of genes for JAs production, and the synergistic cross-talk between JA and ethylene signalling pathways is known to occur preferentially for the defense from necrotrophic pathogens (Wasternack and Hause, 2013), it ought to be expected that nondormant seeds activate this kind of defense processes. In fact, higher expression in nondormant seeds of trans-cinnamate 4-monooxygenase (Fig. 4), as well as of hydroxycinnamoyl transferase Os01g0854000 (a putative hydroxycinnamoyl-CoA shikimate/quinate hydroxycinnamoyl transferase; Fig. In1 of this Insight), suggests that defence substances like caffeoyl quinic acid (chlorogenic acid) and/or feruloyl quinic acid were preferentially formed in these seeds. The two acids are, in fact, involved in the resistance to herbivore insects and pathogens (Leiss et al., 2009; Leiss et al., 2011; Chamam et al., 2015; Maag et al., 2015).

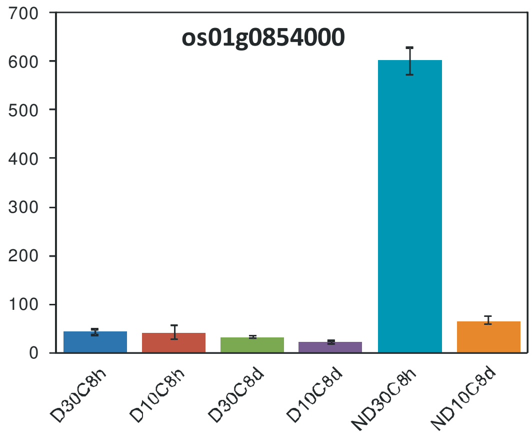

Figure In1. Expression levels, in the tested conditions, of gene for hydroxycinnamoyl transferase Os01g0854000, possibly involved in the synthesis of defense substances like caffeoyl quinic acid (chlorogenic acid) and/or feruloyl quinic acid. Error bars represent standard errors.

Nondormant seeds also showed a higher expression (Fig. In2 of this Insight) of genes for linoleate 9(S)-lipoxygenases *OsLOX1* and *LOX3*, involved in the metabolism of linoleic acid to form 9-oxylipins (Wang et al., 2008; Long et al., 2013), which have functions in development and are also largely recognized as defense agents against pests and pathogens (Christensen and Kolomiets, 2011).

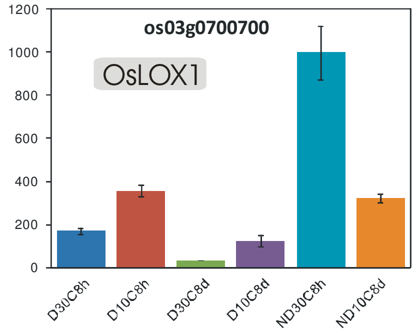

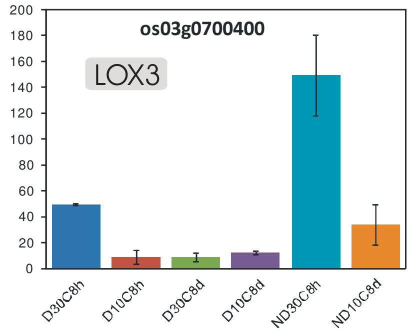

Figure In2. Expression levels, in the tested conditions, of genes for linoleate 9(S)-lipoxygenases OsLOX1 (chloroplastic) and LOX3 (cytosolic), involved in the metabolism of linoleic acid to form biotic defence agents 9-oxylipins. Error bars represent standard errors.

In accordance with these findings, the OsMKK4-OsMPK1-OsWRKY53 module, positively regulating biosynthesis of antimicrobials like rice-specific diterpenoid phytoalexins and lignin as well as genes for glycolysis (Kishi-Kaboshi et al., 2010) was more expressed in nondormant seeds (Fig. In3 of this Insight). OsMPK1 (~ OsSIPK ~ OsMAPK6) is a protein kinase that acts as a central master switch for stress responses, also integrating fine-tuned regulation by JA (Cho et al., 2009). OsMKK4, the upstream kinase of OsMPK1, phosphorylates OsMPK1 to activate the module (Kishi-Kaboshi et al., 2010). OsWRKY53 is phosphorylated by OsMPK1 to activate plant disease response (Chujo et al., 2014) and can be a negative regulator of plant growth (Hu et al., 2015), but it also functions as a negative feedback modulator of OsMPK6 and thereby controls the size of the investment a rice plant makes to defend against chewing herbivory (Hu et al., 2015). So, the specific pre-emptive defence strategy of nondormant red rice seeds would involve activation of biosynthesis genes for caffeoyl and/or feruloyl quinic acids and oxylipins, though modulated according to a tradeoff between defence and growth, apparently by means of the OsMKK4-OsMPK1-OsWRKY53 regulation module.

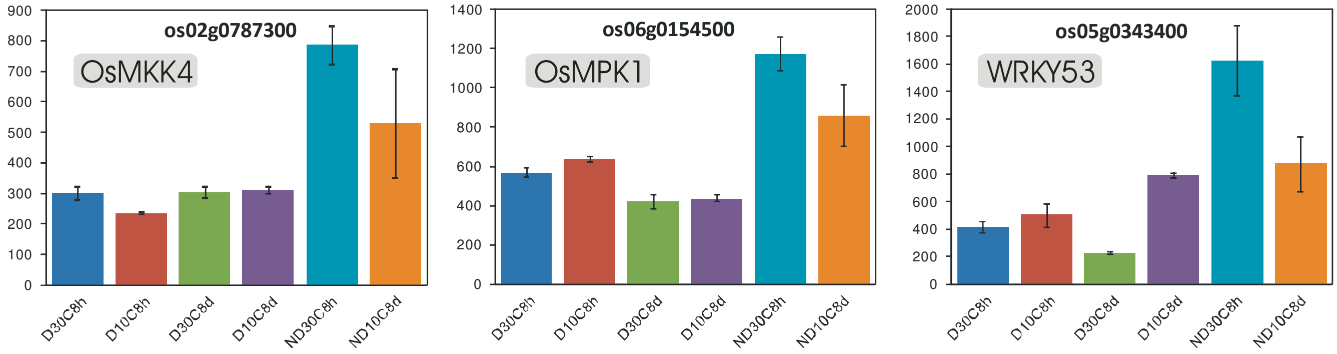

Figure In3. Expression levels, in the tested conditions, of genes of the regulative module for the transcription of genes for biosynthesis of phytoalexins and lignin as well as for glycolysis: OsMKK4 phosphorylates OsMPK1, which is thereby activated and, in turn, phosphorylates proteins involved in plant disease response, like OsWRKY53, to activate stress responses. Error bars represent standard errors.

On the other hand, *OsNPR1/NH1*, a rice homolog of *NPR1*, the key regulator of systemic acquired resistance in arabidopsis, was more expressed in dormant seeds at 8 d of incubation (Fig. In4 of this Insight). OsNPR1 confers disease resistance (Feng et al., 2011), but negatively regulates herbivore-induced JA and ethylene signalling and, thereby, reduces plant resistance response to insect herbivores (Yuan et al., 2007; Li et al., 2013). It also switches the tradeoff between pathogen defence and photosynthesis reallocation energy and resources during defence responses by downregulating genes involved in photosynthesis and in plastid activity while upregulating the expression of defence genes (Sugano et al., 2010). OsNPR1 also attenuates growth by repressing auxin signalling through promoting expression of IAA-amido synthases like OsGH3.8 (Fig. In4 of this Insight; Li et al., 2016), which deplete free auxin by conjugating it to amino acids. It would therefore appear that, following imbibition, dormant seeds stabilize their metabolism towards an idle but “vigil” state.

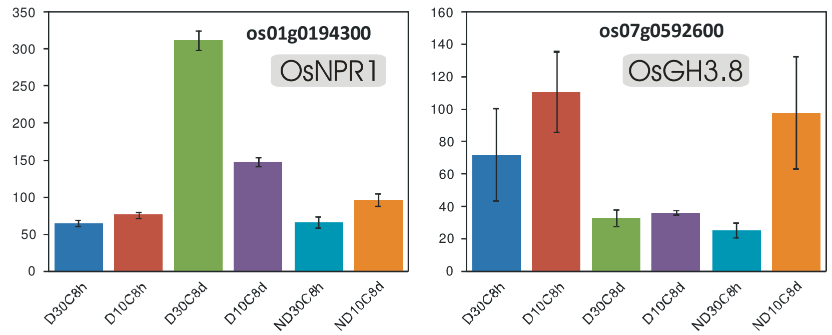

Figure In4. Expression levels, in the tested conditions, of genes for OsNPR1, involved in systemic acquired resistance, and IAA-amido synthases OsGH3.8, conjugating free auxin to amino acids. Error bars represent standard errors.

Correspondingly, from imbibition (8 h) to stable metabolism (8 d) dormant seeds decreased the expression of genes involved in plastid activities and abiotic stresses while increased that of genes involved in biotic stresses (Supplementary Fig. S5), with a large increase in pathogenesis-related transcripts. Specifically, genes Os02g0571100 for ent-copalyl diphosphate synthases (OsCPS2 ~ OsCyc2, which is duplicated in locus Os02g0570900) and Os02g0570400 for ent-cassa-12,15-diene synthase (OsDTC1 ~ OsKS3 ~ KSL7), which catalyze the first two steps of the biosynthesis of the phytocassane family of rice antifungal phytoalexins (Cho et al., 2004; Otomo et al., 2004), were upregulated at 8 d (Fig. In5 of this Insight).

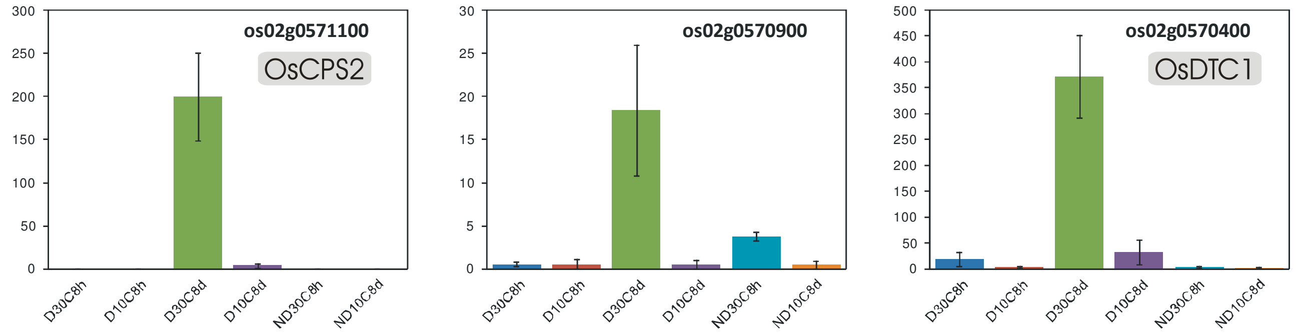

Figure In5. Expression levels, in the tested conditions, of genes for pathogenesis-related transcripts: *OsCPS2*, for ent-copalyl diphosphate synthase, is duplicated in locus Os02g0570900; *OsDTC1* encodes for ent-cassa-12,15-diene synthase, which catalyzes the first two steps of phytocassane synthesis. Error bars represent standard errors.

In addition, though not detected as DEGs, genes for syn-copalyl diphosphate synthase (OsCPS4 ~ OsCyc1) and 9-β-pimara-7,15-diene oxidase OsKS4, which are key enzymes for biosynthesis of momilactone phytoalexins (Otomo et al., 2004; Prisic et al., 2004; Xu et al., 2004; Shimura et al., 2007) were increased too (Fig. In6 of this Insight). A relevant exception to the down-regulation of genes for abiotic stresses during metabolism stabilization was gene Os11g0703900 for a DnaK-type molecular chaperone hsp70, which was one of the overall most highly expressed genes (Supplemental Table S2); in fact, expression of this abiotic stress gene strongly increased after 8 d of incubation at 30 °C in dormant seeds (Fig. In6 of this Insight). Os11g0703900 is orthologous to arabidopsis *HSP70B*, which was strongly down-regulated by afterripening whereas remaining expressed in dormant seeds (Carrera

et al., 2008). In arabidopsis, abundance of stress response proteins in dormant seeds was suggested to contribute to their survival (Arc et al., 2012).

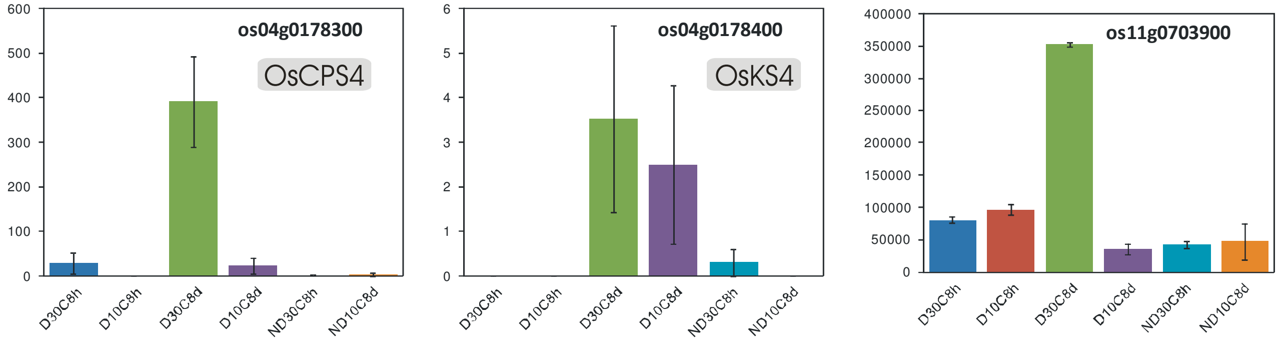

Figure In6. Expression levels, in the tested conditions, of genes for: syn-copalyl diphosphate synthase OsCPS4 and 9-β-pimara-7,15-diene oxidase OsKS4, which are involved in the biosynthesis of momilactones; Os11g0703900 encodes for a DnaK-type molecular chaperone hsp70. Error bars represent standard errors.

As dormant seeds showed an up-regulation of genes for synthesis of PAs (Fig. 4), which protect seeds from pathogens (Dixon et al., 2005), the specific pre-emptive defense strategy of dormant red rice seeds would involve accumulation of PAs first, for mechanical protection, followed by activation of biosynthesis genes for phytocassane and momilactone antimicrobials, together with hsp70 chaperone, involved in various cellular processes including protein folding and helping to protect cells from stress. This may be associated with expression of the OsNPR1 regulator.

Hormonal interactions are known to be complex, and this is particularly evident for stress responses. Although an antagonistic cross-talk between JAs and GAs would be expected, as plant growth and defence responses are usually antagonistic because plant defence occurs at the expense of plant growth (Wasternack and Hause, 2013), active GAs integrate and amplify JA-dependent innate immunity in rice (De Vleesschauwer et al., 2016). This is apparent from an overall transcriptional activation of both JAs- and GAs-related genes in nondormant seeds early (8 h) during imbibition (Figures 5 and 8). Thus, present findings reveal that the tuning of the pre-emptive defence strategy differs between dormant and nondormant seeds, in association with a diverse transcriptional regulation of JA and GAs genes.

References

Arc E., Chibani K., Grappin P., Jullien M., Godin B., Cueff G., Valot B., Balliau T., Job D. and Rajjou L. (2012) Cold stratification and exogenous nitrates entail similar functional proteome adjustments during *Arabidopsis* seed dormancy release. J. Proteome Res. 11:5418-5432.

Carrera E., Holman T., Medhurst A., Dietrich D., Footitt S., Theodoulou F. L. and Holdsworth M. J. (2008). Seed after-ripening is a discrete developmental pathway associated with specific gene networks in Arabidopsis. Plant J. 53:214-224.

Chamam A., Wisniewski-Dyé F., Comte G., Bertrand C. and Prigent-Combaret C. (2015). Differential responses of *Oryza sativa* secondary metabolism to biotic interactions with cooperative, commensal and phytopathogenic bacteria. Planta 242:1439-1452.

Cho K., Agrawal G.K., Jwa N.S., Kubo A. and Rakwal R. (2009). Rice OsSIPK and its orthologs: A “central master switch” for stress responses. Biochem. Biophys. Res. Commun. 379:649-653.

Cho E.M., Okada A., Kenmoku H., Otomo K., Toyomasu T., Mitsuhashi W., Sassa T., Yajima A., Yabuta G., Mori K. and Oikawa H. (2004). Molecular cloning and characterization of a cDNA encoding ent-cassa-12,15-diene synthase, a putative diterpenoid phytoalexin biosynthetic enzyme, from suspension-cultured rice cells treated with a chitin elicitor. Plant J. 37:1-8.

Christensen S.A. and Kolomiets M.V. (2011). The lipid language of plant–fungal interactions. Fungal Genet. Biol. 48:4-14.

Chujo T., Miyamoto K., Ogawa S., Masuda Y., Shimizu T., Kishi-Kaboshi M., Takahashi A., Nishizawa Y., Minami E., Nojiri H. and Yamane H. (2014). Overexpression of phosphomimic mutated OsWRKY53 leads to enhanced blast resistance in rice. PLoS One 9:e98737.

De Vleesschauwer D., Seifi H.S., Filipe O., Haeck A., Huu S.N., Demeestere K. and Höfte M. (2016). The DELLA protein SLR1 integrates and amplifies salicylic acid-and jasmonic acid-dependent innate immunity in rice. Plant Physiol. 170:1831-1847.

Dixon R.A., Xie, D.Y. and Sharma S.B. (2005). Proanthocyanidins–a final frontier in flavonoid research?. New Phytol. 165:9-28.

Feng J.X., Cao L., Li J., Duan C.J., Luo X.M., Le N., Wei H., Liang S., Chu C., Pan Q. and Tang, J.L. (2011). Involvement of OsNPR1/NH1 in rice basal resistance to blast fungus *Magnaporthe oryzae*. Eur. J. Plant Pathol. 131:221-235.

Hu L., Ye M., Li R., Zhang T., Zhou G., Wang Q., Lu J. and Lou Y. (2015). The rice transcription factor WRKY53 suppresses herbivore-induced defenses by acting as a negative feedback modulator of mitogen-activated protein kinase activity. Plant Physiol. 169:2907-2921.

Kishi-Kaboshi M., Okada K., Kurimoto L., Murakami S., Umezawa T., Shibuya N., Yamane H., Miyao A., Takatsuji H., Takahashi A. and Hirochika H. (2010). A rice fungal MAMP-responsive MAPK cascade regulates metabolic flow to antimicrobial metabolite synthesis. Plant J. 63:599-612.

Leiss K.A., Choi Y.H., Verpoorte R. and Klinkhamer P.G. (2011). An overview of NMR-based metabolomics to identify secondary plant compounds involved in host plant resistance. Phytochem. Rev. 10:205-216.

Leiss K.A., Maltese F., Choi Y.H., Verpoorte R. and Klinkhamer P.G.L. (2009). Identification of chlorogenic acid as a resistance factor for thrips in Chrysanthemum. Plant Physiol. 150:1567-1575.

Li R., Afsheen S., Xin Z., Han X. and Lou Y. (2013). OsNPR1 negatively regulates herbivore-induced JA and ethylene signaling and plant resistance to a chewing herbivore in rice. Physiol. Plant. 147:340-351.

Li X., Yang D., Sun L., Li Q., Mao B. and He Z. (2016). The systemic acquired resistance regulator OsNPR1 attenuates growth by repressing auxin signaling and promoting IAA-amido synthase expression. Plant Physiol. 172:546-558.

Long Q., Zhang W., Wang P., Shen W., Zhou T., Liu N., Wang R., Jiang L., Huang J., Wang Y. and Liu Y. (2013). Molecular genetic characterization of rice seed lipoxygenase 3 and assessment of its effects on seed longevity. J. Plant Biol. 56:232-242.

Maag D., Erb M. and Glauser G. (2015). Metabolomics in plant–herbivore interactions: challenges and applications. Entomol. Exp. Appl. 157:18-29.

Otomo K., Kenmoku H., Oikawa H., Konig W.A., Toshima H., Mitsuhashi W., Yamane H., Sassa T., Toyomasu T. (2004). Biological functions of ent- and syn-copalyl diphosphate synthases in rice: key enzymes for the branch point of gibberellin and phytoalexin biosynthesis. Plant J. 39:886-893.

Prisic S., Xu M., Wilderman P.R. and Peters R.J. (2004). Rice contains two disparate ent-copalyl diphosphate synthases with distinct metabolic functions. Plant Physiol. 136:4228-4236.

Shimura K., Okada A., Okada K., Jikumaru Y., Ko K.W., Toyomasu T., Sassa T., Hasegawa M., Kodama O., Shibuya N. and Koga J. (2007). Identification of a biosynthetic gene cluster in rice for momilactones. J. Biol. Chem. 282:34013-34018.

Sugano S., Jiang C.J., Miyazawa S.I., Masumoto C., Yazawa K., Hayashi N., Shimono M., Nakayama A., Miyao M. and Takatsuji H. (2010). Role of OsNPR1 in rice defense program as revealed by genome-wide expression analysis. Plant Mol. Biol. 74:549-562.

Wang R., Shen W., Liu L., Jiang L., Liu Y., Su N. and Wan J. (2008). A novel lipoxygenase gene from developing rice seeds confers dual position specificity and responds to wounding and insect attack. Plant Mol. Biol. 66:401-414.

Wasternack C. and Hause B. (2013). Jasmonates: biosynthesis, perception, signal transduction and action in plant stress response, growth and development. An update to the 2007 review in Annals of Botany. Ann. Bot. 111:1021-1058.

Xu M., Hillwig M.L., Prisic S., Coates R.M. and Peters R.J. (2004). Functional identification of rice syn-copalyl diphosphate synthase and its role in initiating biosynthesis of diterpenoid phytoalexin/allelopathic natural products. Plant J. 39:309-318.

Yuan Y., Zhong S., Li Q., Zhu Z., Lou Y., Wang L., Wang J., Wang M., Li Q., Yang D. and He Z. (2007). Functional analysis of rice *NPR1*-like genes reveals that *OsNPR1/NH1* is the rice orthologue conferring disease resistance with enhanced herbivore susceptibility. Plant Biotech. J. 5:313-324.
