# Supplementary material for: Seed Dormancy Involves a Transcriptional Program That Supports Early Plastid Functionality during Imbibition
Source: Plants (Basel). 2018 Apr 19;7(2):35. doi: 10.3390/plants7020035 (PMC6026906; doi:10.3390/plants7020035)
Supplement: Supplementary file 1 [file plants-07-00035-s001.zip › plants-273866-supplementary-update/Insight into the carbon metabolism.pdf]

Supplementary material to the article "Seed dormancy involves a transcriptional program that supports early plastid functionality during imbibition" by Gianinetti et al.

Insight into the carbon metabolism

<u>The glyoxylate cycle</u>

In many seeds, including those of rice (He et al., 2011), following imbibition, metabolism starts by means of the glyoxylate cycle, a pathway localized into glyoxysomes that allows the seed to use lipids, previously stored in oleosomes and first subjected to β-oxidation of fatty acids, as both a source of carbon skeletons to replenish the intermediates of the main metabolic pathways involved in biosynthetic processes as well as an initial source of energy (Oaks and Beevers, 1964; Bewley, 2001; Donaldson et al., 2008). Likewise to the TCA cycle, this cycle begins with a condensation of acetyl-CoA with oxaloacetate to form citrate, but, differently from the TCA, the glyoxylate cycle bypasses the decarboxylation reactions, thus permitting the acetyl-CoAs to be ultimately used for gluconeogenesis (i.e., the generation of glucose, or, at least, of its precursor phosphoenolpyruvate, from non-carbohydrate organic substrates) without carbon losses (Bewley, 2001).

In cereals, the glyoxylate cycle was observed in the aleurone (Clarke et al., 1983; Holtman et al., 1994; Lu et al., 2013) and scutellum (Oaks and Beevers, 1964; Longo and Longo, 1970; Ma et al., 2016). Thus, although the embryo axis of cereals is also rich in oil, it appears unable to use oil as a gluconeogenic substrate because these tissues lack the glyoxylate cycle enzymes isocitrate lyase and malate synthase (Holtman et al., 1994). In the embryo axis, oil bodies may instead represent a respiratory substrate and therefore an energy source, or they can provide fatty acids for the synthesis of membrane lipids (Clarke et al., 1983; Eastmond and Jones, 2005).

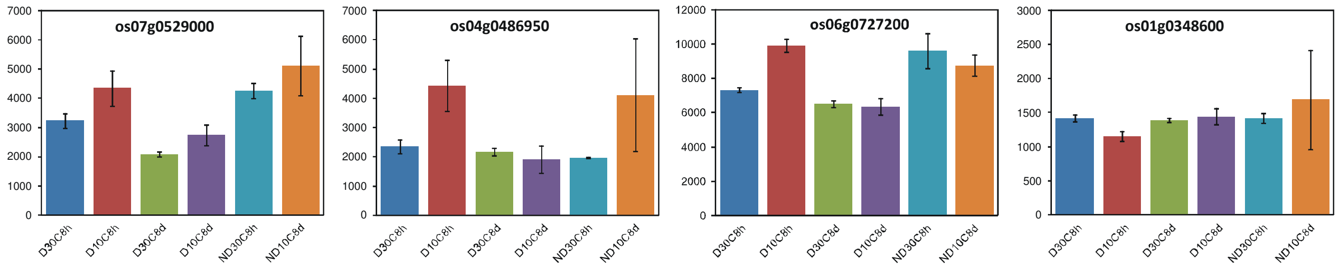

Figure In1. Expression levels, in the tested conditions, of genes for enzymes of the glyoxylate cycle: isocitrate lyase Os07g0529000, malate synthase Os04g0486950, catalase Os06g0727200, glyoxysomal fatty acid β-oxidation multifunctional protein Os01g0348600. Error bars represent standard errors.

In red rice seeds, transcripts for isocitrate lyase and malate synthase (key enzymes involved in the glyoxylate cycle), as well as for glyoxysome catalase (which is required to break down hydrogen peroxide that is formed because of glyoxysome inefficient β-oxidation of fatty acids that uses oxygen for reoxidation of $FADH_2$) and a fatty acid β-oxidation multifunctional protein with 3-hydroxyacyl-CoA dehydrogenase activity (which produces NADH during the successive cycles of fatty acid

shortening in β-oxidation), were highly expressed in all conditions (thus that they were not detected as DEGs, but are anyway shown in Fig. In1 of this Insight to support the activation of this pathway).

As for the synthesis of membrane lipids, diglyceride acyltransferase Os06g0563900, which catalyzes the formation of triglycerides from diacylglycerol and acyl-CoA and is considered the final, rate-limiting and only dedicated step in triglyceride synthesis, was more expressed in dormant seeds at 8 h of imbibition (at both 30 °C and 10 °C) and at 8 d of imbibition at 30 °C (Fig. In2 of this Insight).

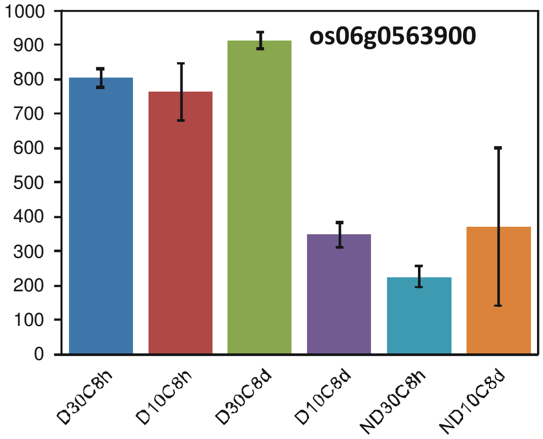

Figure In2. Expression levels, in the tested conditions, of gene for diglyceride acyltransferase Os06g0563900 catalyzes the formation of triglycerides from diacylglycerol and acyl-CoA. Error bars represent standard errors.

In addition to convert acetyl-CoA obtained from β-oxidation of fatty acids into the primary carbon source of the organism (through C_4 dicarboxylic acids that can enter gluconeogenesis), the glyoxylate cycle also allows plants to utilize acetate both as a carbon source and as a source of energy (Oaks and Beevers, 1964; Miro and Ismail, 2013). In fact, aldehyde dehydrogenases can convert acetaldehyde produced by glycolysis under hypoxic conditions (or under a hypoxic-like stress caused by poor mitochondrial functionality) to acetate and then acetyl-CoA synthetase can produce acetyl-CoA in the glyoxysome (Miro and Ismail, 2013). The expression of the respective genes is reported in Figures In3 and In4 of this Insight.

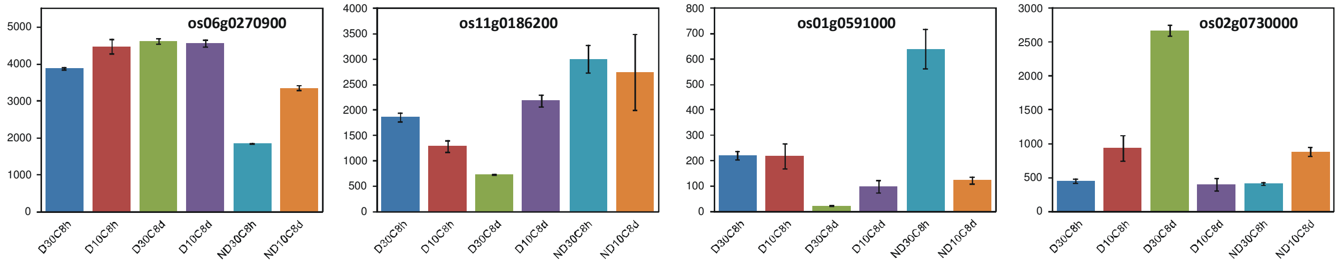

Figure In3. Expression levels, in the tested conditions, of genes for enzymes involved in acetate production and use in gluconeogenesis: aldehyde dehydrogenases Os06g0270900 (ALDH2b, mitochondrial), Os11g0186200, Os01g0591000 (cytosolic) and Os02g0730000 (ALDH2a, mitochondrial). Error bars represent standard errors.

Although, in anaerobic metabolism, metabolization of acetaldehyde to ethanol (alcoholic fermentation) is necessary to provide energy, the most favorable pathway is probably the production of acetate to remove toxic acetaldehyde and to recycle carbon for use in other pathways such the glyoxylate cycle, and to feed TCA cycle intermediates like malate or citrate (Miro and Ismail, 2013). Figure In3 shows

that at least one cytosolic aldehyde dehydrogenase (more effective than the mitochondrial enzyme when the mitochondrion respiratory chain is impaired) was indeed more expressed in nondormant seeds early after imbibition (8 h at 30 °C), confirming that the long dry-afterripening used to obtain nondormant seeds caused, in these seeds, a stronger impairment in mitochondrial functionality.

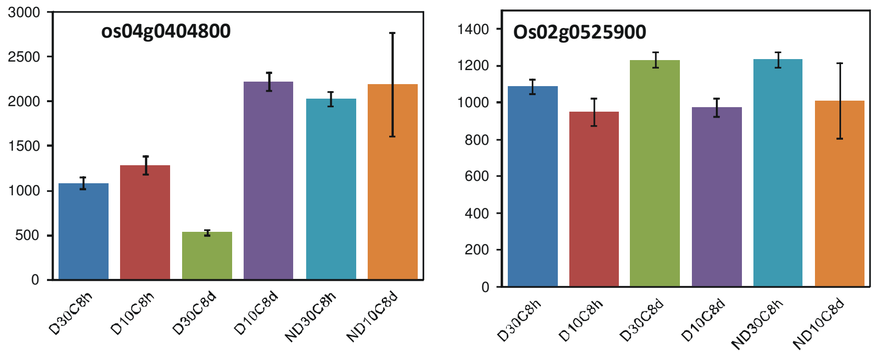

Figure In4. Expression levels, in the tested conditions, of genes for enzymes involved in acetate production and use in gluconeogenesis: acetyl-CoA synthetases Os04g0404800 and Os02g0525900 (not a DEG, included for completeness). Error bars represent standard errors.

The TCA cycle

The glyoxylate cycle can also provide intermediates to power up the TCA cycle, mainly succinate (Miro and Ismail, 2013). Indeed, transcripts and enzyme activities associated with TCA cycle, particularly malate dehydrogenase, are highly increased during early germination (Rosental et al., 2014). In fact, isocitrate, malate and succinate dehydrogenases (Os04g0479200, Os08g0120000 and Os08g0434300, respectively) were all up-regulated in red rice nondormant seeds (Fig. In5 of this Insight). Another malate dehydrogenase (Os03g0773800) was, instead, up-regulated in dormant seeds (Fig. In5 of this Insight), but this could also be expected given that such enzyme activity is necessary for the glyoxylate cycle and/or gluconeogenesis (depending on the intracellular compartmentalization of this enzyme). On the one side, the former findings confirm that recover of respiration is more important in nondormant seeds. On the other side, the latter finding is in accordance with a change of the primary metabolism in dormant seeds towards gluconeogenesis.

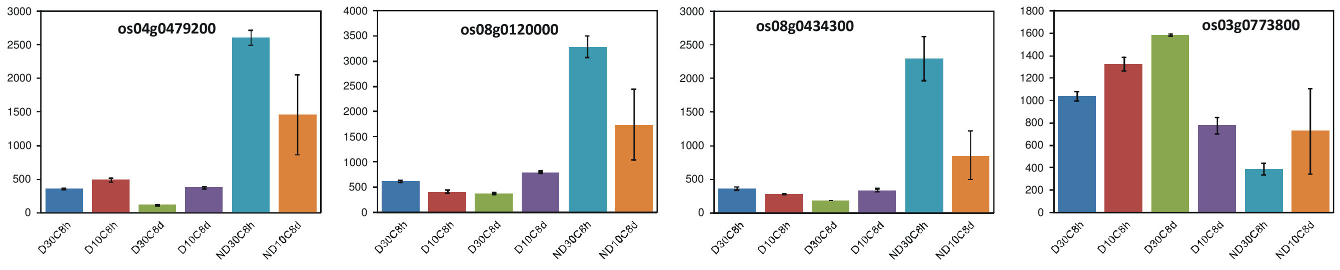

Figure In5. Expression levels, in the tested conditions, of genes for enzymes of the TCA cycle: isocitrate dehydrogenase Os04g0479200, malate dehydrogenases Os08g0120000 and Os03g0773800, and succinate dehydrogenase Os08g0434300. Error bars represent standard errors.

Carbon transfer

Typically, carbon from endospermic reserves is transported to the embryo to fuel postgerminative growth in the form of sucrose (Nomura et al., 1969; Bewley, 2001). It has also been suggested that following imbibition, in cereal seeds, the sucrose initially targeted to the embryo to fuel metabolism is derived from soluble sugars (Yu et al., 2014). Sucrose constitutes an important reserve in the scutellum and it is mobilized during the first two hydration phases of the germination process (Sánchez-Linares et al., 2012). However, since even nitrogen from degradation of storage proteins has to be transferred from the endosperm to the embryo axis, amino acids like glutamine and asparagine are transported as well (Bewley, 2001).

In the cereal aleurone, oil catabolism would be required to produce the energy and carbon skeletons necessary to initiate the production and secretion of hydrolases by the aleurone to mobilize endosperm reserves and sustain growth of the germinating seed (Eastmond and Jones, 2005). In such tissue, accordingly, the glyoxylate cycle is specifically activated during germination by embryo-produced gibberellins (Doig et al., 1975; Eastmond and Jones, 2005). In the dormant red rice seed, the scutellum could then grant gluconeogenesis to the embryo axis, which, as seen, should not have an active glyoxylate cycle.

References

Bewley J.D. (2001). Seed germination and reserve mobilization. In: eLS, Encyclopedia of Life Sciences. John Wiley & Sons, Ltd, Chichester.

Clarke N.A., Wilkinson M.C. and Laidman D.L. (1983). Lipid metabolism in germinating cereals. In: Lipids in Cereal Technology, P.J. Barnes ed. Academic Press, London, pp. 57-92.

Doig R.I., Colborne A.J., Morris G. and Laidman D.L. (1975). The induction of glyoxysomal enzyme activities in the aleurone cells of germinating wheat. J. Exp. Bot. 26:387-398.

Donaldson R.P., Kwak Y., Yanik T. and Sharma V. (2008). Plant peroxisomes and glyoxysomes. In: eLS, Encyclopedia of Life Sciences. John Wiley & Sons, Ltd, Chichester.

Eastmond P.J. and Jones R.L. (2005). Hormonal regulation of gluconeogenesis in cereal aleurone is strongly cultivar-dependent and gibberellin action involves SLENDER1 but not GAMYB. Plant J. 44:483-493.

He D., Han C., Yao J., Shen S. and Yang P. (2011). Constructing the metabolic and regulatory pathways in germinating rice seeds through proteomic approach. Proteomics 11:2693-2713.

Holtman W.L., Heistek J.C., Mattern K.A., Bakhuizen R. and Douma A.C. (1994). β-oxidation of fatty acids is linked to the glyoxylate cycle in the aleurone but not in the embryo of germinating barley. Plant Sci. 99:43-53.

Longo C.P. and Longo G.P. (1970). The development of glyoxysomes in peanut cotyledons and maize scutella. Plant Physiol. 45:249-254.

Lu X., Chen D., Shu D., Zhang Z., Wang W., Klukas C., Chen L.-l., Fan Y., Chen M. and Zhang C. (2013). The differential transcription network between embryo and endosperm in the early developing maize seed. Plant Physiol. 162:440-455.

Ma Z., Marsolais F., Bernards M.A., Sumarah M.W., Bykova N.V. and Igamberdiev A.U. (2016). Glyoxylate cycle and metabolism of organic acids in the scutellum of barley seeds during germination. Plant Sci. 248:37-44.

Miro B. and Ismail A.M. (2013). Tolerance of anaerobic conditions caused by flooding during germination and early growth in rice (*Oryza sativa* L.). Front. Plant Sci. 4:269.

Nomura T., Kono Y. and Akazawa T. (1969). Enzymic mechanism of starch breakdown in germinating rice seeds II. Scutellum as the site of sucrose synthesis. Plant Physiol. 44:765-769.

Oaks A. and Beevers H. (1964). The glyoxylate cycle in maize scutellum. Plant Physiol. 39:431-434.

Rosental L., Nonogaki H. and Fait A. (2014). Activation and regulation of primary metabolism during seed germination. Seed Sci. Res. 24:1-15.

Sánchez-Linares L., Gavilanes-Ruíz M., Díaz-Pontones D., Guzmán-Chávez F., Calzada-Alejo V., Zurita-Villegas V., Luna-Loaiza V., Moreno-Sánchez R., Bernal-Lugo I. and Sánchez-Nieto S. (2012). Early carbon mobilization and radicle protrusion in maize germination. J. Exp. Bot. 63:4513-4526.

Yu Y., Guo G., Lv D., Hu Y., Li J., Li X. and Yan Y. (2014). Transcriptome analysis during seed germination of elite Chinese bread wheat cultivar Jimai 20. BMC Plant Biol. 14:20.
