# Supplementary material for: Seed Dormancy Involves a Transcriptional Program That Supports Early Plastid Functionality during Imbibition
Source: Plants (Basel). 2018 Apr 19;7(2):35. doi: 10.3390/plants7020035 (PMC6026906; doi:10.3390/plants7020035)
Supplement: Supplementary file 1 [file plants-07-00035-s001.zip › plants-273866-supplementary-update/Insight into the hypoxic-like stress caused by dry-afterripening.pdf]

Supplementary material to the article "Seed dormancy involves a transcriptional program that supports early plastid functionality during imbibition" by Gianinetti et al.

Insight into the hypoxic-like stress caused by dry-afterripening

During imbibition, rice embryos show a transition from unstructured promitochondria to the typical cristae-rich mitochondrial structures (Howell et al., 2006). Although mitochondria from dry seeds have all tricarboxylic acid (TCA) cycle enzymes and are able to phosphorylate and produce some ATP, they are usually underdeveloped, and full mitochondrial gain of functions gradually occurs during the first hours of imbibition (Rosental et al., 2014). Specifically, mitochondria in dry seeds need repair and differentiation during imbibition before they can fully restore oxidative phosphorylation (Weitbrecht et al., 2011). In fact, in rice, the functionality of the respiratory chain is initially impaired when seeds are re-hydrated from the dry state and it increases only after 12 h of imbibition (Howell et al., 2006).

In red rice, a general indication that a stronger hypoxic-like stress occurred during imbibition of afterripened seeds was a much higher gene expression of *HB2*, encoding for a non-symbiotic hemoglobin (Fig. In1 of this Insight). Non-symbiotic hemoglobins are indeed induced under several stress conditions, like hypoxia (Matilla and Rodríguez-Gacio, 2013).

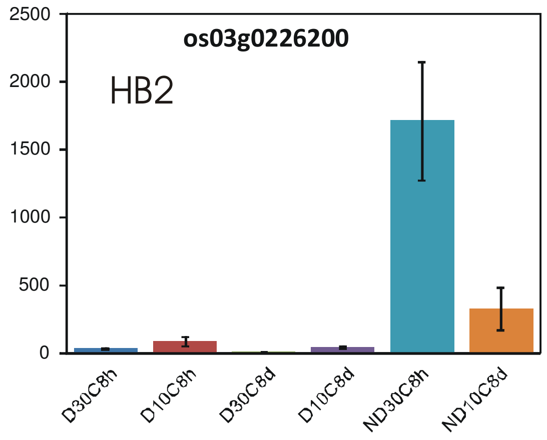

Figure In1. Expression levels, in the tested conditions, of gene for non-symbiotic hemoglobin HB2. Error bars represent standard errors.

As the respiratory chain is more or less impaired upon seed imbibition, the oxidative phosphorylation of NADH and $FADH_2$ generated in the TCA cycle is, at least partially, blocked (Botha et al., 1992), and genes for alternative dehydrogenases dissipating NADH excess are therefore actively expressed during imbibition (Howell et al., 2006). Again, this was expected to be more relevant in dry-afterripened seeds. In fact, the expressions of Os07g0564500, enconding for an alternative (non-electrogenic) NAD(P)H:ubiquinone reductase, and of *OsAOX1a*, encoding for a mitochondrial electron transport alternative oxidase, i.e. a non-electrogenic ubiquinol oxidase, involved in the scavenging of reactive oxygen species through the so called cyanide-resistant respiration, were high in afterripened nondormant seeds upon 8 h of imbibition at 30 °C (Fig. In2 of this Insight). Expression of the latter gene was however more enhanced in response to a prolonged cold treatment in both dormant and nondormant seeds (Fig. In2 of this Insight).

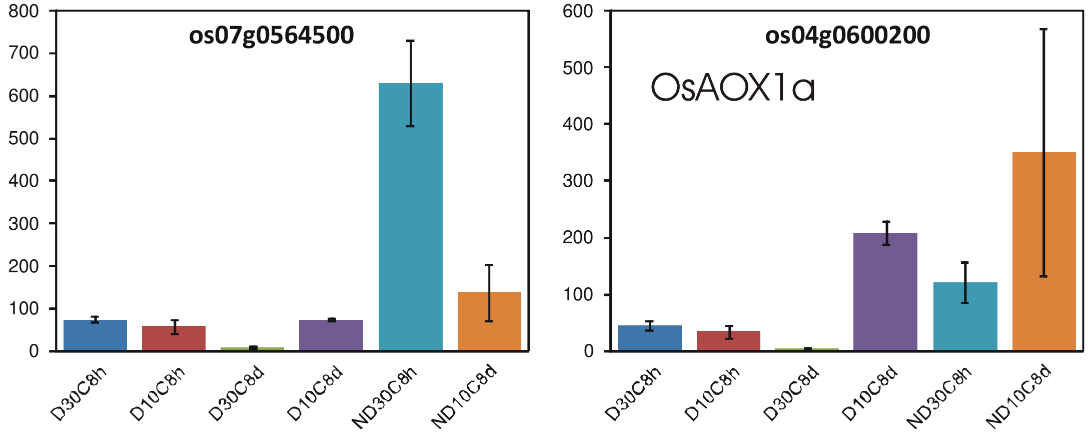

Figure In2. Expression levels, in the tested conditions, of genes for NADH-dissipating dehydrogenases: alternative NADH:ubiquinone reductase Os07g0564500 and alternative oxidase OsAOX1a. Error bars represent standard errors.

Mitochondrial energy-dissipating systems are expected to have an important role in stress tolerance by preventing over-reduction of electron carriers that would otherwise lead to oxidative stress (van Dongen et al., 2011). Indeed, much of the control of respiration is vested in the mitochondrial electron transport chain itself (van Dongen et al., 2011). The high expression of these genes, particularly Os07g0564500, therefore confirms the occurrence of a hypoxic-like stress during imbibition of afterripened seeds.

In the absence of a full-capacity electron transport chain, genes involved in the fermentation process (specifically, pyruvate decarboxylase and alcohol dehydrogenase) are up-regulated together with genes encoding glycolytic enzymes, so that synthesized pyruvate enters the fermentation pathway and is turned into alcohol (Shingaki-Wells et al., 2011; Yu et al., 2014). In agreement, nondormant seeds showed higher expression of genes for isoforms of cytosolic pyruvate kinase (Fig. In3 of this Insight), which catalyzes the final step of glycolysis and plays a critical role in the regulation of glycolysis, and whose activity increases under low oxygen stress in order to compensate for the prevailing depletion in ATP levels (van Dongen et al., 2011).

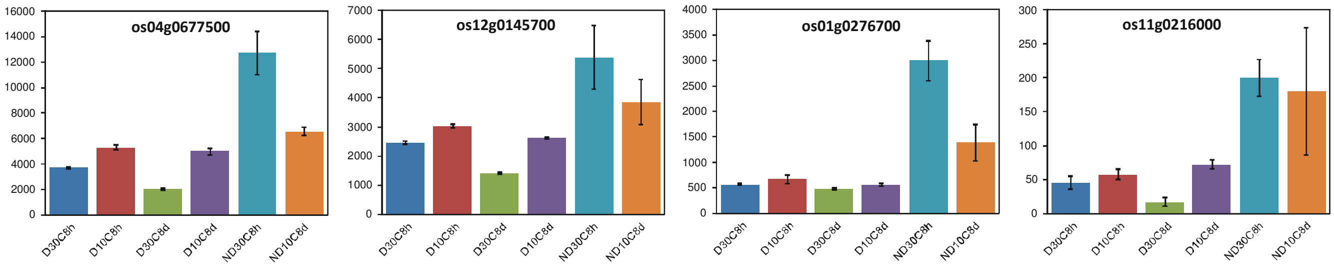

Figure In3. Expression levels, in the tested conditions, of genes for pyruvate kinases Os04g0677500, Os12g0145700, Os01g0276700 and Os11g0216000, which, in cytosolic glycolysis, catalyze the transfer of phosphate from PEP to ADP to produce ATP and pyruvate. Error bars represent standard errors.

As expected, because of this hypoxic-like stress (at 8 h of imbibition) in nondormant seeds, glycolysis deviated from TCA cycle to fermentation, as suggested by the increased expression of the gene for pyruvate decarboxylase (Fig. In4 of this Insight), which catalyzes the first committed reaction of ethanolic fermentation, for which it is the limiting factor (Magneschi and Perata, 2009). This increase occurred even more drastically in consequence of imbibition of seeds, even dormant ones, at suboptimal temperature (10 °C; Fig. In4 of this Insight).

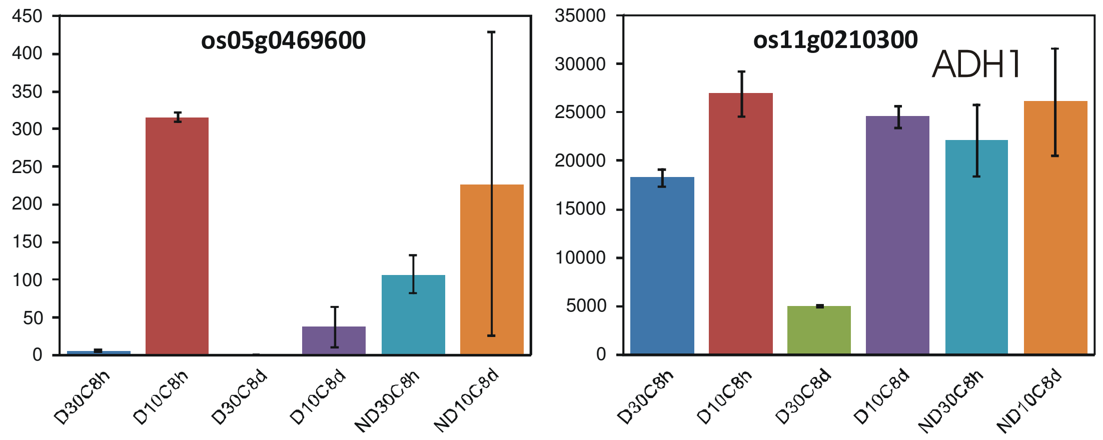

Figure In4. Expression levels, in the tested conditions, of genes for fermentation enzymes pyruvate decarboxylase Os05g0469600 and alcohol dehydrogenase ADH1. Error bars represent standard errors.

Ethanolic fermentation is much less energetically efficient than aerobic respiration and this forces the metabolism to increase the flow of sugars through glycolysis to keep up with the energetic demand of the cell (Magneschi and Perata, 2009). Nonetheless, expression of the alcohol dehydrogenase gene *ADH1* was high in every condition with the exception of 8 d at 30 °C, when it remarkably decreased (Fig. In4 of this Insight), probably because this was the only condition wherein the respiratory chain was no longer impaired. In germinating soybean embryonic axes, pyruvate decarboxylase and alcohol dehydrogenase were found to reach maximum transcriptional levels at 6 h after imbibition, implying that fermentation is indeed more relevant before mitochondria are fully repaired (Bellieny-Rabelo et al., 2016). High expression of alcohol dehydrogenase transcripts after seed imbibition was also reported by Howell et al. (2009) in rice and Sreenivasulu et al. (2008) in barley. Accordingly, alcohol dehydrogenase has been show to increase in the rice embryo of both dormant and nondormant seeds even during aerobic incubation in water (Xu et al., 2016).

Overall, a strong impairing effect of dry-afterripening on energy metabolism could explain the large divergence observed for gene expression in the nondormant seeds imbibed 8 h at 30 °C from all the other samples (Supplementary Fig. S2).

References

Bellieny-Rabelo D., de Oliveira E.A.G., Ribeiro E. da S., Costa E.P., Oliveira A.E.A. and Venancio T.M. (2016). Transcriptome analysis uncovers key regulatory and metabolic aspects of soybean embryonic axes during germination. Sci. Rep. 6:36009.

Botha F.C., Potgieter, G.P. and Botha A.-M. (1992). Respiratory metabolism and gene expression during seed germination. Plant Growth Reg. 11:211-224.

Howell K.A., Millar A.H. and Whelan J. (2006). Ordered assembly of mitochondria during rice germination begins with promitochondrial structures rich in components of the protein import apparatus. Plant Mol. Biol. 60:201-223.

Howell K.A., Narsai R., Carroll A., Ivanova A., Lohse M., Usadel B., Millar A.H. and Whelan J. (2009). Mapping metabolic and transcript temporal switches during germination in rice highlights specific transcription factors and the role of RNA instability in the germination process. Plant Physiol. 149:961-980.

Magneschi L. and Perata P. (2009). Rice germination and seedling growth in the absence of oxygen. Ann. Bot. 103:181-196.

Matilla A.J. and Rodríguez-Gacio M. del C. (2013). Non-symbiotic hemoglobins in the life of seeds. Phytochem. 87:7-15.

Rosental L., Nonogaki H. and Fait A. (2014). Activation and regulation of primary metabolism during seed germination. Seed Sci. Res. 24:1-15.

Shingaki-Wells R.N., Huang S., Taylor N.L., Carroll A.J., Zhou W. and Millar A.H. (2011). Differential molecular responses of rice and wheat coleoptiles to anoxia reveal novel metabolic adaptations in amino acid metabolism for tissue tolerance. Plant Physiol. 156:1706-1724.

Sreenivasulu N., Usadel B., Winter A., Radchuk V., Scholz U., Stein N., Weschke W., Strickert M., Close T.J., Stitt M., Graner A. and Wobus U. (2008). Barley grain maturation and germination: metabolic pathway and regulatory network commonalities and differences highlighted by new MapMan/PageMan profiling tools. Plant Physiol. 146:1738-1758.

van Dongen J.T., Gupta K.J., Ramírez-Aguilar S.J., Araújo W.L., Nunes-Nesi A. and Fernie A.R. (2011). Regulation of respiration in plants: A role for alternative metabolic pathways. J. Plant Physiol. 168:1434-1443.

Weitbrecht K., Müller K. and Leubner-Metzger G. (2011). First off the mark: early seed germination. J. Exp. Bot. 62:3289-3309.

Xu H.-H., Liu S.-J., Song S.-H., Wang R.-X., Wang W.-Q. and Song S.-Q. (2016). Proteomics analysis reveals distinct involvement of embryo and endosperm proteins during seed germination in dormant and non-dormant rice seeds. Plant Physiol. Biochem. 103:219-242.

Yu Y., Guo G., Lv D., Hu Y., Li J., Li X. and Yan Y. (2014). Transcriptome analysis during seed germination of elite Chinese bread wheat cultivar Jimai 20. BMC Plant Biol. 14:20.
