# Supplementary material for: Seed Dormancy Involves a Transcriptional Program That Supports Early Plastid Functionality during Imbibition
Source: Plants (Basel). 2018 Apr 19;7(2):35. doi: 10.3390/plants7020035 (PMC6026906; doi:10.3390/plants7020035)
Supplement: Supplementary file 1 [file plants-07-00035-s001.zip › plants-273866-supplementary-update/Insight into variability between replicates.pdf]

Supplementary material to the article "Seed dormancy involves a transcriptional program that supports early plastid functionality during imbibition" by Gianinetti et al.

Insight into variability between replicates

When expressions of dormant and nondormant seeds were compared after 8 d of incubation (at 10 °C) the number of DEGs plunged (Table 3). This was largely owing to a wider variability between replicates for these samples. In fact, a higher error (between-replicates) variance decreases the power of statistical detection for significant differences and therefore the number of DEGs. This was confirmed by calculating the variances of gene expression intensity between replicates for each expressed gene within every sample, and averaging them across all expressed genes for each sample. Very large differences were observed between samples (Table In1 of this Insight).

Sample	Average between-replicates variance of gene expression
D 30 °C 8 h	309552
D 30 °C 8 d	35923
D 10 °C 8 h	5081322
D 10 °C 8 d	727759
ND 30 °C 8 h	700734
ND 10 °C 8 d	11455709

Table In1. Sample means of between-replicates gene-expression variances. They measure the variability of gene expression between replications. Sample-averaged between-replicates gene-expression variances were compared pair-wise by a nonparametric Mann-Whitney test. Probabilities of equal mean gene-expression replicate variances were very low, always < 0.00001.

A main role of variability between replicates on the number of DEGs is clearly indicated by a strong ($R^2 = 0.837$) negative correlation between the number of detected DEGs and the deviance, pooled over each compared sample pair, obtained from the sample means of between-replicates gene-expression variances (Fig. In1 of this Insight). In fact, in pairwise comparisons, any statistic commonly used to test whether the means are significantly different is inversely proportional to the pooled standard deviation.

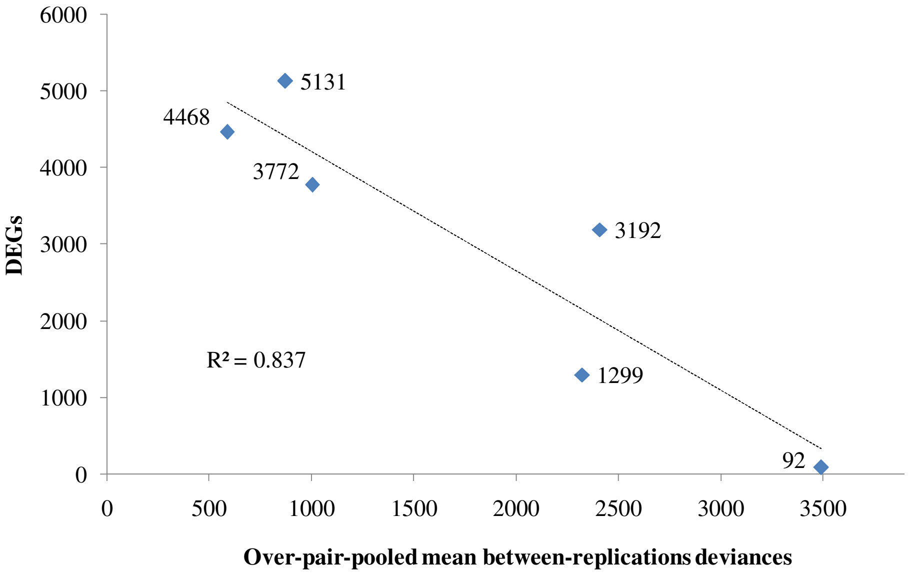

Figure In1. Scatter plot showing a negative correlation between the number of DEGs detected in each pair-wise comparison and the relative deviance, pooled over each pair, and obtained from the sample means of between-replicates gene-expression variances.

The exceptionally high between-replicates gene-expression variance of nondormant seeds incubated 8 d at 10 °C could have a physiological cause. In fact, this treatment aimed to prevent germination of these seeds by using a temperature that is generally not permissive for red rice germination at least within two weeks of incubation (Gianinetti and Cohn, 2008). However, the minimum temperature for germination could slightly vary among seeds, thus that even though no single seed showed external morphological signs of germination (Table 1), they could differ in the advancement of the early germinative metabolism. Analogously, the higher between-replicates gene-expression variances observed for dormant seeds incubated at 10 °C with respect to 30 °C for either 8 h or 8 d, could be due to diversity among seeds in how the low temperature affects their dormancy status (Gianinetti and Cohn, 2008).

Although it is evident that the variability of gene expression between replications was the major determinant of the number of DEGs detected in each pair-wise comparison (Fig. In1 of this Insight), it seems probable that diversity in sample means of between-replicates gene-expression variances (Table In1 of this Insight) can correspond to actual variability in the transcriptional response among seeds. In fact, lower variability among biological replications including several seeds originates from a lower variability among single seeds. If so, it is then clear that dormant seeds incubated at 30 °C for 8 d attained a much greater homogeneity in their transcriptional activity than any other sample. This is a strong support to the purported stabilization of the metabolism in these seeds. Such stabilization was expected, as dormant seeds of our red rice genotype can remain dormant for a long time at 30 °C, and though they ultimately germinate at different times, this appears not to be a metabolic effect (Gianinetti, 2016). However, it was not known how this metabolic stabilization could display at the transcriptional level. It appears to show up in terms of a much greater uniformity in the gene expression levels among seeds.

References

Gianinetti A. (2016). Anomalous germination of dormant dehulled red rice seeds provides a new perspective to study the transition from dormancy to germination and to unravel the role of the caryopsis coat in seed dormancy. Seed Sci. Res. 26:124-138.

Gianinetti A. and Cohn M.A. (2008). Seed dormancy in red rice. XIII: Interaction of dry-afterripening and hydration temperature. Seed Sci. Res. 18:151-159.
