# Supplementary material for: Seed Dormancy Involves a Transcriptional Program That Supports Early Plastid Functionality during Imbibition
Source: Plants (Basel). 2018 Apr 19;7(2):35. doi: 10.3390/plants7020035 (PMC6026906; doi:10.3390/plants7020035)
Supplement: Supplementary file 1 [file plants-07-00035-s001.zip › plants-273866-supplementary-update/Supplemental Figures.pdf]

SUPPLEMENTAL FIGURES

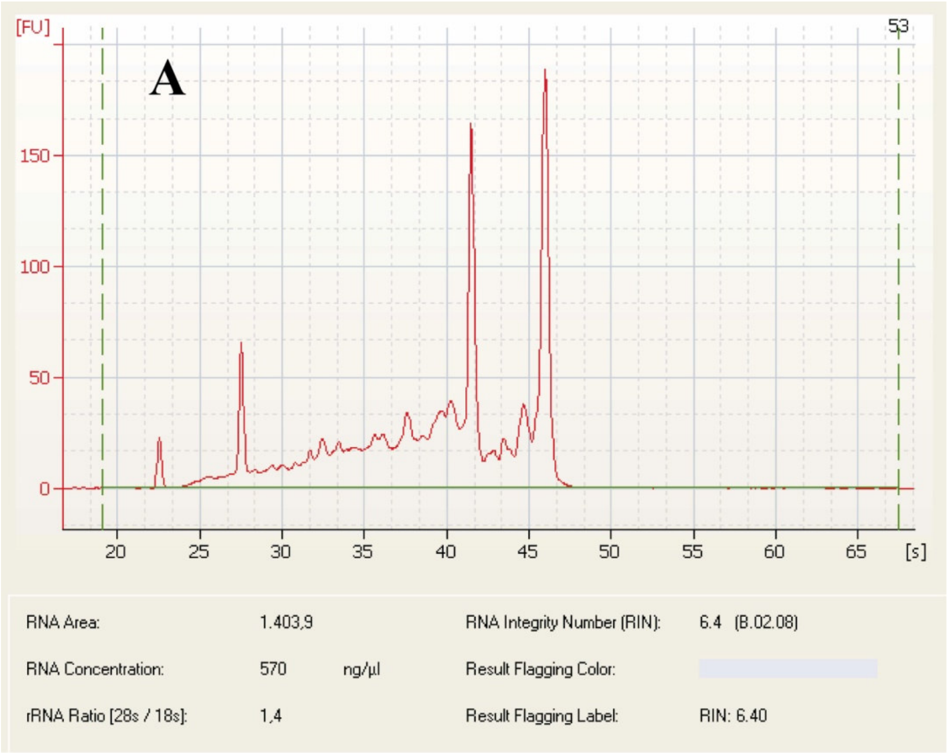

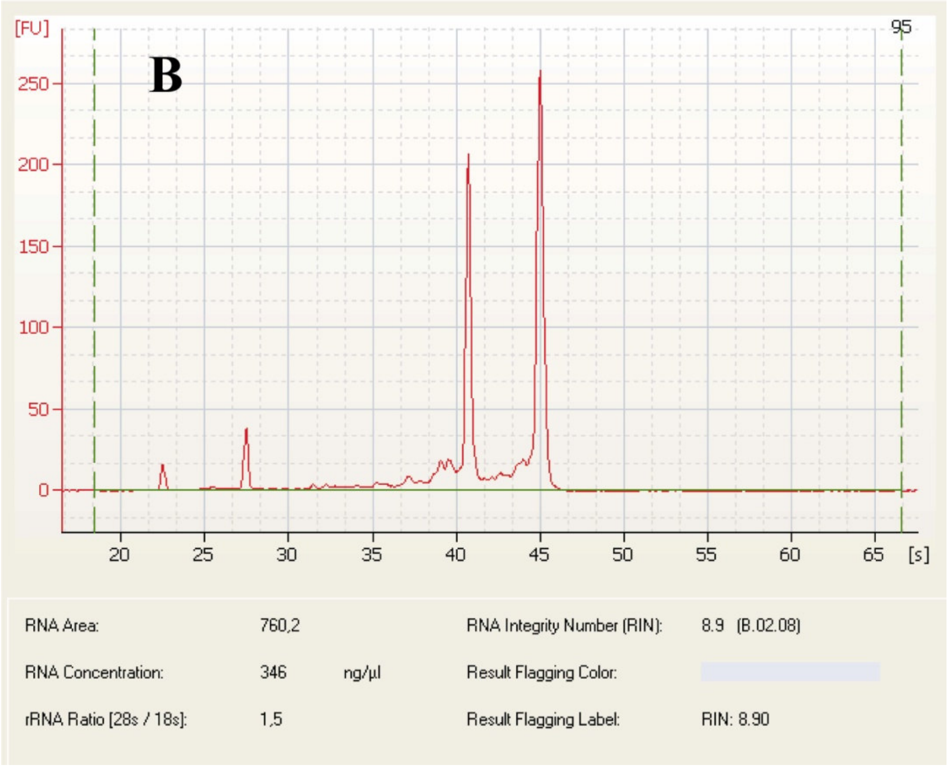

Supplemental Figure S1. Representative Bioanalyzer RNA electropherograms. The two highest peaks corresponds to the 18S and 25S ribosomal RNA species, respectively. (A) Typical electropherogram for ND seeds; (B) typical electropherogram for D seeds.

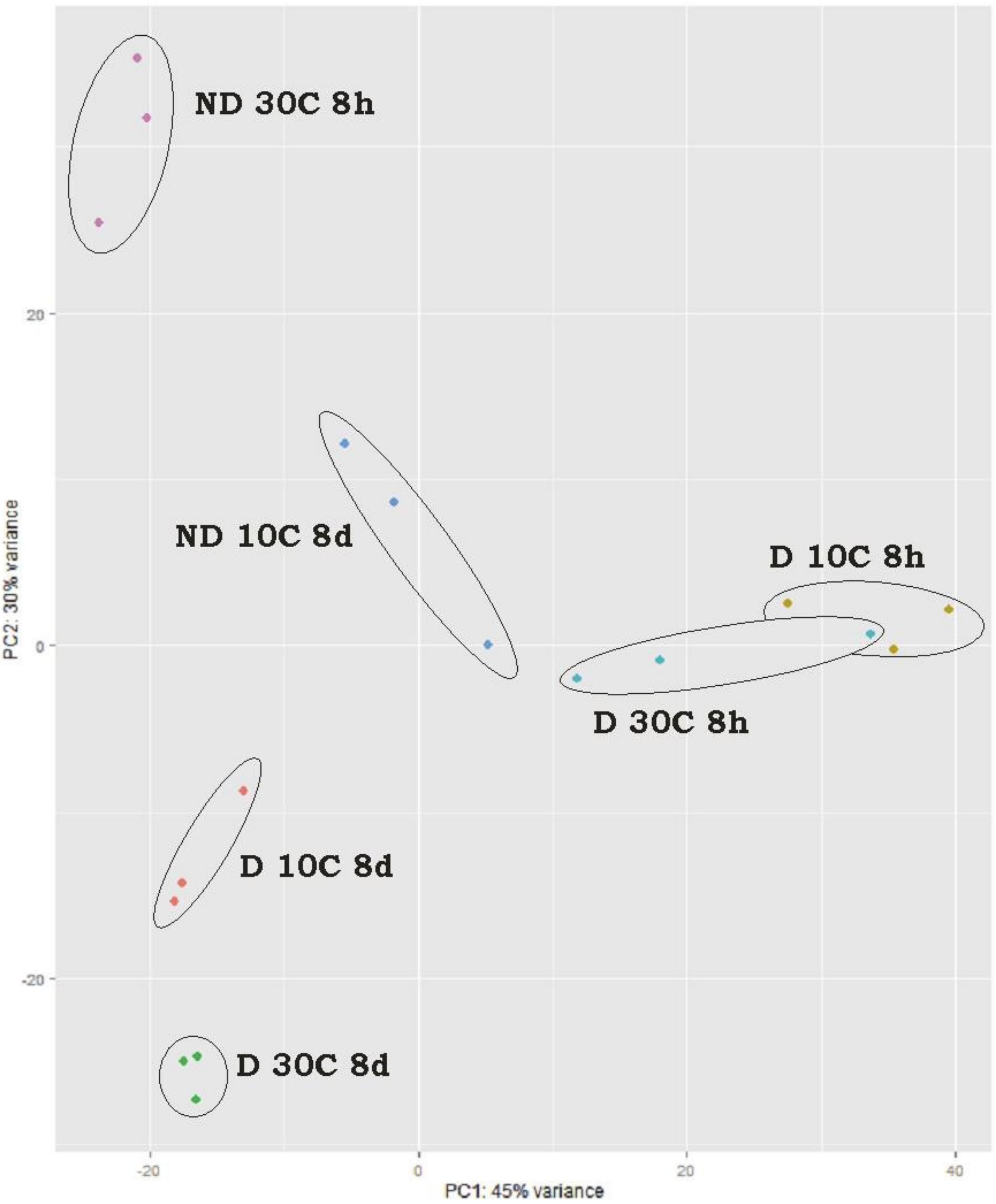

Supplemental Figure S2. Principal Component Analysis showing how the overall variability observed for gene expression distributes over the first two Principal Components, which together described 75% of the total variance, across experimental conditions and their replicates. To avoid both heteroskedasticity and that the result either depended only on the few most strongly expressed genes or it were dominated by the many genes with the very lowest counts, data were subjected to regularized-logarithm transformation (rlog).

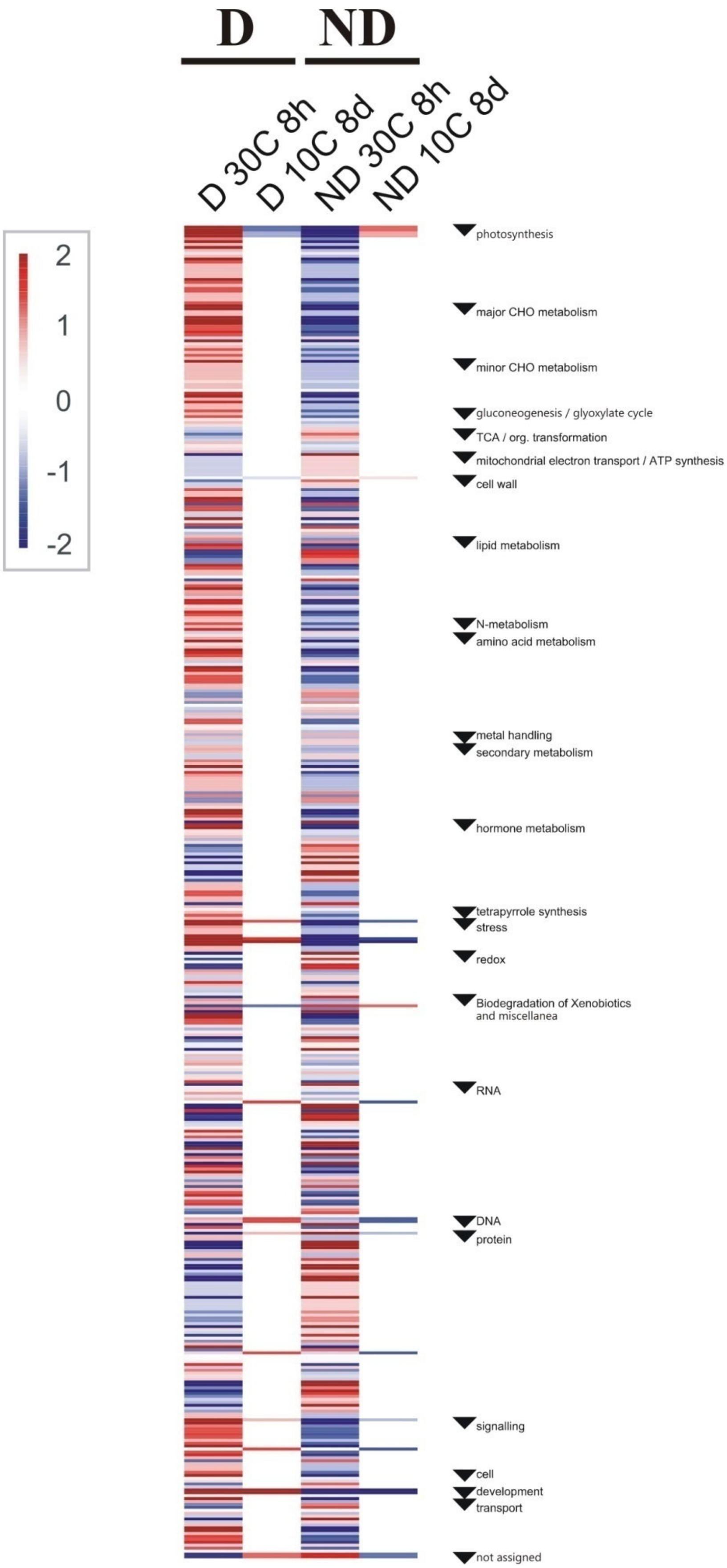

Supplemental Figure S3. Comparison for differential gene expression of PageMan BINs (groups of functionally similar genes) of D and ND seeds incubated for either 8h at 30 °C or 8d at 10 °C (DEG sets for D 30°C 8h *vs* ND 30°C 8h and D 10°C 8d *vs* ND 10°C 8d were compared). For graphical visualization, probabilities of over/under-representation are presented as z-scores. Thus, colours intensities represent more/less items than expected by chance in each given BIN, with increasing red intensity showing over-representation and blue showing under-representation (colour scale on the left).

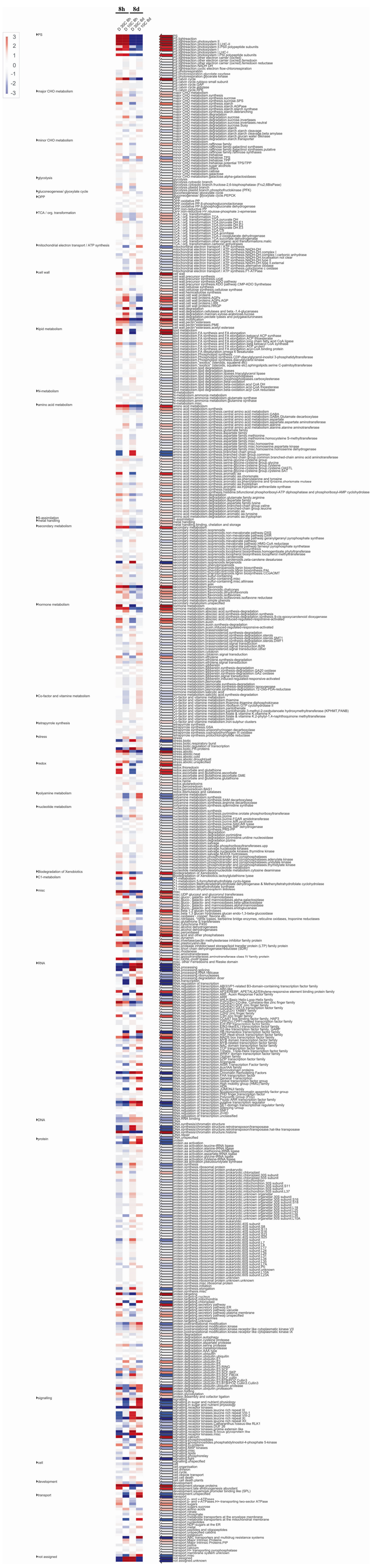

Supplemental Figure S4. Comparison for differential gene expression of PageMan BINs (groups of functionally similar genes) of D seeds incubated for either 8h or 8d at 30 °C or 10 °C (DEG sets for D 30°C 8d *vs* D 30°C 8h and D 10°C 8d *vs* D 10°C 8h were compared). For graphical visualization, probabilities of over/under-representation are presented as z-scores. Thus, colours intensities represent more/less items than expected by chance in each given BIN, with increasing red intensity showing over-representation and blue showing under-representation (colour scale on the upper left).

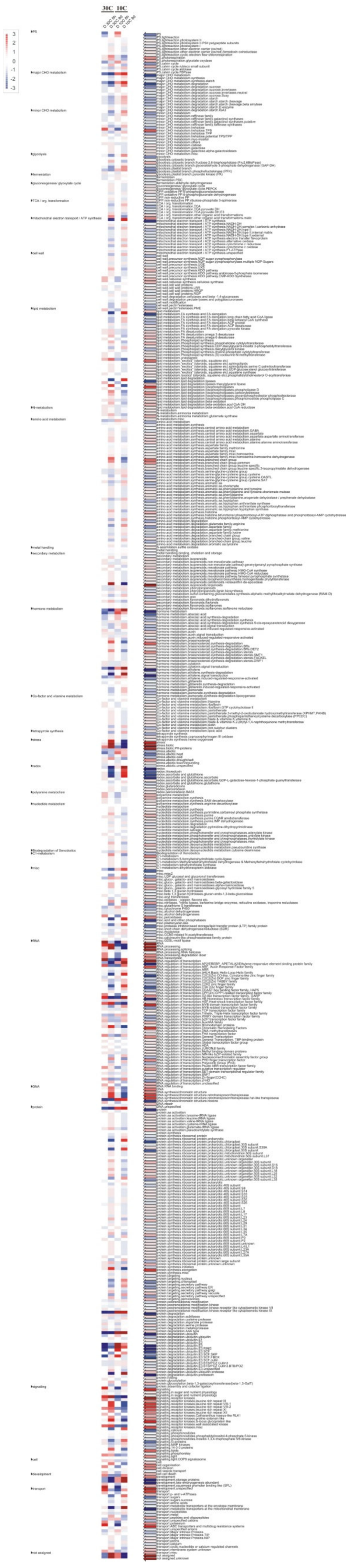

Supplemental Figure S5. Comparison for differential gene expression of PageMan BINs (groups of functionally similar genes) of D and ND seeds incubated at either 30 °C or 10 °C for 8h or 8d (DEG sets for D 30°C 8h *vs* D 10°C 8h and D 30°C 8d *vs* D 10°C 8d were compared). For graphical visualization, probabilities of over/under-representation are presented as z-scores. Thus, colours intensities represent more/less items than expected by chance in each given BIN, with increasing red intensity showing over-representation and blue showing under-representation (colour scale on the upper left).

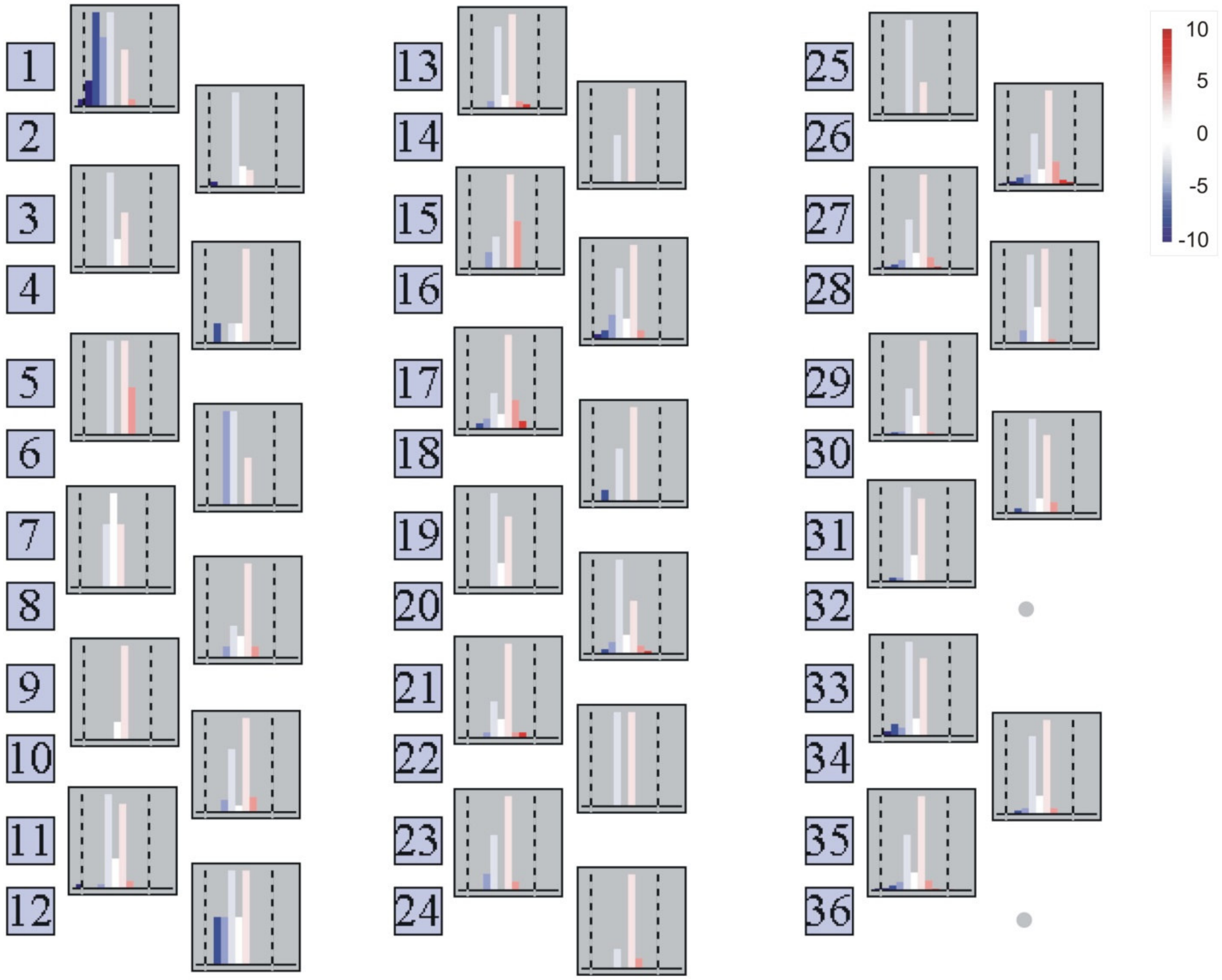

Supplemental Figure S6. Comparison between gene expression in D and ND seeds incubated in water for 8h at 30 °C. In MapMan Overview, DEGs are grouped in functional BINs: 1- Photosynthesis, 2- Major carbohydrates, 3- Minor carbohydrates, 4- Glycolysis, 5- Fermentation, 6- Gluconeogenesis/glyoxylate cycle, 7- OPP cycle, 8- TCA/organic acid transformations, 9- Mitochondrial electron transport/ATP synthesis, 10- Cell wall, 11- Lipid metabolism, 12- Nitrogen assimilation, 13- Amino acid metabolism, 14- S-assimilation, 15- Metal handling, 16- Secondary metabolism, 17- Hormones, 18- Cofactor and vitamin synthesis, 19- Tetrapyrrole synthesis, 20- Stress, 21- Redox, 22- Polyamine synthesis, 23- Nucleotide metabolism, 24- Biodegradation of Xenobiotics, 25- C1-metabolism, 26- Miscellaneous enzyme families, 27- RNA, 28- DNA, 29- Protein, 30- Signalling, 31- Cell, 32- microRNA, 33- Development, 34- Transport, 35- Not assigned, 36- Biodegradation of xenobiotics. Gray dots represent empty BINs (no DEGs were detected in this comparison). For each BIN, the distribution of DEGs according to their relative expression level in this contrast is shown: histograms represent the number of DEGs and their colours indicate the relative expression levels in terms of log_2FC (scale on the right upper corner), with red showing relative higher expression in ND seeds and blue in D ones. Dashed lines show the limits fixed for the colour scale.

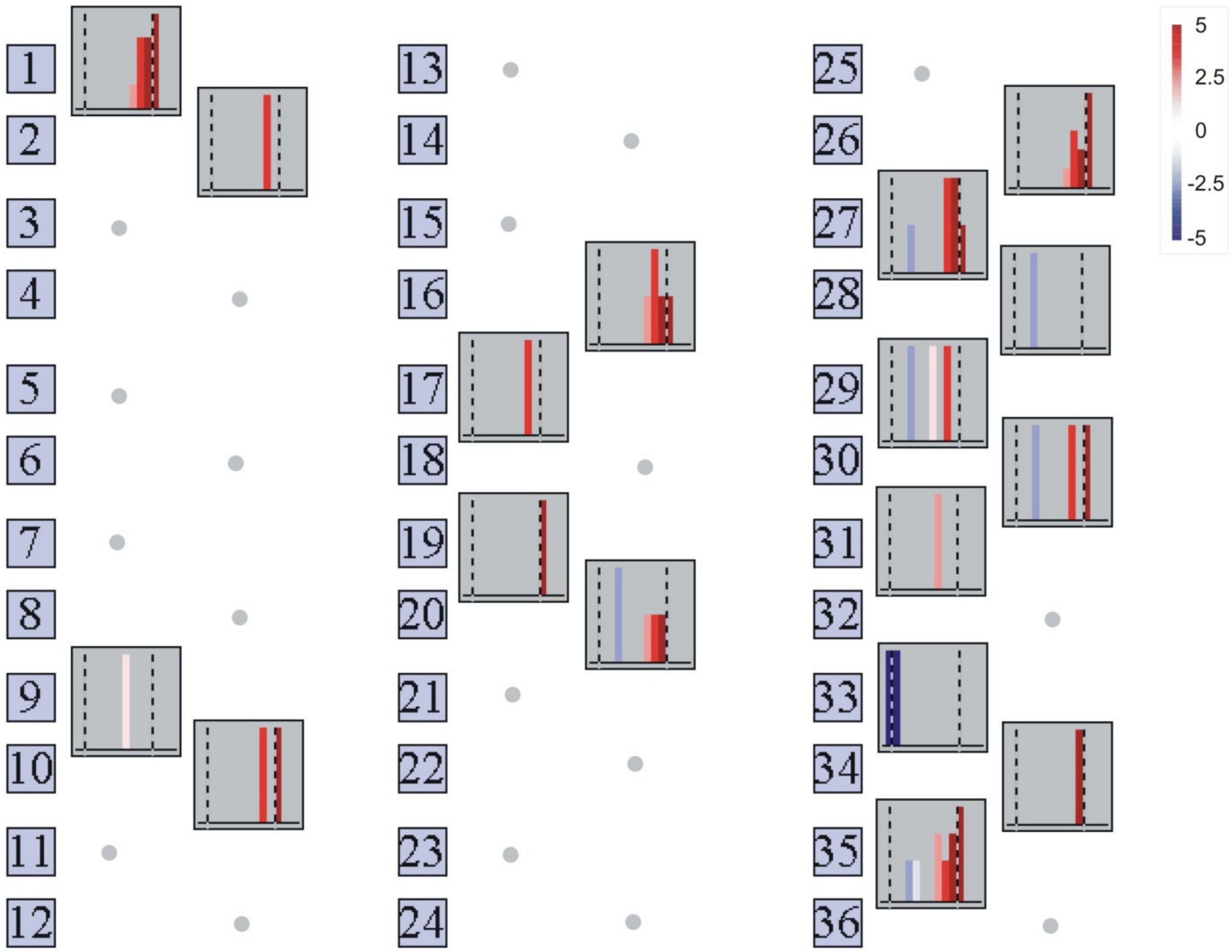

Supplemental Figure S7. Comparison between gene expression in D seeds incubated in water for either eight hours (8h) or eight days (8d) at 10 °C. MapMan Metabolism overview: single DEGs are represented by elementary squares grouped in functional BINs and their colour indicates the relative expression level in this contrast (in terms of log_2FC, scale on the right upper corner), with red showing relative higher expression after 8d and blue after 8h.

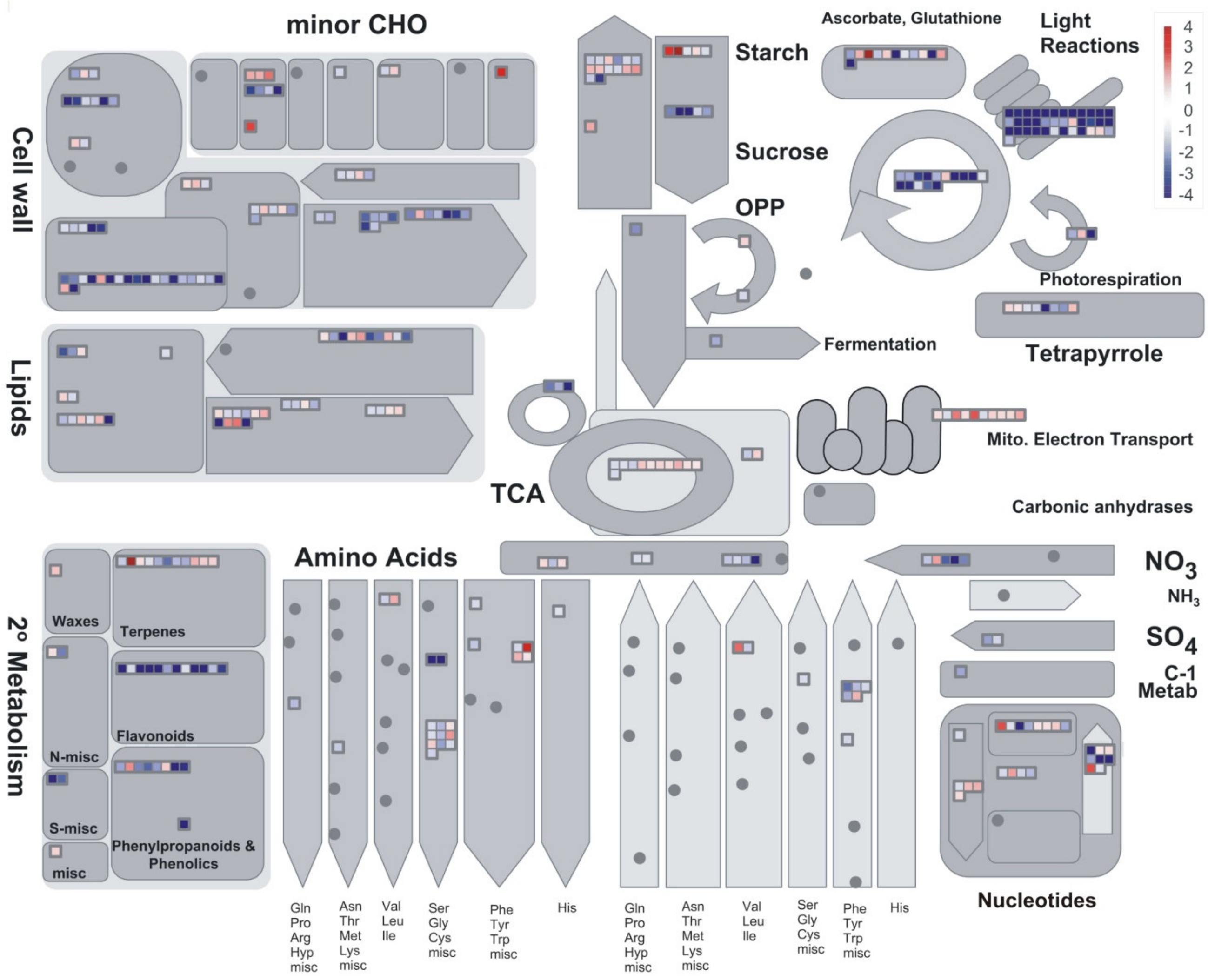

Supplemental Figure S8. Comparison between gene expression in D seeds incubated in water for eight days (8d) at either 10 °C or 30 °C. MapMan Metabolism overview: single DEGs are represented by elementary squares grouped in functional BINs and their colour indicates the relative expression level in this contrast (in terms of $\log_2$FC, scale on the right upper corner), with red showing relative higher expression at 10 °C and blue at 30 °C.

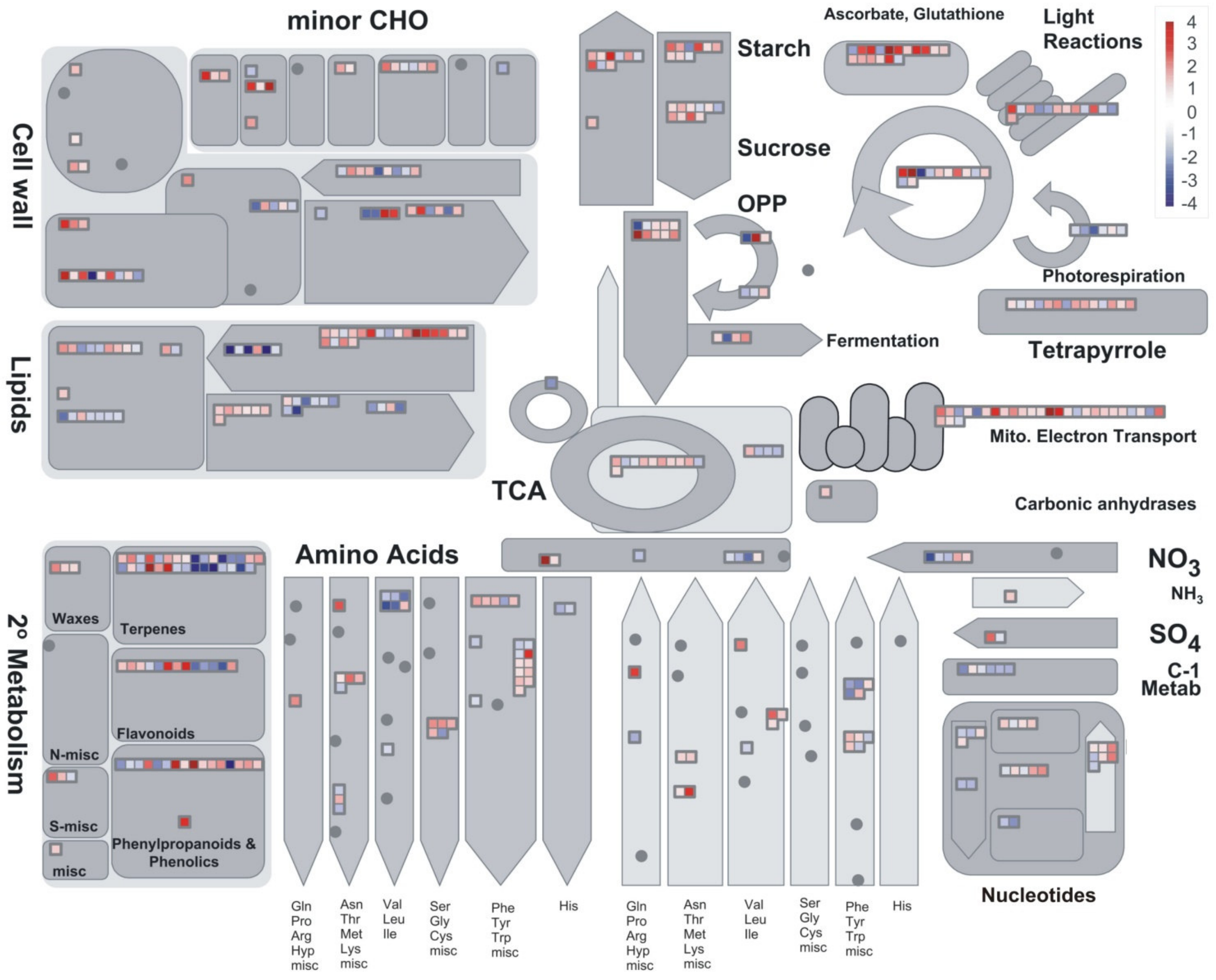

Supplemental Figure S9. Comparison between gene expression in D and ND seeds incubated in water for eight days (8d) at 10 °C. In MapMan Overview, DEGs are grouped in functional BINs: 1- Photosynthesis, 2- Major carbohydrates, 3- Minor carbohydrates, 4- Glycolysis, 5- Fermentation, 6- Gluconeogenesis/glyoxylate cycle, 7- OPP cycle, 8- TCA/organic acid transformations, 9- Mitochondrial electron transport/ATP synthesis, 10- Cell wall, 11- Lipid metabolism, 12- Nitrogen assimilation, 13- Amino acid metabolism, 14- S-assimilation, 15- Metal handling, 16- Secondary metabolism, 17- Hormones, 18- Cofactor and vitamin synthesis, 19- Tetrapyrrole synthesis, 20- Stress, 21- Redox, 22- Polyamine synthesis, 23- Nucleotide metabolism, 24- Biodegradation of xenobiotics, 25- C1-metabolism, 26- Miscellaneous enzyme families, 27- RNA, 28- DNA, 29- Protein, 30- Signalling, 31- Cell, 32- microRNA, 33- Development, 34- Transport, 35- Not assigned, 36- Biodegradation of xenobiotics. Gray dots represent empty BINs (no DEGs were detected in this comparison). For each BIN, the distribution of DEGs according to their relative expression level in this contrast is shown: histograms represent the number of DEGs and their colours indicate the relative expression levels in terms of log_2FC (scale on the right upper corner), with red showing relative higher expression in ND seeds and blue in D ones. Dashed lines show the limits fixed for the colour scale.

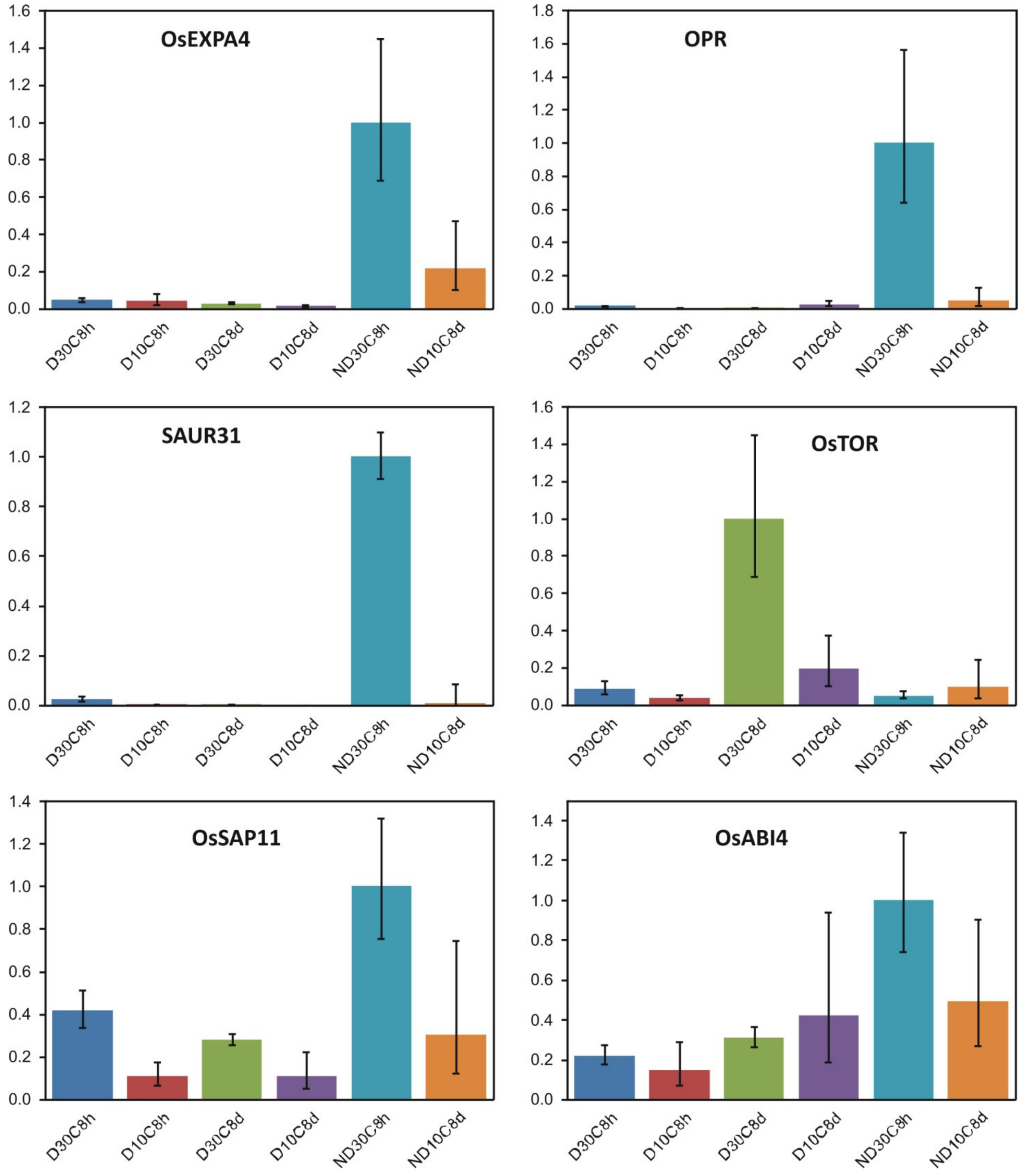

Supplemental Figure S10. Quantitative RT-PCR analysis for representative genes. For every gene, the average C_T (cycle threshold) value of each tested condition was normalized to the corresponding C_T value of the internal control gene, *Edf*, and the relative mRNA level averages across the different tested conditions were further normalized to the highest average value. For every data mean, the C_T value was the average of C_T values obtained from the three biological replicates, each with duplicate RT-qPCR analyses (error bars indicate standard errors between the three biological replicates).

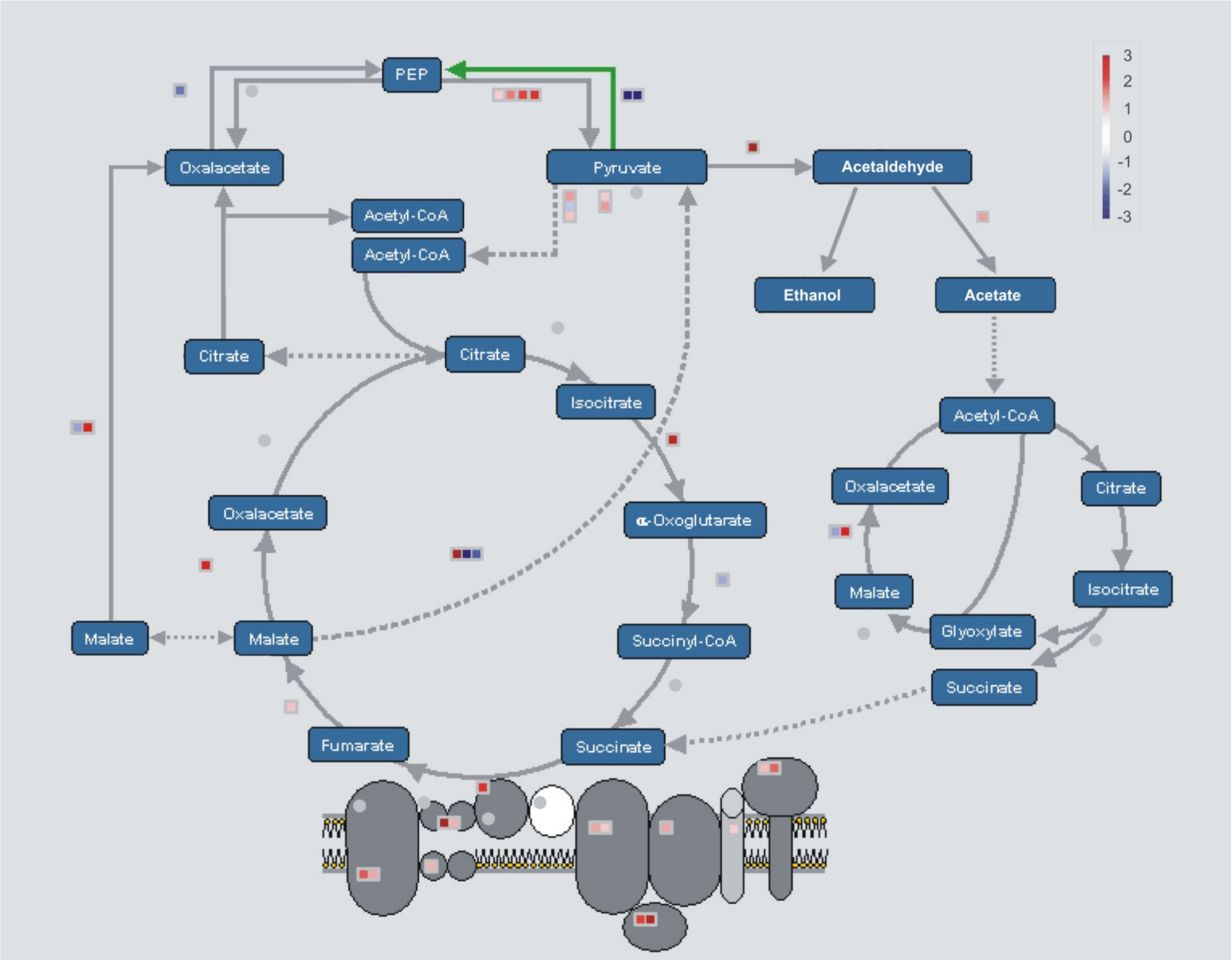

Supplemental Figure S11. Comparison between gene expression in D and ND seeds incubated in water for 8h at 30 °C: MapMan overview of glyoxylate and TCA cycles plus respiratory chain and plastidic (green arrow) gluconeogenic reaction. Single DEGs are represented by elementary squares grouped in functional BINs and their colour indicates the relative expression level in this contrast (in terms of log_2FC, scale on the right), with red showing relative higher expression in ND seeds and blue in D ones.

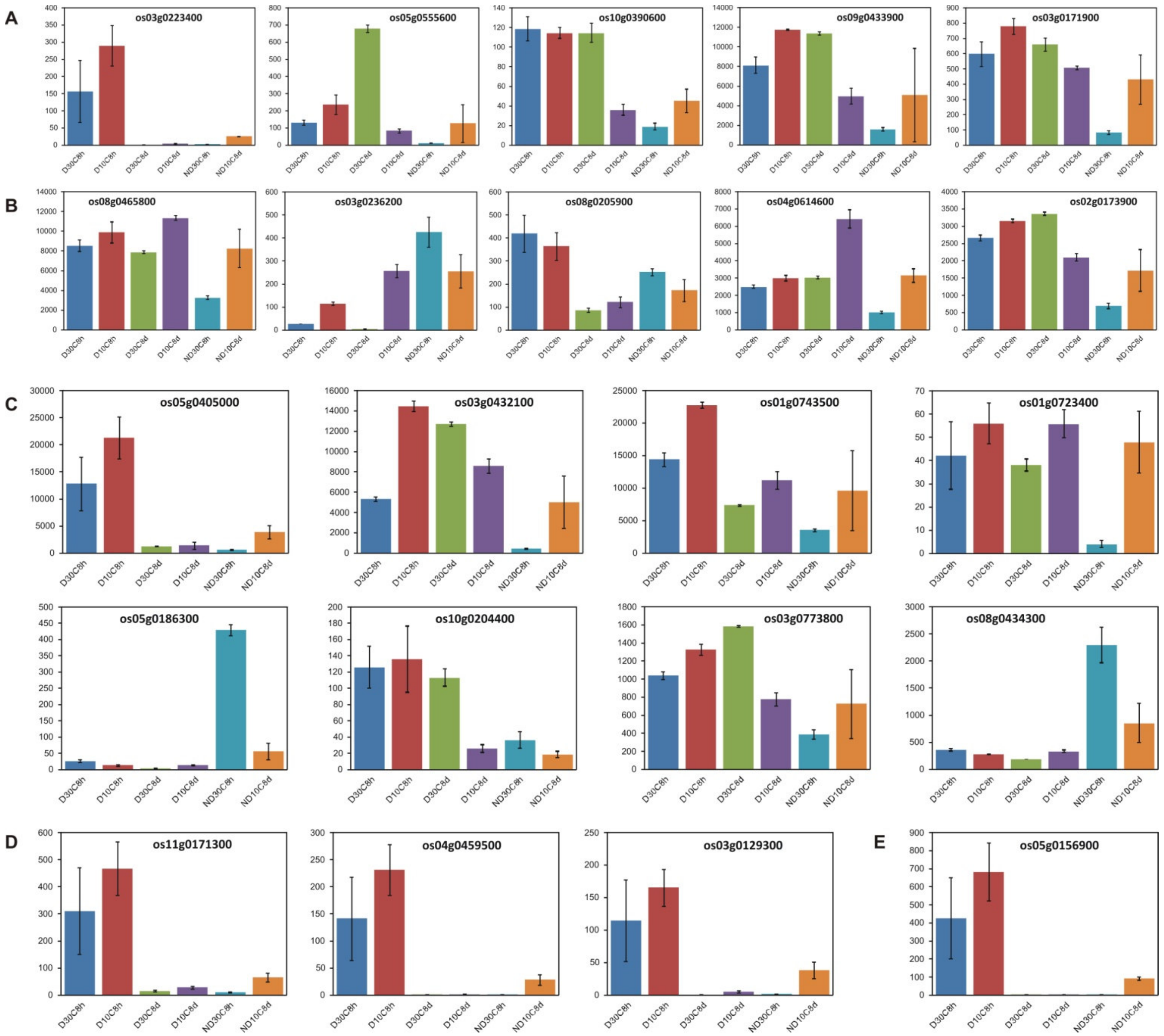

Supplemental Figure S12. Gene expression levels for the tested conditions (error bars represent standard errors). (A) Nitrogen metabolism: glutamine synthase Os03g0223400, glutamate synthase Os05g0555600, alanine aminotransferases Os10g0390600 and Os09g0433900, alanine:glyoxylate aminotransferase Os03g0171900. (B) Enzymes involved in GABA metabolism: glutamate decarboxylases (cytosolic) Os08g0465800 and Os03g0236200 produce GABA; GABA:pyruvate transaminase Os08g0205900 (mitochondrial) transfers an amino group from GABA to pyruvate or, to the reverse, from alanine to succinic semialdehyde; GABA transaminase Os04g0614600 (mitochondrial) performs the catabolic deamination of GABA to succinic semialdehyde by transferring the amino group to 2-oxoglutarate coming from the TCA cycle, thereby forming glutamate and starting the GABA shunt (or, alternatively, 2-oxoglutarate can be produced from glutamate by alanine aminotransferase); succinate-semialdehyde dehydrogenase Os02g0173900 (mitochondrial) produces succinate that re-enters the TCA cycle closing the GABA shunt. (C) Enzymes involved in gluconeogenesis: pyruvate phosphate dikinases Os05g0405000 (plastidic) and Os03g0432100

(cytosolic) phosphorylate pyruvate to phosphoenolpyruvate; NADPH malic enzymes Os01g0743500 (cytosolic), Os01g0723400 (plastidic) and Os05g0186300 decarboxylate malate to pyruvate; phosphoenolpyruvate carboxykinase Os10g0204400 decarboxylates oxaloacetate to phosphoenolpyruvate (in the cytosol); malate dehydrogenases Os03g0773800 and Os08g0434300 catalyze the interconversion between malate and oxaloacetate. (D) Enzymes involved in the plastid branch of glycolysis: fructose-bisphosphate aldolase Os11g0171300 and NADP-dependent glyceraldehyde-3-phosphate dehydrogenase subunits A (Os04g0459500) and B (Os03g0129300). (E) H^+-translocating pyrophosphatase membrane proton pump Os05g0156900.

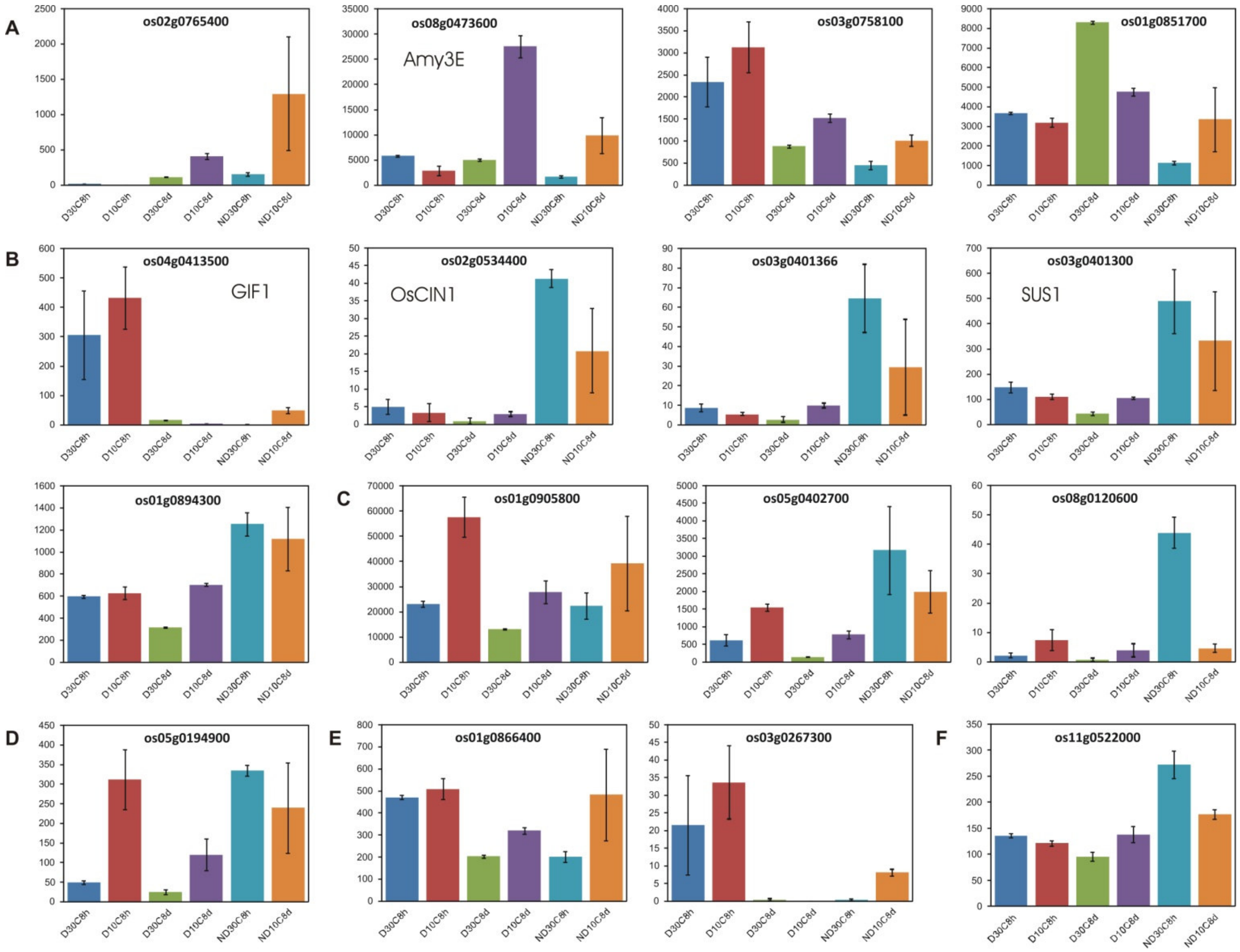

Supplemental Figure S13. Gene expression levels for the tested conditions (error bars represent standard errors). (A) Enzymes involved in the utilization of endosperm polysaccharide reservoirs: α-amylases Os02g0765400 and Amy3E break down starch, starch phosphorylases Os03g0758100 and Os01g0851700 catalyze the reversible conversion of starch and inorganic phosphate to glucose-1-phosphate. (B) Invertases GIF1 and OsCIN1 hydrolyze sucrose into glucose and fructose, sucrose synthases Os03g0401366 and SUS1 catalyze the UDP-dependent cleavage of sucrose into UDP-glucose and fructose, fructokinase Os01g0894300 catalyzes the phosphorylation of fructose to fructose-6-phosphate. (C) Fructose-bisphosphate aldolases Os01g0905800, Os05g0402700 and Os08g0120600 split fructose-1,6-bisphosphate into dihydroxyacetone phosphate and glyceraldehyde 3-phosphate. (D) Pyrophosphate-dependent phosphofructokinase Os05g0194900 catalyzes the reversible interconversion of fructose-6-phosphate and fructose-1,6-bisphosphate using inorganic pyrophosphate as the phosphoryl donor. (E) Fructose-1,6-bisphosphatases Os01g0866400 (cytosolic) and Os03g0267300 (plastidic) convert fructose-1,6-bisphosphate to fructose-6-phosphate in gluconeogenesis. (F) Fructose-2,6-bisphosphatase Os11g0522000 (not a DEG, included for completeness) phosphorylates fructose-6-phosphate to fructose-2,6-bisphosphate.

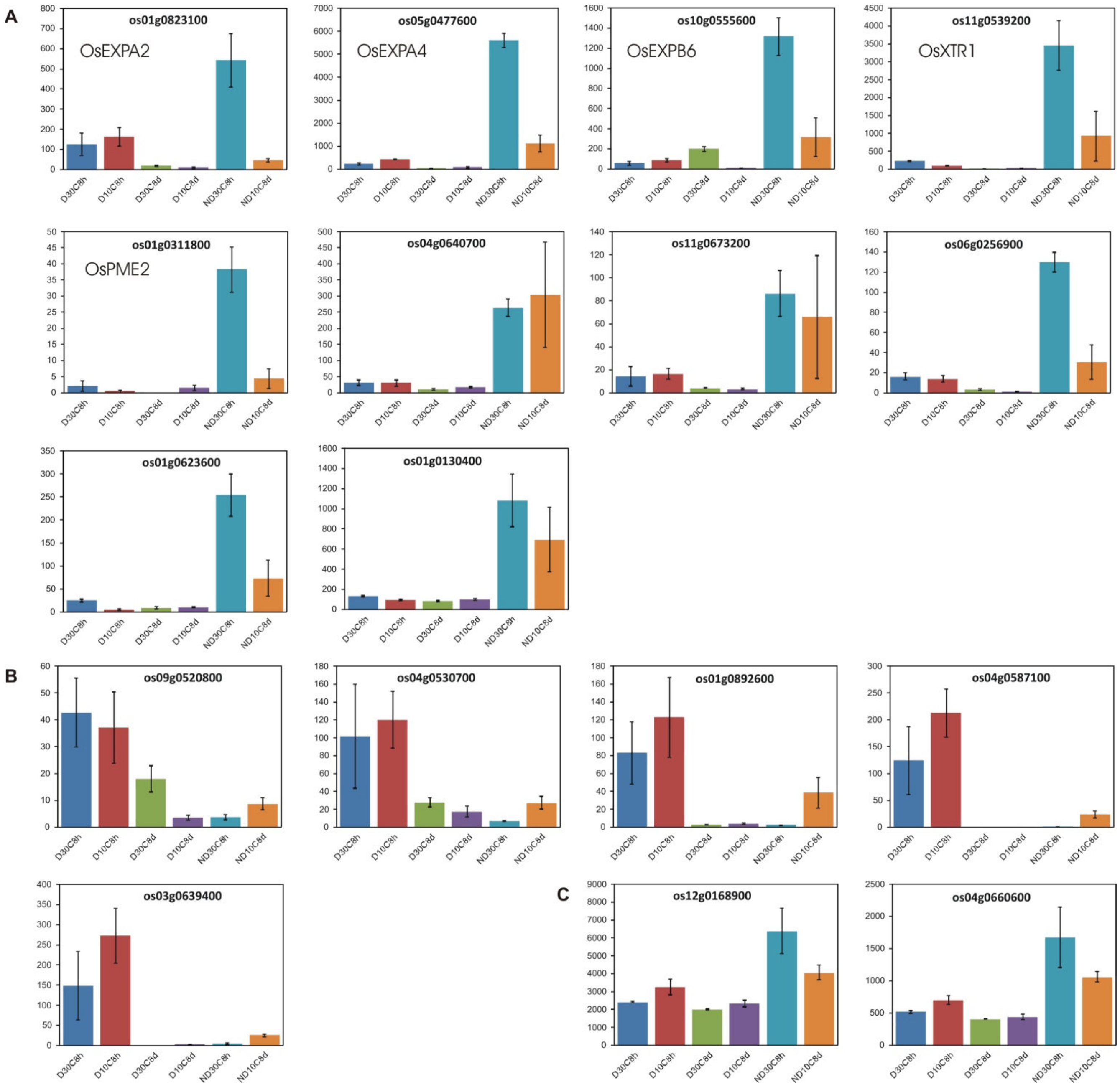

Supplemental Figure S14. Gene expression levels for the tested conditions (error bars represent standard errors). (A) Cell-wall modifying enzymes up-regulated in ND seeds: expansins OsEXPA2, OsEXPA4 and OsEXPB6, xyloglucan endotransglycosylases OsXTR1, pectin methylesterase OsPME2, β-xylosidases Os04g0640700 and Os11g0673200, endo-1,4-β-glucanase Os06g0256900, polygalacturonase Os01g0623600, and α-xylosidase Os01g0130400. (B) Cell-wall modifying enzymes up-regulated in D seeds: glycosyl hydrolases Os09g0520800 and Os04g0530700, pectin acetylesterase Os01g0892600, and invertase/pectin methylesterase inhibitors Os04g0587100 and Os03g0639400. (C) proton-transporting ATPases Os12g0168900 and Os04g0660600.

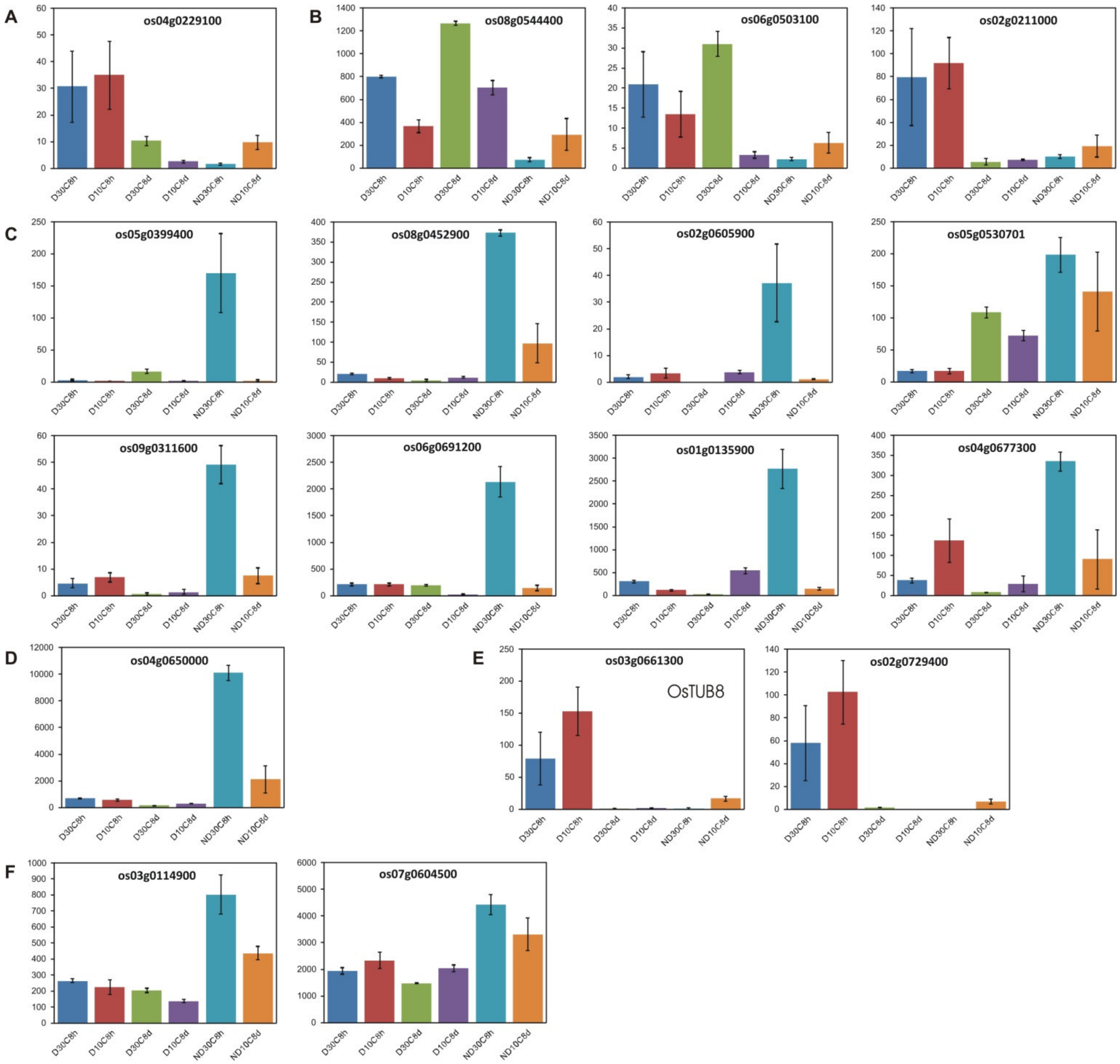

Supplemental Figure S15. Gene expression levels for the tested conditions (error bars represent standard errors). (A) Cinnamyl alcohol dehydrogenase Os04g0229100 is involved in the synthesis of phenylpropanoids. (B) ATP-binding cassette (ABC) transporters Os08g0544400, Os06g0503100 and Os02g0211000 are involved in the transport of flavonoids. (C) Stress related-transcripts, mostly linked to biotic stresses. (D) Cysteine protease Os04g0650000 is involved in defence from biotic stresses. (E) Genes for β-tubulin OsTUB8, which is involved in the building of microtubules, and rhodanese-like domain containing protein Os02g0729400, which acts as extracellular calcium sensing receptor, may be involved in the organization of plastids. (F) Mitochondrial import inner membrane translocase subunits Os03g0114900 and Os07g0604500.

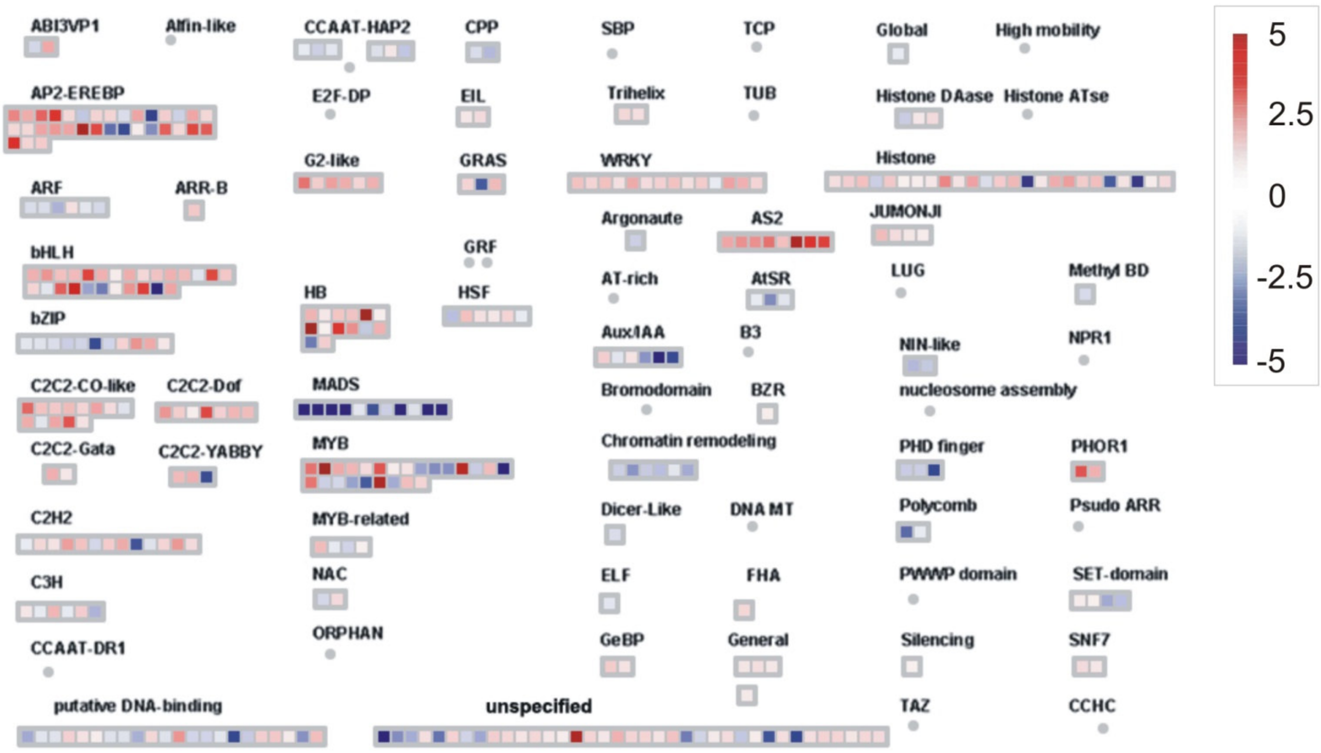

Supplemental Figure S16. Comparison between gene expression in D and ND seeds incubated in water for 8h at 30 °C: MapMan overview of transcription. Single DEGs are represented by elementary squares grouped in BINs corresponding to the diverse families of transcription factors, and their colour indicates the relative expression level in this contrast (in terms of log_2FC, scale on the right), with red showing relative higher expression in ND seeds and blue in D ones.

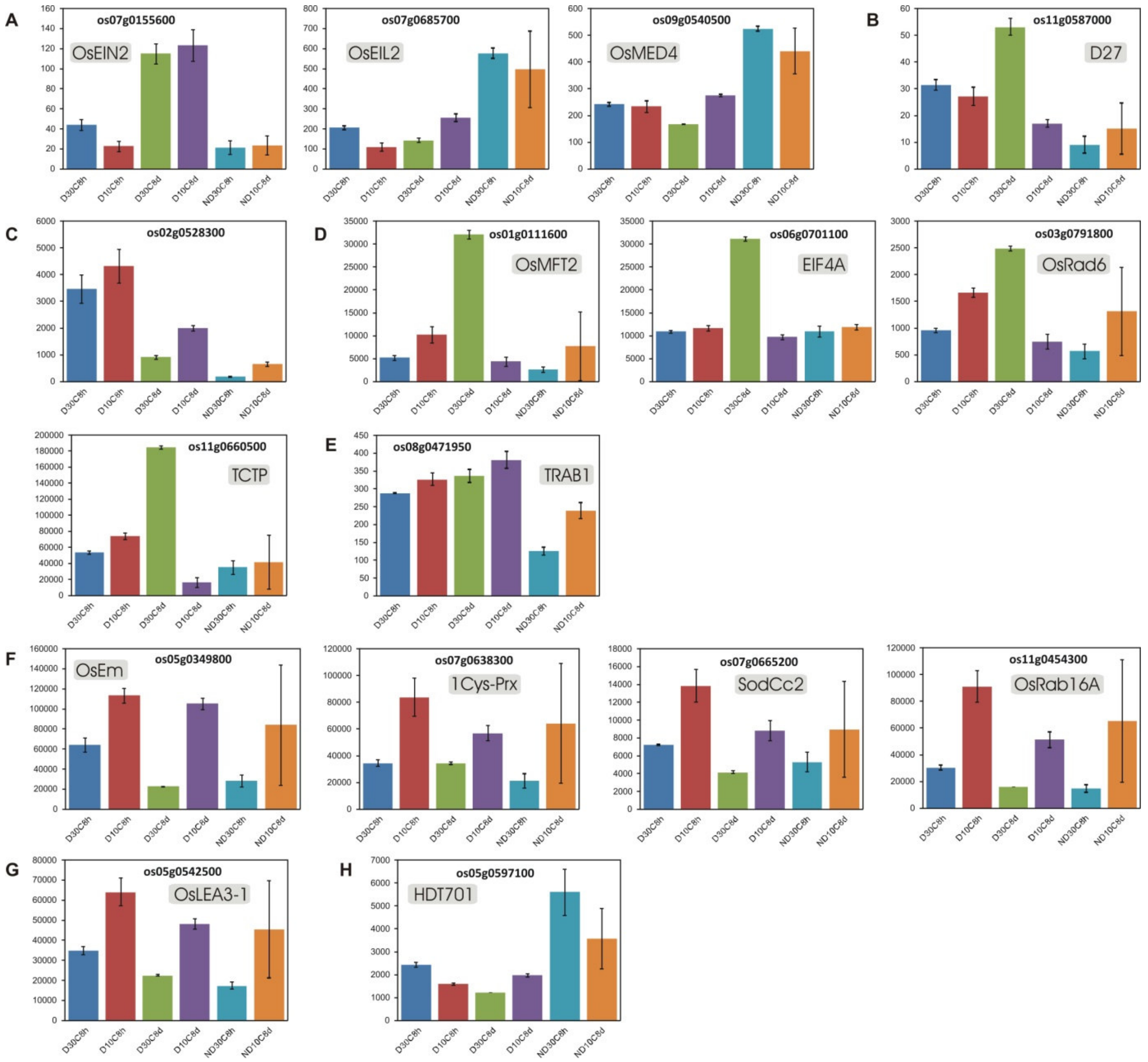

Supplemental Figure S17. Gene expression levels for the tested conditions (error bars represent standard errors). (A) Regulators of the ethylene signalling pathway OsEIN2, OsEIL2 and MED4. (B) 9-cis/all-trans-β-carotene isomerase D27. (C) Putative ABA responsive protein Os02g0528300. (D) OsMFT2, a putative homolog of arabidopsis MOTHER OF FT AND TFL1; EIF4A, a subunit of the eukaryotic initiation factor 4A that acts as an ATP-dependent RNA helicase unwinding mRNA secondary structures; OsRad6, a ubiquitin-conjugating protein E2; TCTP, a so-called translationally-controlled tumor protein, which acts as a regulator of TOR. (E) TRAB1, an ABF bZIP factor that mediates ABA signals to activate transcription. (F) ABA-responsive genes *OsEm*, *1Cys-Prx*, *SodCc2* and *OsRab16A*. (G) Late embryogenesis abundant protein OsLEA3-1, a putative dehydrin. (H) Gene Os05g0597100 for the HD2-type histone deacetylase HDT701.

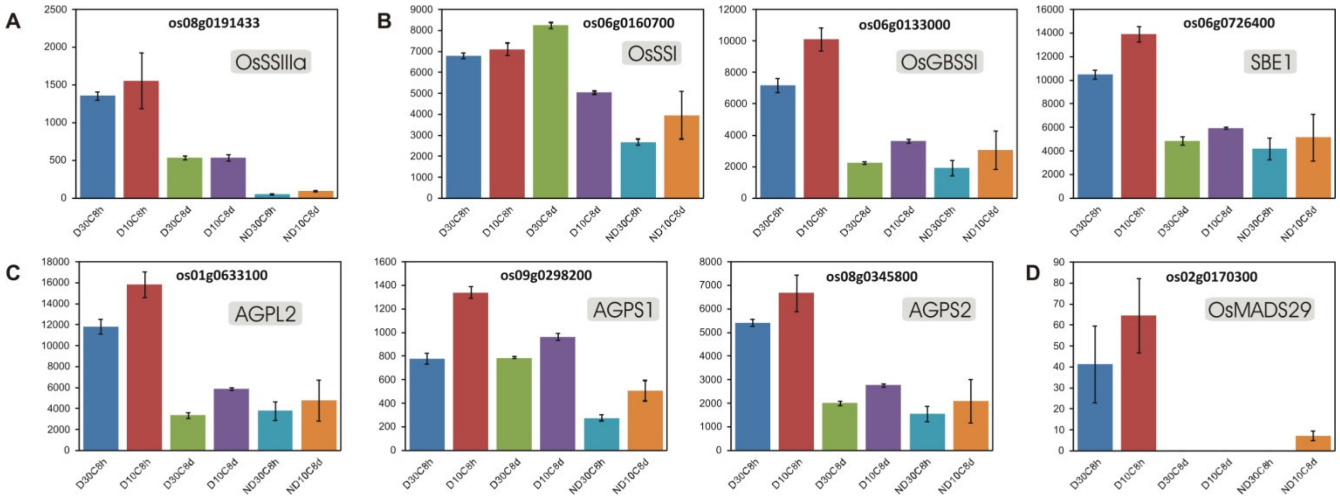

Supplemental Figure S18. Gene expression levels for the tested conditions (error bars represent standard errors). (A) Gene *OsSSIIIa* for soluble starch synthase IIIa. (B) Starch synthase OsSSI, granule-bound starch synthase OsGBSSI, starch branching enzyme SBE1. (C) ADP-glucose pyrophosphorylase subunits: large subunit 2 (AGPL2), small subunit 1 (AGPS1), small subunit 2 (AGPS2). (D) MADS-box transcription factor 29 (OsMADS29).

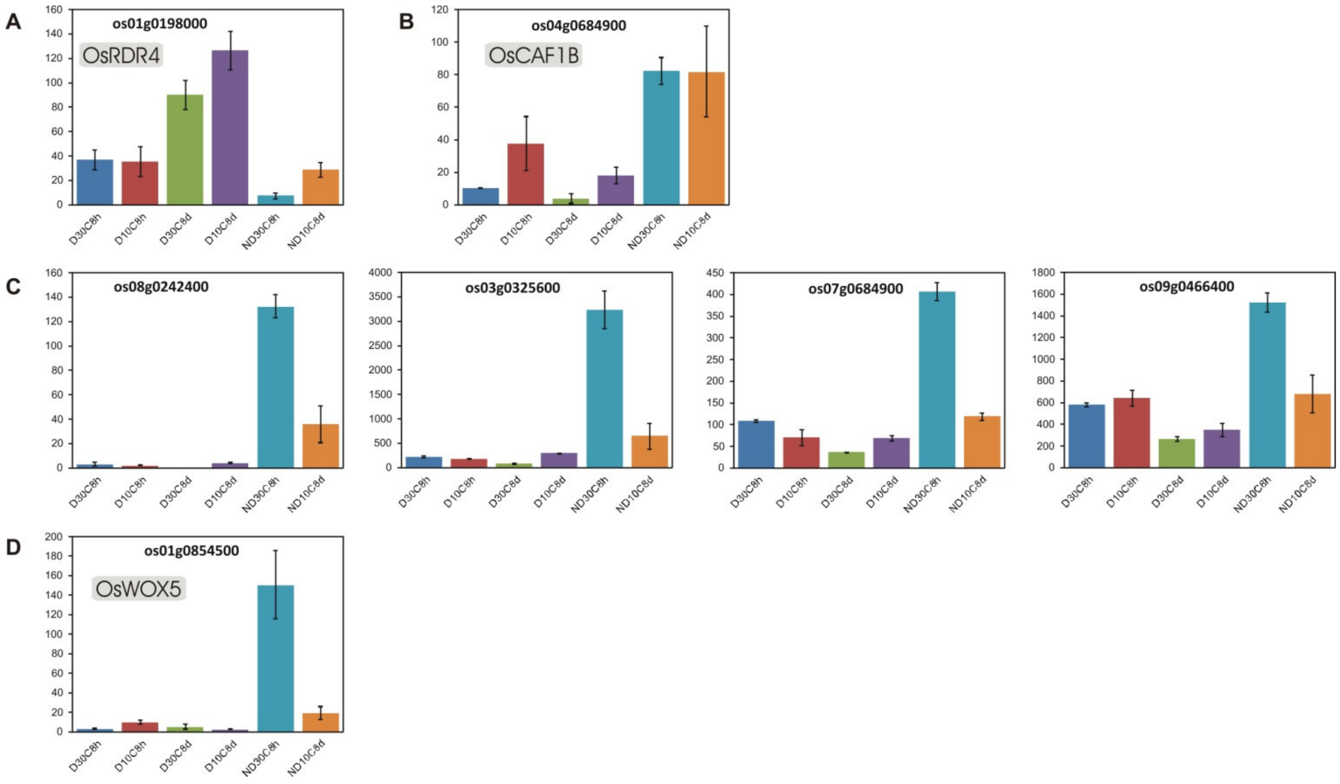

Supplemental Figure S19. Gene expression levels for the tested conditions (error bars represent standard errors). (A) RNA-dependent RNA polymerase OsRDR4. (B) Putative CCR4-NOT transcription complex subunit OsCAF1B is involved in regulating mRNA deadenylation and degradation. (C) Germination-specific transcription factors: WUSCHEL-related homeobox (HB) transcription factors Os08g0242400, Os03g0325600, and Os07g0684900, and zinc finger homeodomain transcription factor Os09g0466400. (D) WUSCHEL-type homeobox Os01g0854500.

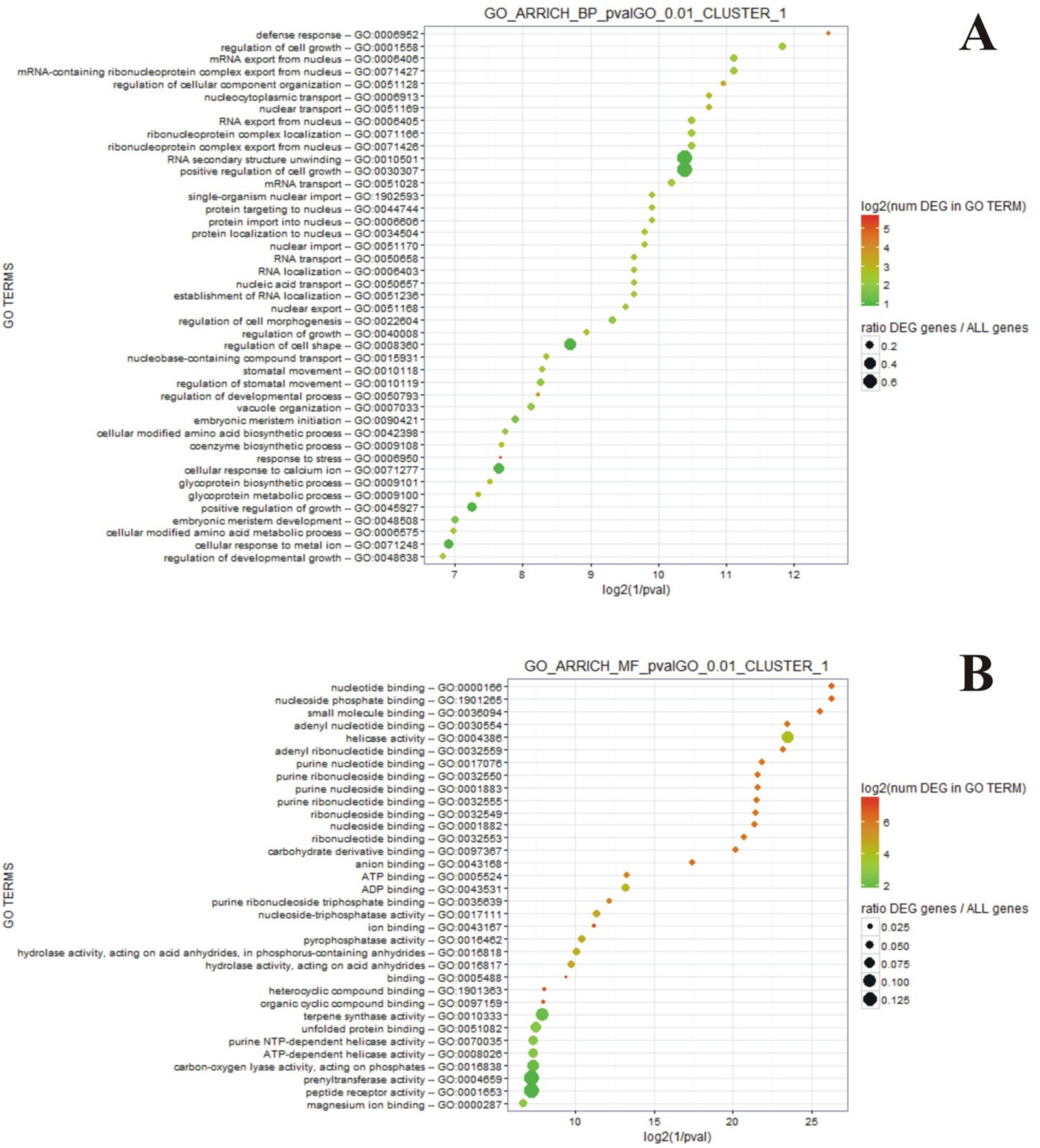

Supplemental Figure S20. GO term enrichment analysis of the largest cluster. (A)- Enrichment in terms of the Biological Process domain. (B)- Enrichment in terms of the Molecular Function domain. The GO terms the cluster is enriched in are reported on the y-axis, whereas the transformed GO enrichment probability is reported on the x-axis (higher values of the log_2(1/probability value) transformation correspond to higher significance). In addition, the colour of each spot indicates the number of DEGs of the cluster that matched the GO term (log_2-transformed), whereas the size of each spot shows the proportion of DEGs of the cluster that matched the GO term with respect to all the rice genes that pertain to that GO term.

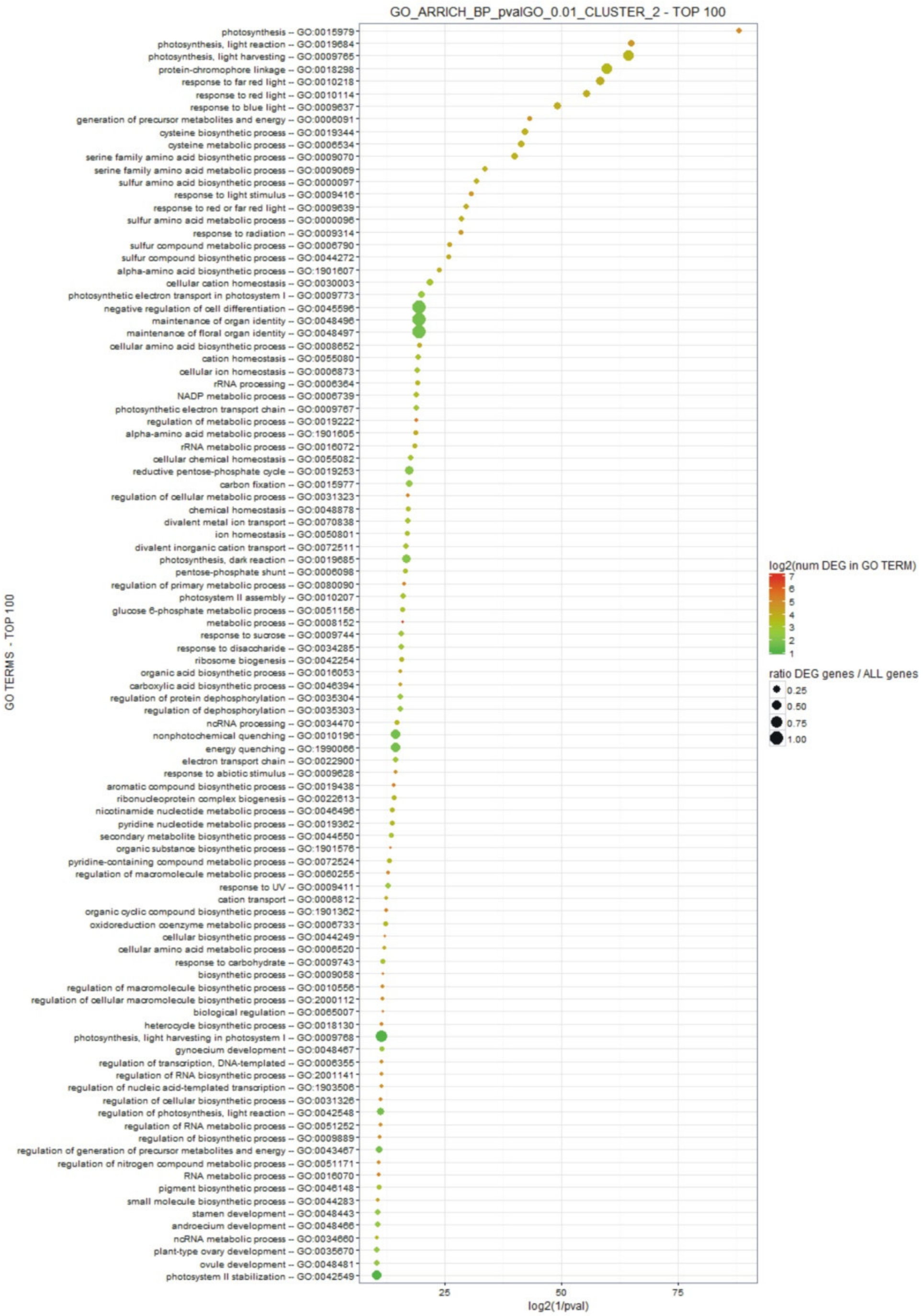

Supplemental Figure S21. GO term enrichment analysis of the second largest cluster for the Biological Process domain. The GO terms the cluster is enriched in are reported on the y-axis, whereas the transformed GO enrichment probability is reported on the x-axis (higher values of the $\log_2$(1/probability value) transformation correspond to higher significance). In addition, the colour of each spot indicates the number of DEGs of the cluster that matched the GO term ($\log_2$-transformed), whereas the size of each spot shows the proportion of DEGs of the cluster that matched the GO term with respect to all the rice genes that pertain to that GO term.

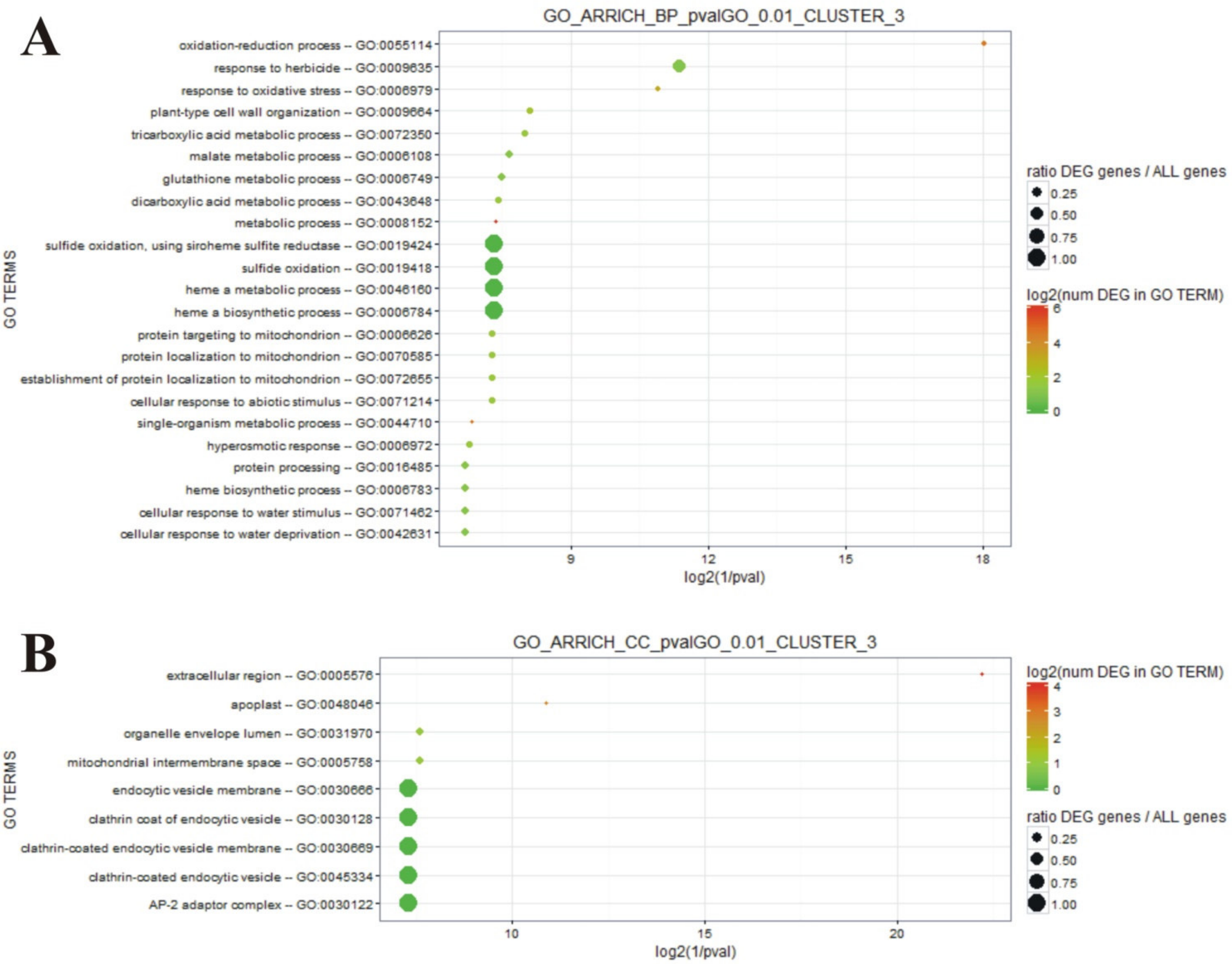

Supplemental Figure S22. GO term enrichment analysis of the third largest cluster. (A)- Enrichment in terms of the Biological Process domain. (B)- Enrichment in terms of the Cell Component domain. The GO terms the cluster is enriched in are reported on the y-axis, whereas the transformed GO enrichment probability is reported on the x-axis (higher values of the log_2(1/probability value) transformation correspond to higher significance). In addition, the colour of each spot indicates the number of DEGs of the cluster that matched the GO term (log_2-transformed), whereas the size of each spot shows the proportion of DEGs of the cluster that matched the GO term with respect to all the rice genes that pertain to that GO term.

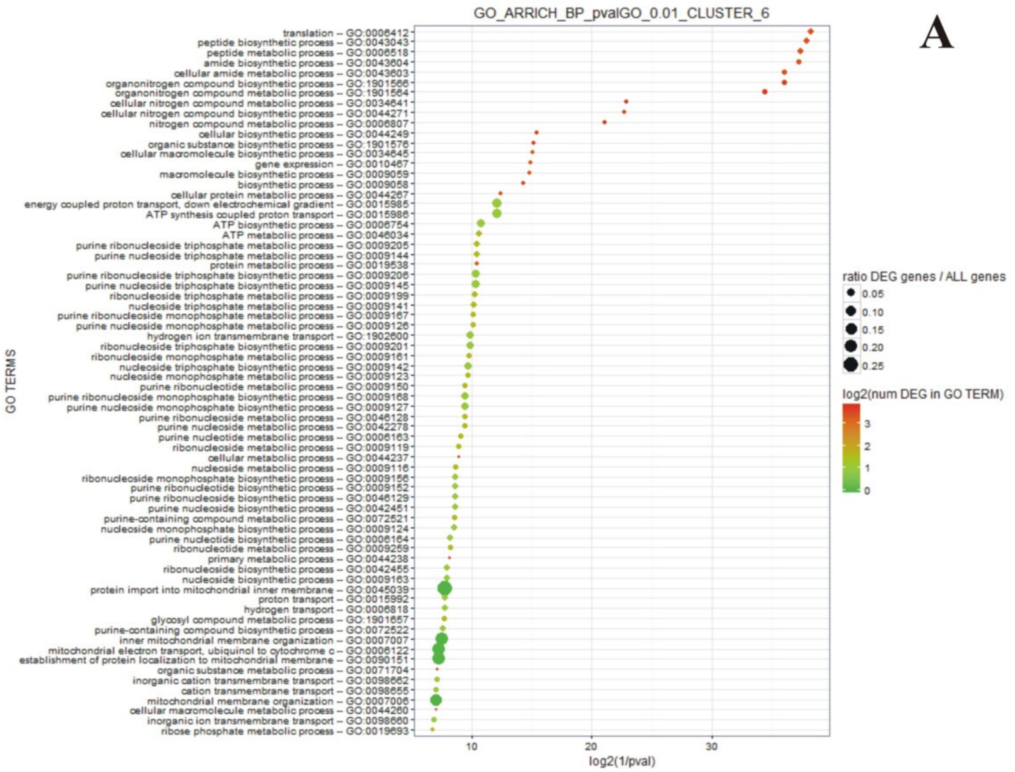

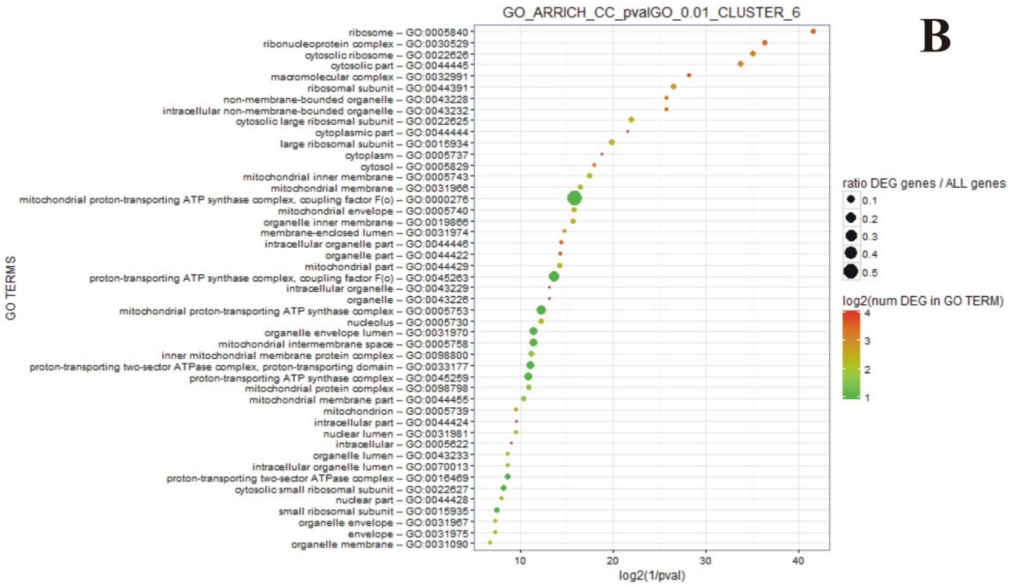

Supplemental Figure S23. GO term enrichment analysis of the sixth largest cluster. (A)- Enrichment in terms of the Biological Process domain. (B)- Enrichment in terms of the Cell Component domain. The GO terms the cluster is enriched in are reported on the y-axis, whereas the transformed GO enrichment probability is reported on the x-axis (higher values of the log_2(1/probability value) transformation correspond to higher significance). In addition, the colour of each spot indicates the number of DEGs of the cluster that matched the GO term (log_2-transformed), whereas the size of each spot shows the proportion of DEGs of the cluster that matched the GO term with respect to all the rice genes that pertain to that GO term.

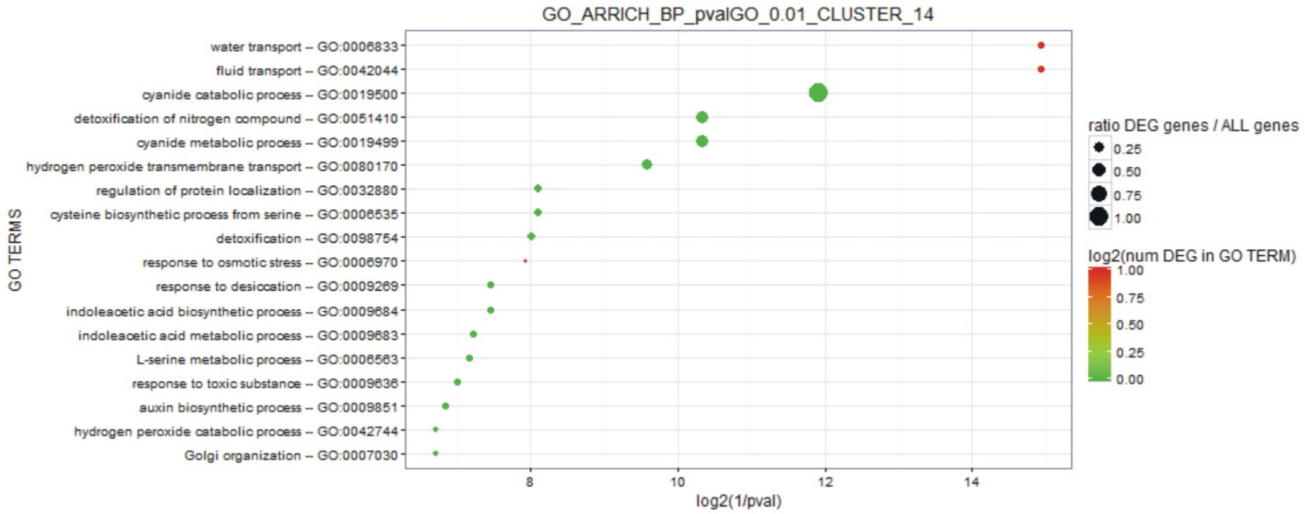

Supplemental Figure S24. GO term enrichment analysis of the 14th largest cluster for the Biological Process domain. The GO terms the cluster is enriched in are reported on the y-axis, whereas the transformed GO enrichment probability is reported on the x-axis (higher values of the $\log_2$(1/probability value) transformation correspond to higher significance). In addition, the colour of each spot indicates the number of DEGs of the cluster that matched the GO term ($\log_2$-transformed), whereas the size of each spot shows the proportion of DEGs of the cluster that matched the GO term with respect to all the rice genes that pertain to that GO term.
