# Supplementary material for: Seed Dormancy Involves a Transcriptional Program That Supports Early Plastid Functionality during Imbibition
Source: Plants (Basel). 2018 Apr 19;7(2):35. doi: 10.3390/plants7020035 (PMC6026906; doi:10.3390/plants7020035)
Supplement: Supplementary file 1 [file plants-07-00035-s001.zip › plants-273866-supplementary-update/Supplemental Tables.pdf]

SUPPLEMENTAL TABLES

Supplemental Table S1. Alignment of a whole set of reads (14,326,619 from a replicate of dormant red rice seeds incubated at 30 °C for 8 d), with publicly available genome sequences of *Oryza* species.

**Species**	**Overall read mapping rate**	**Number of multiple alignments**
Oryza sativa ssp *japonica*	97.5%	1,670,920 (12.0%) have multiple alignments (3202 have >20)
Oryza rufipogon	95.1%	873,313 (6.4%) have multiple alignments (0 have >20)
Oryza sativa ssp *indica*	94.6%	2,097,638 (15.5%) have multiple alignments (204 have >20)
Oryza nivara	93.6%	954,611 (7.1%) have multiple alignments (0 have >20)
Oryza barthii	89.8%	1,081,042 (8.4%) have multiple alignments (0 have >20)
Oryza glumaepatula	89.0%	657,466 (5.2%) have multiple alignments (0 have >20)
Oryza glaberrima	82.1%	1,299,343 (11.0%) have multiple alignments (4 have >20)
Oryza longistaminata	75.9%	833,692 (7.7%) have multiple alignments (0 have >20)
Oryza meridionalis	73.3%	961,661 (9.2%) have multiple alignments (0 have >20)
Oryza punctata	54.7%	1,129,289 (14.4%) have multiple alignments (9 have >20)
Oryza brachyantha	22.2%	312,783 (9.8%) have multiple alignments (0 have >20)

Supplemental Table S2. Most abundant mRNAs detected in imbibed caryopses across all six tested conditions. The most relevant GO (gene ontology) terms are shown.

Rank	Gene id.	Description of encoded product	Gene ontology
1	OS07G0206500 [1]	13 kDa prolamin	GO:0045735 nutrient reservoir activity
2	OS01G0762500 [1]	Glutelin type-A 1 (GluA-1)	GO:0045735 nutrient reservoir activity
3	OS07G0206400 [1]	13 kDa prolamin C	GO:0045735 nutrient reservoir activity
4	OS10G0400200 [1]	Glutelin type-A (GluA-2)	GO:0045735 nutrient reservoir activity
5	OS03G0766100 [1]	10 kDa prolamin	GO:0045735 nutrient reservoir activity
6	OS06G0507200 [1]	Prolamin	GO:0045735 nutrient reservoir activity
7	OS08G0104400 [1]	Putative uncharacterized protein	GO:0008150 biological_process GO:0003674 molecular_function
8	OS07G0214300 [1]	Seed allergenic protein RAG2 (albumin)	GO:0016068 type I hypersensitivity GO:0005615 extracellular space GO:0004867 serine-type endopeptidase inhibitor activity
9	OS07G0529800	Protein translation factor SUI1 homolog (eIF-1)	GO:0006413 translational initiation GO:0003743 translation initiation factor activity GO:0006417 regulation of translation
10	OS11G0582400 [1]	Antimicrobial peptide MBP-1 family	GO:0050832 defence response to fungus
11	OS02G0249600 [1]	Glutelin type B-2 (GluB-2)	GO:0045735 nutrient reservoir activity
12	OS05G0499100 [1]	19 kDa globulin (Glb1)	GO:0045735 nutrient reservoir activity
13	OS11G0703900	DnaK-type molecular chaperone hsp70	GO:0006950 response to stress GO:0005524 ATP binding
14	OS02G0453600 [1]	Glutelin (GluC)	GO:0045735 nutrient reservoir activity
15	OS02G0161900	Polyubiquitin 6	GO:0016874 ligase activity
16	OS12G0269200 [1]	Prolamin PPROL 17D	GO:0045735 nutrient reservoir activity
17	OS05G0349800	Embryonic abundant protein 1 (Late Embryogenesis Abundant protein OsEm)	GO:0048700 acquisition of desiccation tolerance in seed GO:0048316 seed development GO:0009737 response to abscisic acid
18	OS03G0793700	Cupin family protein; globulin-like (acts as zinc metalloprotease)	GO:0045735 nutrient reservoir activity
19	OS10G0542100 [1]	Class II metallothionein-like protein 1A	GO:0008270 zinc ion binding
20	OS07G0638300	1-Cys peroxiredoxin A (1Cys-Prx)	GO:0051920 peroxiredoxin activity GO:0016491 oxidoreductase activity
21	OS03G0663750	Not annotated	
22	OS03G0670700	Retrotransposon protein, putative, Ty1-copia subclass	GO:0003676 nucleic acid binding RNA secondary structure unwinding GO:0003690 double-stranded DNA binding GO:0032508 DNA duplex unwinding GO:0003697 single-stranded DNA binding GO:0006406 mRNA export from nucleus GO:0009737 response to abscisic acid GO:0016310 phosphorylation GO:0016301 kinase activity GO:0009507 chloroplast
23	OS04G0589800	OSJNBa0086O06.12 protein	GO:0009790 embryo development
24	OS07G0213800	Allergenic protein	GO:0016068 type I hypersensitivity GO:0005615 extracellular space GO:0004867 serine-type endopeptidase inhibitor activity
25	OS11G0660500	Translationally-controlled tumor protein homolog (TCTP)	GO:0040014 regulation of multicellular organism growth GO:0001558 regulation of cell growth GO:0048768 root hair cell tip growth GO:0009791 post-embryonic development GO:0007346 regulation of mitotic cell cycle GO:0008283 cell proliferation GO:0010252 auxin homeostasis GO:0009579 thylakoid GO:0009507 chloroplast
26	OS05G0542500	Late embryogenesis abundant protein, group 3 (putative dehydrin OsLEA3-1)	
27	OS07G0214600 [1]	Allergen RA16	GO:0016068 type I hypersensitivity GO:0005615 extracellular space GO:0004867 serine-type endopeptidase inhibitor activity
28	OS01G0256500	Zinc inducible protein-like	GO:0006950 response to stress
29	OS11G0454300	Water stress-inducible protein Rab21 (OsRab16A)	GO:0009415 response to water GO:0006950 response to stress
30	OS07G0215500 [1]	Seed allergenic protein RA5	GO:0016068 type I hypersensitivity GO:0005615 extracellular space GO:0004867 serine-type endopeptidase inhibitor activity
31	OS05G0140800	Glucose and ribitol dehydrogenase homolog	GO:0016491 oxidoreductase activity
32	OS04G0510900 [1]	Plant seed peroxygenase	GO:0071614 linoleic acid epoxygenase activity GO:0034389 lipid particle organization GO:0031407 oxylipin metabolic process GO:0004392 heme oxygenase (decyclizing) activity GO:0031969 chloroplast membrane GO:0009737 response to abscisic acid GO:0016165 linoleate 13S-lipoxygenase activity GO:0031408 oxylipin biosynthetic process
33	OS01G0705200	WSI18 protein induced by water stress	
34	OS01G0905800	Fructose-bisphosphate aldolase	GO:0004332 fructose-bisphosphate aldolase activity
35	OS01G0124401 [1]	Bowman-Birk type bran trypsin inhibitor	GO:0004867 serine-type endopeptidase inhibitor activity
36	OS03G0427300 [1]	Glutelin type-A 3 (GluA-3)	GO:0045735 nutrient reservoir activity
37	OS11G0211800 [1]		GO:0008200 ion channel inhibitor activity
38	OS02G0586900	Putative uncharacterized protein	
39	OS02G0249000 [1]	Glutelin C / B-2-type (GluD-1)	GO:0045735 nutrient reservoir activity
40	OS05G0569500	Putative embryo-specific protein Ose731	GO:0009737 response to abscisic acid
41	OS06G0612800	Zinc finger A20 and AN1 domain-containing stress-associated protein 8	GO:0003677 DNA binding
42	OS05G0489600	ADP-ribosylation factor 2	GO:0007264 small GTPase mediated signal transduction GO:0005525 GTP binding GO:0006471 protein ADP-ribosylation
43	OS12G0464400	Oxidoreductase, short chain dehydrogenase/reductase family protein	GO:0016491 oxidoreductase activity

[1] Genes that contain the AAATAA motif in their 3’ UTR sequence.

Supplemental Table S3. Most intensely expressed genes detected in imbibed caryopses (first 12 genes for each condition).

Condition	Incubation temperature (°C)	Time of incubation	Most expressed transcripts		
			Gene id.	Description (short)	Gene ontology (short)
Dormant	30	8 hours	OS01G0762500	Glutelin type-A 1	GO:0045735 nutrient reservoir activity
			OS07G0206500	13 kDa prolamin	GO:0045735 nutrient reservoir activity
			OS10G0400200	Glutelin type-A 2	GO:0045735 nutrient reservoir activity
			OS07G0206400	13 kDa prolamin C	GO:0045735 nutrient reservoir activity
			OS03G0766100	10 kDa prolamin	GO:0045735 nutrient reservoir activity
			OS06G0507200	Prolamin	GO:0045735 nutrient reservoir activity
			OS07G0214300	Seed allergenic protein RAG2	GO:0016068 type I hypersensitivity GO:0005615 extracellular space GO:0004867 serine-type endopeptidase inhibitor activity
			OS02G0249600	Glutelin type-B 2	GO:0045735 nutrient reservoir activity
			OS11G0582400	Embryo-specific protein	GO:0050832 defense response to fungus
			OS02G0453600	Glutelin	GO:0045735 nutrient reservoir activity
			OS05G0499100	19 kDa globulin	GO:0045735 nutrient reservoir activity
			OS03G0197300	protein	GO:0045735 nutrient reservoir activity
		8 days	OS02G0161900	Polyubiquitin 6	GO:0016874 ligase activity GO:0005737 cytoplasm
			OS11G0703900	DnaK-type molecular chaperone hsp70-rice; Heat shock cognate 70 kDa protein	GO:0006950 response to stress GO:0000166 nucleotide binding GO:0005524 ATP binding
			OS02G0586900	Putative uncharacterized protein	
			OS08G0104400	Putative uncharacterized protein	GO:0008150 biological_process GO:0003674 molecular_function
			OS07G0529800	Protein translation factor SUI1 homolog	GO:0006413 translational initiation GO:0003743 translation initiation factor activity GO:0006417 regulation of translation
			OS11G0660500	Translationally-controlled tumor protein homolog	GO:0040014 regulation of multicellular organism growth GO:0001558 regulation of cell growth GO:0009791 post-embryonic development GO:0007346 regulation of mitotic cell cycle GO:0008283 cell proliferation GO:0009790 embryo development GO:0048364 root development GO:0010252 auxin homeostasis GO:0009579 thylakoid GO:0048046 apoplast GO:0009507 chloroplast GO:0005886 plasma membrane GO:0005634 nucleus GO:0005737 cytoplasm
			OS07G0206500	13 kDa prolamin	GO:0045735 nutrient reservoir activity
			OS03G0670700	Retrotransposon protein, putative, Ty1-copia subclass	GO:0003676 nucleic acid binding GO:0000166 nucleotide binding GO:0010501 RNA secondary structure unwinding GO:0003690 double-stranded DNA binding GO:0032508 DNA duplex unwinding GO:0003697 single-stranded DNA binding GO:0006406 mRNA export from nucleus GO:0009737 response to abscisic acid GO:0005730 nucleolus GO:0005618 cell wall GO:0005777 peroxisome GO:0016310 phosphorylation GO:0016301 kinase activity GO:0007623 circadian rhythm GO:0009507 chloroplast GO:0005886 plasma membrane GO:0005737 cytoplasm GO:0005634 nucleus
			OS07G0206400	13 kDa prolamin C	GO:0045735 nutrient reservoir activity
			OS01G0762500	Glutelin type-A 1	GO:0045735 nutrient reservoir activity
			OS02G0465900	protein; Putative OsCTTP	GO:0010288 response to lead ion
			OS01G0256500	Zinc inducible protein	GO:0006950 response to stress
	10	8 hours	OS07G0206500	13 kDa prolamin	GO:0045735 nutrient reservoir activity
			OS01G0762500	Glutelin type-A 1	GO:0045735 nutrient reservoir activity
			OS10G0400200	Glutelin type-A 2	GO:0045735 nutrient reservoir activity
			OS07G0206400	13 kDa prolamin C	GO:0045735 nutrient reservoir activity
			OS03G0766100	10 kDa prolamin	GO:0045735 nutrient reservoir activity
			OS06G0507200	prolamin	GO:0045735 nutrient reservoir activity
			OS11G0582400	Embryo-specific protein	GO:0050832 defense response to fungus
			OS07G0214300	Seed allergenic protein RAG2	GO:0016068 type I hypersensitivity GO:0005615 extracellular space GO:0004867 serine-type endopeptidase inhibitor activity
			OS05G0499100	19 kDa globulin	GO:0045735 nutrient reservoir activity
			OS02G0249600	Glutelin type-B 2	GO:0045735 nutrient reservoir activity
			OS03G0793700	Cupin family protein, expressed; Globulin protein	GO:0045735 nutrient reservoir activity
			OS02G0453600	Glutelin	GO:0045735 nutrient reservoir activity
		8 days	OS07G0206500	13 kDa prolamin	GO:0045735 nutrient reservoir activity
			OS01G0762500	Glutelin type-A 1	GO:0045735 nutrient reservoir activity
			OS10G0400200	Glutelin type-A 2	GO:0045735 nutrient reservoir activity
			OS07G0206400	13 kDa prolamin C	GO:0045735 nutrient reservoir activity
			OS03G0766100	10 kDa prolamin	GO:0045735 nutrient reservoir activity
			OS06G0507200	prolamin	GO:0045735 nutrient reservoir activity
			OS07G0214300	Seed allergenic protein RAG2	GO:0016068 type I hypersensitivity GO:0005615 extracellular space GO:0004867 serine-type endopeptidase inhibitor activity
			OS05G0349800	Embryonic abundant protein 1	GO:0048700 acquisition of desiccation tolerance in seed GO:0048316 seed development GO:0009737 response to abscisic acid
			OS05G0499100	19 kDa globulin	GO:0045735 nutrient reservoir activity
			OS02G0249600	Glutelin type-B 2	GO:0045735 nutrient reservoir activity
			OS03G0663750		
			OS02G0453600	Glutelin	GO:0045735 nutrient reservoir activity
Nondormant	30	8 hours	OS07G0206500	13 kDa prolamin	GO:0045735 nutrient reservoir activity
			OS01G0762500	Glutelin type-A 1	GO:0045735 nutrient reservoir activity
			OS10G0400200	Glutelin type-A 2	GO:0045735 nutrient reservoir activity
			OS07G0206400	13 kDa prolamin C	GO:0045735 nutrient reservoir activity
			OS03G0766100	10 kDa prolamin	GO:0045735 nutrient reservoir activity
			OS07G0214300	Seed allergenic protein RAG2	GO:0016068 type I hypersensitivity GO:0005615 extracellular space GO:0004867 serine-type endopeptidase inhibitor activity
			OS06G0507200	prolamin	GO:0045735 nutrient reservoir activity
			OS07G0529800	Protein translation factor SUI1 homolog	GO:0006413 translational initiation GO:0003743 translation initiation factor activity GO:0006417 regulation of translation
			OS06G0681400	Polyubiquitin	GO:0016874 ligase activity GO:0005737 cytoplasm
			OS05G0499100	19 kDa globulin	GO:0045735 nutrient reservoir activity
			OS02G0161900	Polyubiquitin 6	GO:0016874 ligase activity GO:0005737 cytoplasm
			OS08G0104400	uncharacterized protein	GO:0008150 biological_process GO:0003674 molecular_function
	10	8 days	OS07G0206500	13 kDa prolamin	GO:0045735 nutrient reservoir activity
			OS01G0762500	Glutelin type-A 1	GO:0045735 nutrient reservoir activity
			OS08G0104400	protein	GO:0008150 biological_process GO:0003674 molecular_function
			OS07G0206400	13 kDa prolamin C	GO:0045735 nutrient reservoir activity
			OS10G0400200	Glutelin type-A 2	GO:0045735 nutrient reservoir activity
			OS07G0214300	Seed allergenic protein RAG2	GO:0016068 type I hypersensitivity GO:0005615 extracellular space GO:0004867 serine-type endopeptidase inhibitor activity
			OS11G0582400	Embryo-specific protein	GO:0050832 defense response to fungus
			OS03G0766100	10 kDa prolamin	GO:0045735 nutrient reservoir activity
			OS06G0507200	prolamin	GO:0045735 nutrient reservoir activity
			OS07G0529800	Protein translation factor SUI1 homolog	GO:0006413 translational initiation GO:0003743 translation initiation factor activity GO:0006417 regulation of translation
			OS03G0793700	Cupin family protein; Globulin	GO:0045735 nutrient reservoir activity
			OS05G0349800	Embryonic abundant protein 1	GO:0048700 acquisition of desiccation tolerance in seed GO:0048316 seed development GO:0009737 response to abscisic acid

Supplemental Table S4. MapMan “transcription” sub-BINs for D 8 h 30 °C *vs* ND 8 h 30 °C, Wilcoxon Rank Sum Test with Benjamini and Hochberg correction.

BIN	Name	Elements	P-value
27.3.24	RNA.regulation of transcription.MADS box transcription factor family	11	3.149 E-4
27.3.37	RNA.regulation of transcription.AS2,Lateral Organ Boundaries Gene Family	8	0.002
27.3.6	RNA.regulation of transcription.bHLH,Basic Helix-Loop-Helix family	26	0.016
27.3.3	RNA.regulation of transcription.AP2/EREBP, APETALA2/Ethylene-responsive element binding protein family	33	0.016
27.3.7	RNA.regulation of transcription.C2C2(Zn) CO-like, Constans-like zinc finger family	13	0.084
27.3.32	RNA.regulation of transcription.WRKY domain transcription factor family	14	0.104
27.3.20	RNA.regulation of transcription.G2-like transcription factor family, GARP	6	0.110
27.3.44	RNA.regulation of transcription.Chromatin Remodeling Factors	6	0.165
27.3.8	RNA.regulation of transcription.C2C2(Zn) DOF zinc finger family	7	0.173
27.3.22	RNA.regulation of transcription.HB,Homeobox transcription factor family	14	0.260
27.3.63	RNA.regulation of transcription.PHD finger transcription factor	3	0.366
27.3.64	RNA.regulation of transcription.PHOR1	2	0.438
27.3.40	RNA.regulation of transcription.Aux/IAA family	6	0.538
27.3.60	RNA.regulation of transcription.NIN-like bZIP-related family	2	0.538
27.3.4	RNA.regulation of transcription.ARF, Auxin Response Factor family	6	0.581
27.3.69	RNA.regulation of transcription.SET-domain transcriptional regulator family	4	0.584
27.3.17	RNA.regulation of transcription.CPP(Zn),CPP1-related transcription factor family	2	0.616
28.1.3	DNA.synthesis/chromatin structure.histone	25	0.632
27.3.11	RNA.regulation of transcription.C2H2 zinc finger family	13	0.649
27.3.65	RNA.regulation of transcription.Polycomb Group (PcG)	2	0.649
27.3.39	RNA.regulation of transcription.AtSR Transcription Factor family	3	0.649
27.3.67	RNA.regulation of transcription.putative transcription regulator	22	0.649
27.3.16	RNA.regulation of transcription.CCAAT box binding factor family, HAP5	3	0.764
27.3.14	RNA.regulation of transcription.CCAAT box binding factor family, HAP2	3	0.764
27.3.36	RNA.regulation of transcription.Argonaute	1	0.764
27.3.5	RNA.regulation of transcription.ARR	1	0.767
27.3.9	RNA.regulation of transcription.C2C2(Zn) GATA transcription factor family	2	0.767
27.3.49	RNA.regulation of transcription.GeBP like	2	0.780
27.3.57	RNA.regulation of transcription.JUMONJI family	4	0.782
27.3.59	RNA.regulation of transcription.Methyl binding domain proteins	1	0.791
27.3.30	RNA.regulation of transcription.Trihelix, Triple-Helix transcription factor family	2	0.799
27.3.35	RNA.regulation of transcription.bZIP transcription factor family	11	0.803
27.1.20	RNA.processing.degradation dicer	1	0.837
27.3.25	RNA.regulation of transcription.MYB domain transcription factor family	24	0.864
27.3.48	RNA.regulation of transcription.FHA transcription factor	1	0.866
27.3.10	RNA.regulation of transcription.C2C2(Zn) YABBY family	3	0.906
27.3.71	RNA.regulation of transcription.SNF7	2	0.906
27.3.47	RNA.regulation of transcription.ELF3	1	0.910
27.3.19	RNA.regulation of transcription.EIN3-like(EIL) transcription factor family	2	0.910
27.3.50	RNA.regulation of transcription.General Transcription	3	0.941
27.3.1	RNA.regulation of transcription.ABI3/VP1-related B3-domain-containing transcription factor family	2	0.941
27.3.99	RNA.regulation of transcription.unclassified	37	0.944
27.3.52	RNA.regulation of transcription.Global transcription factor group	1	0.944
27.3.55	RNA.regulation of transcription.HDA	3	0.944
27.3.23	RNA.regulation of transcription.HSF,Heat-shock transcription factor family	6	0.944
27.3.70	RNA.regulation of transcription.Silencing Group	1	0.944
27.3.26	RNA.regulation of transcription.MYB-related transcription factor family	4	0.944
27.3.27	RNA.regulation of transcription.NAC domain transcription factor family	2	0.946
17.3.2.2	hormone metabolism.brassinosteroid.signal transduction.BZR	1	0.950
27.3.21	RNA.regulation of transcription.GRAS transcription factor family	3	0.971
27.3.51	RNA.regulation of transcription.General Transcription, TBP-binding protein	1	0.983
27.3.12	RNA.regulation of transcription.C3H zinc finger family	6	0.988

**Supplemental Table S5. Primers used in this study for qPCR.**		
**Gene**	**RAP ID**	**Primers sequences 5' to 3'**
EXPA4	Os05g0477600	CACTGCCAAGCTCCGCTTCAA<br>TGCTGGTGATGGTGTCTAAACG
SAU31<br>(Jain et al., 2006)	Os08g0118500	CCACCACTAGCTCGCTGTTA<br>CACAAAAATCCGAGCCGTCA
OPR	Os06g0216300	TTCGATCGAGAAGAACCAAAGC<br>CCACTTTCGCCGGTGCGTG
OsTOR	Os05g0235300	ATATCAGACGCCGTGCCATT<br>ATGAGTACCAACCTGCACCG
OsSAP11	Os08g0504700	TCCCAAAAGCAAAGCGAACC<br>CGCCATGATCGAATCCGAGA
ABI4<br>(Wang et al., 2015)	Os05g0351200	ACCCCTGGTTCGATCTCTTC<br>AGCTGGAACGCCAAGCTAAG
Edf<br>(Wang et al., 2016)	Os08g0366100	TCCGAACCAGCAGATCATCG<br>GCATGGTATCAAAAGACCCAGC
